# Synthesis and Antiviral and Antitumor Activities of Novel 18*β*-Glycyrrhetinic Acid Derivatives

**DOI:** 10.3390/ijms241915012

**Published:** 2023-10-09

**Authors:** Bo-Wen Pan, Liang-Liang Zheng, Yang Shi, Zhang-Chao Dong, Ting-Ting Feng, Jian Yang, Ying Wei, Ying Zhou

**Affiliations:** 1 College of Pharmacy, Guizhou University of Traditional Chinese Medicine, Guiyang 550025, China; bwpan1105@163.com (B.-W.P.); zx15117508142@163.com (L.-L.Z.); xiaoyewater@163.com (Y.S.); dongzhangchao0824@163.com (Z.-C.D.); ftt0809@163.com (T.-T.F.); 2 College of Pharmacy and Nutrition, University of Saskatchewan, Saskatoon, SK S7N 5E5, Canada; jian.yang@usask.ca

**Keywords:** 18*β*-glycyrrhetinic acid, synthesis of derivatives, antiviral activities, antitumor activities

## Abstract

A series of novel derivatives of 18*β*-glycyrrhetinic acid (GA) were synthesized by introducing aromatic or heterocyclic structures to extend the side chain, thereby enhancing their interaction with amino acid residues in the active pocket of the target protein. These compounds were structurally characterized using ^1^H NMR, ^13^C NMR, and HRMS. The compounds were subsequently evaluated for their inhibitory effects on HIV-1 protease and cell viability in the human cancer cell lines K562 and HeLa and the mouse cancer cell line CT26. Towards HIV-1 protease, compounds **28** and **32**, which featured the introduction of heterocyclic moieties at the C3 position of GA, exhibited the highest inhibition, with inhibition rates of 76% and 70.5%, respectively, at 1 mg/mL concentration. Further molecular docking suggests that a 3-substituted polar moiety would be likely to enhance the inhibitory activity against HIV-1 protease. As for the anti-proliferative activities of the GA derivatives, incorporation of a thiazole heterocycle at the C3- position in compound **29** significantly enhanced the effect against K562 cells with an IC_50_ value of 8.86 ± 0.93 µM. The introduction of electron-withdrawing substituents on the C3-substituted phenyl ring augmented the anti-proliferative activity against Hela and CT26 cells. Compound **13** exhibited the highest inhibitory activity against Hela cells with an IC_50_ value of 9.89 ± 0.86 µM, whereas compound **7** exerted the strongest inhibition against CT26 cells with an IC_50_ value of 4.54 ± 0.37 µM. These findings suggest that further modification of GA is a promising path for developing potent novel anti-HIV and anticancer therapeutics.

## 1. Introduction

Licorice (*Glycyrrhiza glabra*) is a medicinal plant widely used in traditional Chinese medicine (TCM). Its major active ingredient, glycyrrhetinic acid (GA), is a pentacyclic triterpenoid with a broad spectrum of pharmacological activities [1,2,3]. GA has been shown to possess anticancer [4,5,6,7], antiviral [8,9,10], antioxidant [11], antibacterial [12], and anti-inflammatory [13] functions. Especially, its anticancer and antiviral functions have attracted extensive research attention in recent years. Various types of modification of GA have been undertaken to improve its pharmacological activities and boost its application in complementary and alternative medicine (CAM).

Human immunodeficiency virus (HIV), which has emerged to be a sustained global epidemic, has infected approximately 79.3 million and killed 36.3 million people so far [14,15,16]. To combat HIV infection, antiretroviral therapy (ART), which is a cocktail combination of reverse transcriptase inhibitors [17], protease inhibitors [18], and/or integrase inhibitors [19,20], has been developed and shown to be effective. However, drug resistance arises rapidly towards these therapeutics, and the continuous search for novel anti-HIV agents is critical for improving patient survival and patients’s quality of life.

Besides HIV infection, cancer is another serious disease threatening human health. Globally, it is the second leading cause of death, only after cardiovascular diseases [21]. Despite the large number of anticancer therapies that have been developed, cancer heterogeneity [22] and the rapid development of resistance towards the therapeutics [23] have imposed a big challenge to therapeutic efficacy and patient survival. Thus, similar to HIV infection, identifying and advancing novel anticancer treatments is still of high importance.

As a valuable resource, natural products possess a series of desirable attributes for drug development, including biodegradability, wide availability, and a reduced likelihood of drug resistance [24,25,26]. In terms of licorice, its active ingredient, glycyrrhizic acid (GL), enhanced the level of HIV-1 p24 antigen in patients with hemophilia and AIDS [27]. GL is also able to stimulate interferon production, augment the activity of natural killer cells, and boost the count of CD4-positive T lymphocytes, which, in turn, would impede AIDS progression [28,29]. All these observations imply that GA, the primary hydrolytic metabolite of GL [30], is potentially capable of suppressing HIV-1 replication *in vivo*. Furthermore, GA was shown to exhibit selective cytotoxicity towards various cancer cells, including breast, ovarian, leukemia, liver, and gastric cancer cells [31,32,33], making it a promising candidate for designing and developing novel anticancer agents. GA can exert its cytotoxicity via diverse mechanisms, such as disruption of the actin cytoskeleton, suppression of the p38 MAPK-AP1 signaling axis, and DNA fragmentation-mediated apoptosis [34,35,36]. The inherent biological activities of natural compounds are usually moderate against specific targets [37]. Therefore, in this study, we have developed a series of 32 novel GA derivatives by introducing aromatic or heterocyclic structures to extend the side chains, thereby enhancing their interaction with the target proteins and improving their antiviral and anticancer activities.

## 2. Results and Discussion

### 2.1. Synthesis of GA Derivatives

**Scheme 1:**
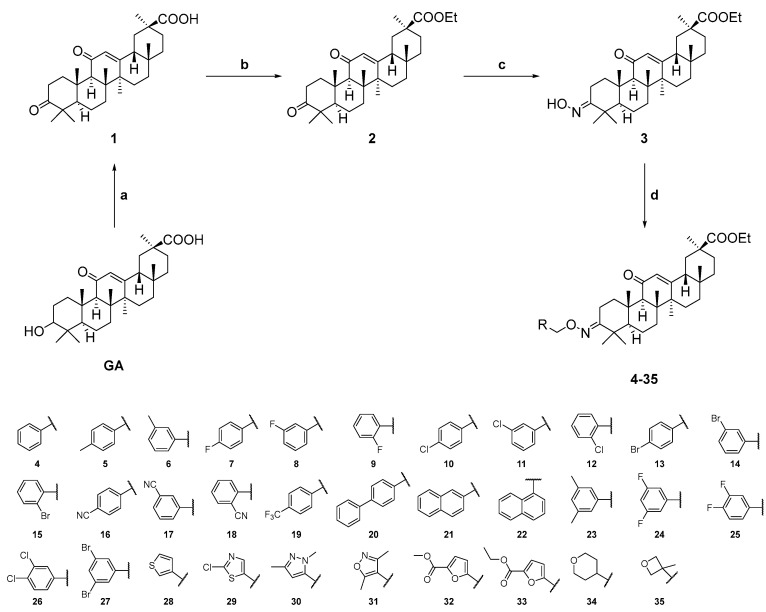
Synthesis of compounds 1–35. Reagents and conditions: a. Dess-Martin Periodinane, DCM, rt, 3 h. b. K_2_CO_3_, KI, DMF, C_2_H_5_Br, rt, 4 h. c. NH_2_OH·HCl, NaHCO_3_, C_2_H_5_OH, 90 °C, 14 h, 80%. d. Halogenated hydrocarbons, NaH, Anhydrous THF, rt.

**Figure 1:**
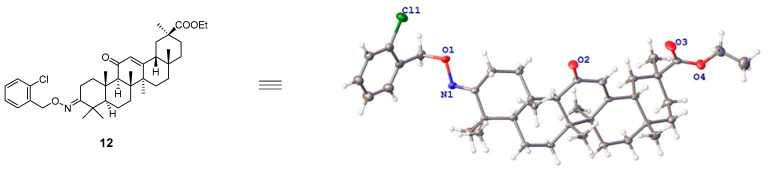
Structure of compound **12** determined by X-ray crystallography.

The chemical synthesis of GA derivatives is presented in Scheme 1. Briefly, with GA as the starting substance, its 3-hydroxyl group was oxidized into a carbonyl group by Dess-Martin Periodinane, resulting in compound **1** (white solid, yield 99%). Compound **1** was esterized by reacting with CH_3_CH_2_Br, using K_2_CO_3_ and KI as catalysts, to form compound **2** (white solid, yield 64%). Compound **2** was subsequently treated with NH_2_OH·HCl and NaHCO_3_, converting the 3-carbonyl group into an oxime group to generate the intermediate compound **3** (white solid, yield 80%). Finally, compound **3** underwent nucleophilic substitution reactions with various halogenated hydrocarbons under the catalysis of NaH to form a series of GA derivatives (compounds 4–35). The structures of all compounds were characterized by ^1^H NMR, ^13^C NMR, and HRMS. The stereostructure of compound **12** was further confirmed by X-ray crystallography (Figure 1 and Tables S1–S7 of the Supporting Information). Crystallographic data for compound **12** has been deposited in the Cambridge Crystallographic Data Centre (deposition number: CCDC 2287206).

### 2.2. In Vitro Inhibitory Activity against HIV-1 Protease

Compounds **4–35** were assayed for their inhibitory activities against HIV-1 protease. As shown in Table 1, the inhibitory effect was mild to moderate except for compounds **7**, **28,** and **32**. Compound **28**, which has a 3-thiophene group, displayed the highest activity with an inhibition rate of 76% at 1 mg/mL concentration. Compound **32**, which has a 3-furan moiety, exhibited comparable activity to compound **28** with an inhibition rate of 70.5%. Compound **7** with a para-fluorophenyl at 3-position exhibited weaker activity than compounds **28** and **32** with an inhibition rate of 53.6%, but was the strongest among compounds with a 3-phenyl moiety with different substitutions.

### 2.3. Molecular Docking

**Figure 2:**
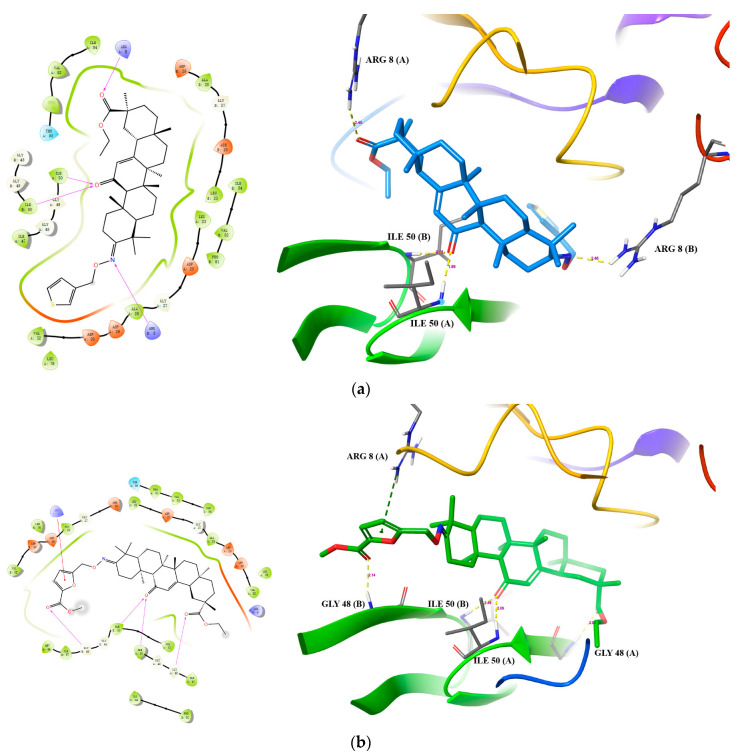
Putative binding of compound **28** (**a**) and compound **32** (**b**) to HIV-1 protease. Carbon atoms were colored blue (**a**) or green (**b**), while oxygen, hydrogen, nitrogen, and sulfur atoms were colored red, grey, blue, and yellow, respectively. A green dotted line was used to show the π-cation interaction, and hydrogen bonds were shown as yellow dotted lines.

To get a glimpse of potential interactions between the most active GA derivatives and HIV-1 protease, we docked compounds **28** and **32** into the active site of HIV-1 protease (PDB ID: 1QBS), respectively (Table 2). Compound **28** gave a docking score of −7.236 and a binding energy of −53.102 kcal/moL. It formed four hydrogen bonds with residues Ile 50(A), Ile 50(B), Arg 8(A), and Arg 8(B) of HIV-1 protease, with respective bond lengths of 1.89 Å, 2.18 Å, 2.45 Å, and 2.46 Å (Figure 2a). Compound **32** gave a lower docking score of -6.834 but a better binding energy of −64.549 kcal/moL. It formed four hydrogen bonds with residues Ile 50(A), Ile 50(B), Gly 48(A), and Gly 48(B) of HIV-1 protease, with respective bond lengths of 2.09 Å, 2.49 Å, 2.03 Å, and 2.14 Å (Figure 2b). It also formed a π-cation interaction with residue Arg 8(A). The polar interactions between the 3-substitue group of compounds **28** and **32** and HIV-1 protease imply that a 3-substitute hydrophobic group, such as a phenyl group, would be unfavorable for interactions with HIV-1 protease, which would render inhibitory activity lower.

### 2.4. In Vitro effect on Viability of Cancer Cells

To identify whether the GA derivatives could be potentially used to treat human or animal tumors, their effects on the viability of human chronic myeloid leukemia cell lines K562, human cervical cancer cell lines Hela, and mouse colon cancer cell lines CT26 were evaluated using the MTT assay. The IC_50_ values of the compounds were summarized in Table 3. In human leukemia K562 cells, five compounds (**4**, **7**, **8**, **28,** and **29**) exhibited stronger inhibition on cell growth than the positive control cisplatin, with compound **29** having the best inhibitory activity (IC_50_ = 8.86 ± 0.93 µM). In human cervical cancer HeLa cells, four compounds (**13**, **24**, **30,** and **31**) showed more potent activity than cisplatin, with compound **13** having the highest activity (IC_50_ = 9.89 ± 0.86 µM). As for the mouse colon CT26 cells, three compounds (**7**, **8,** and **31**) manifested a better inhibitory effect than cisplatin, with compound **7** showcasing the highest activity (IC_50_ = 4.54 ± 0.37 µM). Importantly, the IC_50_ values for the most effective compounds (7, 13, and 29) against normal NCM460 cells were all greater than 100 µM. These results suggest that GA derivatives with 3-substituted heterocyclic moieties possess inhibitory activities against human and animal cancer cells, and electron-withdrawing groups, particularly fluorine, on the 3-phenyl moiety render stronger effects.

## 3. Materials and Methods

### 3.1. General Experimental Procedures

The progress of the chemical reactions was monitored by thin-layer chromatography (TLC) under UV light. Compound purification was carried out using flash chromatography on silica gel. Chemical yields refer to pure, isolated substances. HRMS spectra of the compounds were measured on a Waters Xevo G2-S QTOF mass spectrometer (Waters, Massachusetts, USA) using the electron spray ionization (ESI) method. ^1^H, ^13^C, and ^19^F NMR spectra were obtained using a Bruker DPX-400 MHz spectrometer (Bruker, Billerica, Massachusetts, USA). Chemical shifts were reported in ppm from CDCl_3_ or TMS, with solvent resonance as an internal standard. The following abbreviations were used to designate chemical shift multiplicities: s = singlet, d = doublet, t = triplet, q = quartet, h = heptet, m = multiplet, and br = broad.

The HIV-1 protease kit (AS-71147) and HIV-1 protease (wild type, NC-001802) were purchased from AnaSpec (Fremont, CA, USA). Enzyme assays were conducted in 96- and 384-well plates using a Synergy II Microplate Reader (Biotec Co., Burlington, VT, USA).

### 3.2. Synthesis of the Compounds ***1**–**35***

Glycyrrhetinic acid (1 equiv) and Dess-Martin Periodinane (1 equiv) were dissolved in 20 mL of dichloromethane and stirred at room temperature for 3 h. Upon completion of the reaction (monitored by TLC), the reaction mixture was quenched with 20 mL of saturated sodium bicarbonate. The resulting mixture was then extracted three times with 20 mL of dichloromethane. The combined organic phases were dried using anhydrous sodium sulfate. After filtration, the solvent was evaporated to produce compound **1** (white solid) with a yield of 99%.

Compound **1** (1 equiv), K_2_CO_3_ (2 equiv), and KI (0.2 equiv) were dissolved in 20 mL of DMF. Then, C_2_H_5_Br (2 equiv) was added to the solution, and the mixture was stirred at room temperature for 4 h to complete the reaction (monitored by TLC). Upon completion, the reaction mixture was quenched with 20 mL of water and subsequently extracted three times with 20 mL of ethyl acetate. The organic phase was dried using anhydrous sodium sulfate and filtered. The solvent evaporated. The residue was purified by silica gel chromatography to give the white solid compound **2,** with a yield of 74%.

Compound **2** (1 equiv.) was dissolved in EtOH (50 mL). Then, NH_2_OH·HCl (1.5 equiv.) and NaHCO_3_ (1.5 equiv.) were added to reflux for 4 h. Upon reaction completion, the reaction mixture was concentrated under vacuum, and the residue was extracted with ethyl acetate and H_2_O. The organic layer was washed with saturated aqueous NaCl, dried with MgSO_4_, and concentrated under vacuum to give the crude residue, which was subsequently purified by column chromatography on silica gel to produce compound **3** in 85% yield.

Compound **3** (1 equiv.) was dissolved in 2 mL of dry THF, followed by the addition of NaH (1.5 equiv.). After stirring in an ice bath for 30 min, the halogenated hydrocarbon (2 equiv.) was added. The reaction mixture was allowed to proceed at room temperature for 4 h. Upon reaction completion, the reaction was quenched with 1 mL of methanol. The solvent was then evaporated, and the target compounds **4–35** were purified by column chromatography.

### 3.3. Compound Characterization

Product **4** was a white solid in 83% yield, Mp: 156–158 °C; ^1^H NMR (400 MHz, CDCl_3_) δ 7.38–7.31 (m, 4H), 7.30–7.25 (m, 1H), 5.65 (s, 1H), 5.06 (s, 2H), 4.23–4.08 (m, 2H), 2.30–2.94 (m, 1H), 2.81–2.76 (m, 1H), 2.36 (s, 1H), 2.32–2.24 (m, 1H), 2.13–2.08 (m, 1H), 2.07–1.97 (m, 2H), 1.94–1.89 (m, 1H), 1.82 (td, J = 13.6, 4.6 Hz, 1H), 1.76–1.57 (m, 4H), 1.53–1.38 (m, 3H), 1.34–1.30 (m, 2H), 1.34 (s, 3H), 1.26 (t, J = 7.1 Hz, 3H), 1.20 (s, 3H), 1.17 (s, 3H), 1.14 (s, 6H), 1.05–1.00 (m, 3H), 1.05 (s, 3H), 0.81 (s, 3H); ^13^C NMR (100 MHz, CDCl_3_) δ 199.94, 176.47, 169.62, 166.37, 138.68, 128.55, 128.26, 128.18, 127.52, 75.35, 61.42, 60.42, 55.55, 48.49, 45.45, 43.94, 43.38, 41.18, 40.33, 39.10, 37.82, 37.03, 32.48, 31.92, 31.21, 28.69, 28.42, 27.55, 26.57, 26.51, 23.53, 23.42, 18.70, 18.34, 18.21, 15.77, 14.44. HRMS (ESI): Exact mass calcd for C_39_H_55_NNaO_4_ [M+Na]^+^: 624.4023, Found: 624.4014.

Product **5** is a white solid in 86% yield, Mp: 145–147 °C; ^1^H NMR (400 MHz, CDCl_3_) δ 7.26– 7.13 (m, 2H), 7.14 (d, *J* = 7.8 Hz, 2H), 5.65 (s, 1H), 5.01 (s, 2H), 4.23–4.08 (m, 2H), 2.99–2.93 (m, 1H), 2.80–2.76 (m, 1H), 2.36 (s, 1H), 2.33 (s, 3H), 2.30–2.22 (m, 1H), 2.13–1.96 (m, 2H), 1.94–1.89 (m, 1H), 1.86–1.78 (m, 1H), 1.82 (td, *J* = 13.7, 4.7 Hz, 1H), 1.78–1.57L (m, 4H), 1.50–1.38 (m, 3H), 1.34–1.30 (m, 2H), 1.34 (s, 3H), 1.26 (t, *J* = 7.1 Hz, 3H), 1.20 (s, 3H), 1.17 (s, 3H), 1.14 (s, 6H), 1.06–1.00 (m, 3H), 1.06 (s, 3H), 0.81 (s, 3H). ^13^C NMR (100 MHz, CDCl_3_) δ 199.94, 176.46, 169.58, 166.15, 137.18, 135.54, 128.96, 128.54, 128.33, 75.26, 61.42, 60.41, 55.56, 48.49, 45.44, 43.93, 43.37, 41.17, 40.32, 39.08, 37.81, 37.02, 32.49, 31.91, 31.21, 28.68, 28.40, 27.53, 26.56, 26.50, 23.55, 23.40, 21.29, 18.69, 18.32, 18.17, 15.75, 14.43. HRMS (ESI): Exact mass calcd for C_40_H_57_NNaO_4_ [M+Na]^+^: 638.4180, Found: 638.4176.

Product **6** was a white solid in 98% yield, Mp: 114–116 °C; ^1^H NMR (400 MHz, CDCl_3_) δ 7.26–7.20 (m, 1H), 7.18–7.14 (m, 2H), 7.10–7.08 m, 1H), 5.65 (s, 1H), 5.03 (s, 2H), 4.23–4.08 (m, 2H), 2.99–2.93 (m, 1H), 2.81–2.75 (m, 1H), 2.37–2.25 (m, 2H), 2.35 (s, 3H), 2.13–2.08 (m, 1H), 2.07–1.97 (m, 2H), 1.94–1.89 (m, 1H), 1.83 (td, *J* = 13.6, 4.6 Hz, 1H), 1.69–1.57 (m, 4H), 1.53–1.38 (m, 3H), 1.37–1.29 (m, 2H), 1.35 (s, 3H), 1.26 (t, *J* = 7.1 Hz, 3H), 1.21 (s, 3H), 1.17 (s, 3H), 1.14 (m, 6H), 1.09–1.01 (m, 3H), 1.06 (s, 3H), 0.81 (s, 3H). ^13^C NMR (100 MHz, CDCl_3_) δ 199.98, 176.50, 169.62, 166.32, 138.54, 137.86, 129.02, 128.60, 128.31, 128.22, 125.31, 75.45, 61.47, 60.45, 55.59, 48.53, 45.48, 43.97, 43.41, 41.22, 40.37, 39.15, 37.85, 37.06, 32.53, 31.95, 31.25, 28.72, 28.45, 27.60, 26.60, 26.55, 23.55, 23.45, 21.56, 18.73, 18.38, 18.25, 15.80, 14.47. Exact mass calcd for C_40_H_57_NNaO_4_ [M+Na]^+^: 638.4180, Found: 638.4168.

Product **7** was a white solid in 95% yield, Mp: 149–151 °C; ^1^H NMR (400 MHz, CDCl_3_) δ 7.34–7.26 (m, 2H), 7.03–6.99 (m, 2H), 5.65 (s, 1H), 5.01 (s, 2H), 4.23–4.08 (m, 2H), 2.95–2.89 (m, 1H), 2.81–2.75 (m, 1H), 2.36 (s, 1H), 2.31–2.23 (m, 1H), 2.14–2.08 (m, 1H), 2.06–1.97 (m, 2H), 1.94–1.98 (m, 1H), 1.82 (td, *J* = 13.6, 4.6 Hz, 1H), 1.66–1.56 (m, 4H), 1.53–1.40 (m, 3H), 1.37–1.29 (m, 2H), 1.34 (s, 3H), 1.26 (t, *J* = 7.1 Hz, 3H), 1.19 (s, 3H), 1.15 (d, *J* = 6.0 Hz, 9H), 1.06–0.99 (m, 3H), 1.04 (s, 3H), 0.81 (s, 3H). ^13^C NMR (100 MHz, CDCl_3_) δ 199.99, 176.53, 169.70, 166.61, 162.41 (d, *J* = 244.9 Hz), 134.52 (d, *J* = 3.2 Hz), 130.08 (d, *J* = 8.0 Hz), 128.60, 115.12 (d, *J* = 21.1 Hz), 74.64, 61.47, 60.48, 55.59, 48.54, 45.49, 43.99, 43.43, 41.23, 40.41, 39.13, 37.86, 37.06, 32.52, 31.97, 31.26, 28.73, 28.46, 27.60, 26.62, 26.55, 23.56, 23.46, 18.74, 18.39, 18.26, 15.80, 14.48. ^19^F NMR (377 MHz, CDCl_3_) δ -115.23. Exact mass calcd for C_39_H_54_FNNaO_4_ [M+Na]^+^: 642.3929, Found: 642.3917.

Product **8** was a white solid in 99% yield, Mp: 118–120 °C; ^1^H NMR (400 MHz, CDCl_3_) δ 7.31–7.26 (m, 1H), 7.12–7.10 (m, 1H), 7.08–7.04 (m, 1H), 6.98–6.93 (m, 1H), 5.66 (s, 1H), 5.05 (s, 2H), 4.23–4.09 (m, 2H), 2.99–2.93 (m, 1H), 2.83–2.78 (m, 1H), 2.37 (s, 1H), 2.34–2.26 (m, 1H), 2.13–2.09 (m, 1H), 2.07–1.97 (m, 2H), 1.95–1.90 (m, 1H), 1.83 (td, *J* = 13.7, 4.6 Hz, 1H), 1.70–1.57 (m, 4H), 1.53–1.38 (m, 3H), 1.37–1.30 (m, 2H), 1.35 (s, 3H), 1.27 (t, *J* = 7.1 Hz, 3H), 1.21 (s, 3H), 1.17–1.13 (m, 9H), 1.10–1.00 (m, 3H), 1.05 (s, 3H), 0.81 (s, 3H). ^13^C NMR (100 MHz, CDCl_3_) δ 199.94, 176.50, 169.66, 166.76, 162.91 (d, *J* = 245.5 Hz), 141.56 (d, *J* = 7.2 Hz), 129.76 (d, *J* = 8.0 Hz), 128.59, 123.45 (d, *J* = 2.9 Hz), 114.85 (d, *J* = 21.7 Hz), 114.30 (d, *J* = 21.4 Hz), 74.52, 61.45, 60.46, 55.60, 48.54, 45.48, 43.98, 43.42, 41.22, 40.41, 39.12, 37.86, 37.06, 32.51, 31.96, 31.26, 28.72, 28.45, 27.58, 26.61, 26.55, 23.56, 23.45, 18.73, 18.38, 18.27, 15.80, 14.48. ^19^F NMR (377 MHz, CDCl_3_) δ -113.68. Exact mass calcd for C_39_H_54_FNNaO_4_ [M+Na]^+^: 642.3929, Found: 642.3911.

Product **9** was a white solid in 81% yield, Mp: 171–173 °C; ^1^H NMR (400 MHz, CDCl_3_) δ 7.40 (td, *J* = 7.5, 1.9 Hz, 1H), 7.28–7.22 (m, 1H), 7.10 (td, *J* = 7.5, 1.2 Hz, 1H), 7.05–7.00 (m, 1H), 5.66 (s, 1H), 5.13 (s, 2H), 4.23–4.08 (m, 2H), 2.98–2.92 (m, 1H), 2.82–2.76 (m, 1H), 2.37 (s, 1H), 2.33–2.25 (m, 1H), 2.13–2.09 (m, 1H), 2.07–1.97 (m, 2H), 1.94–1.89 (m, 1H), 1.83 (td, *J* = 13.7, 4.6 Hz, 1H), 1.76–1.52 (m, 4H), 1.49–1.38 (m, 3H), 1.37–1.29 (m, 2H), 1.34 (s, 3H), 1.26 (t, *J* = 7.1 Hz, 3H), 1.20 (s, 3H), 1.15 (d, *J* = 6.3 Hz, 9H), 1.09–1.00 (m, 3H), 1.05 (s, 3H), 0.81 (s, 3H). ^13^C NMR (100 MHz, CDCl_3_) δ 199.93, 176.47, 169.63, 166.66, 160.83 (d, *J* = 247.0 Hz), 130.52 (d, *J* = 4.6 Hz), 129.17 (d, *J* = 8.1 Hz), 128.56, 125.86 (d, *J* = 14.6 Hz), 123.85 (d, *J* = 3.6 Hz), 115.17 (d, *J* = 21.7 Hz), 68.75 (d, *J* = 4.0 Hz), 61.42, 60.42, 55.52, 48.50, 45.44, 43.94, 43.39, 41.19, 40.35, 39.10, 37.83, 37.02, 32.47, 31.93, 31.22, 28.69, 28.42, 27.55, 26.58, 26.51, 23.51, 23.42, 18.70, 18.34, 18.17, 15.77, 14.44. ^19^F NMR (377 MHz, CDCl_3_) δ -118.65. Exact mass calcd for C_39_H_54_FNNaO_4_ [M+Na]^+^: 642.3929, Found: 642.3911.

Product **10** was a white solid in 99% yield, Mp: 117–119 °C; ^1^H NMR (400 MHz, CDCl_3_) δ 7.31–7.26 (m, 4H), 5.66 (s, 1H), 5.01 (s, 2H), 4.23–4.08 (m, 2H), 2.96–2.90 (m, 1H), 2.82–2.76 (m, 1H), 2.36 (s, 1H), 2.32–2.23 (m, 1H), 2.14–2.09 (m, 1H), 2.07–1.97 (m, 2H), 1.94–1.89 (m, 1H), 1.83 (dt, *J* = 13.7, 6.8 Hz, 1H), 1.74–1.57 (m, 4H), 1.53–1.38 (m, 3H), 1.35–1.30 (m, 2H), 1.35 (s, 3H), 1.26 (t, *J* = 7.1 Hz, 3H), 1.20 (s, 3H), 1.14 (d, *J* = 2.8 Hz, 9H), 1.04–1.00 (m, 3H), 1.04 (s, 3H), 0.81 (s, 3H). ^13^C NMR (100 MHz, CDCl_3_) δ 199.93, 176.48, 169.68, 166.65, 137.31, 133.25, 129.56, 128.55, 128.41, 74.48, 61.42, 60.43, 55.54, 48.50, 45.45, 43.95, 43.39, 41.19, 40.38, 39.09, 37.83, 37.02, 32.47, 31.93, 31.22, 28.70, 28.42, 27.56, 26.58, 26.51, 23.53, 23.43, 18.70, 18.35, 18.22, 15.78, 14.45. HRMS (ESI): Exact mass calcd for C_39_H_54_ClNNaO_4_ [M+Na]^+^: 658.3634, Found: 658.3615.

Product **11** was a white solid in 81% yield, Mp: 115–117 °C; ^1^H NMR (400 MHz, CDCl_3_) δ 7.34 (t, *J* = 3.60 Hz, 1H), 7.28–7.20 (m, 3H), 5.66 (s, 1H), 5.03 (s, 2H), 4.23–4.08 (m, 2H), 2.98–2.92 (m, 1H), 2.83–2.77 (m, 1H), 2.37 (s, 1H), 2.34–2.26 (m, 1H), 2.14–2.07 (m, 1H), 2.03–1.98 (m, 2H), 1.95–1.90 (m, 1H), 1.83 (td, *J* = 13.6, 4.6 Hz, 1H), 1.72–1.57 (m, 4H), 1.52–1.37 (m, 3H), 1.35–1.32 (m, 2H), 1.35 (s, 3H), 1.27 (t, *J* = 7.1 Hz, 3H), 1.21 (s, 3H), 1.15 (d, *J* = 4.3 Hz, 9H), 1.05–1.00 (m, 3H), 1.05 (s, 3H), 0.81 (s, 3H). ^13^C NMR (100 MHz, CDCl_3_) δ 199.93, 176.49, 169.67, 166.86, 140.96, 134.10, 129.57, 128.56, 128.19, 127.58, 126.10, 74.45, 61.43, 60.44, 55.58, 48.51, 45.46, 43.96, 43.40, 41.20, 40.42, 39.10, 37.84, 37.04, 32.48, 31.94, 31.23, 28.71, 28.43, 27.56, 26.59, 26.52, 23.51, 23.43, 18.71, 18.36, 18.27, 15.79, 14.46. Exact mass calcd for C_39_H_54_ClNNaO_4_ [M+Na]^+^: 658.3634, Found: 658.3614.

Product **12** was a white solid in 62% yield, Mp: 189–191 °C; ^1^H NMR (400 MHz, CDCl_3_) δ 7.41 (dd, *J* = 7.4, 1.9 Hz, 1H), 7.34 (dd, *J* = 7.5, 1.7 Hz, 1H), 7.27–7.18 (m, 2H), 5.66 (s, 1H), 5.18 (s, 2H), 4.23–4.09 (m, 2H), 3.03–2.97 (m, 1H), 2.85–2.79 (m, 1H), 2.39–2.30 (m, 2H), 2.14–2.09 (m, 1H), 2.07–1.97 (m, 2H), 1.95–1.90 (m, 1H), 1.83 (td, *J* = 13.7, 4.6 Hz, 1H), 1.70–1.57 (m, 4H), 1.53–1.38 (m, 3H), 1.35–1.30 (m, 2H), 1.35 (s, 3H), 1.27 (t, *J* = 7.1 Hz, 3H), 1.21 (s, 3H), 1.16–1.14 (m, 9H), 1.09–1.00 (m, 3H), 1.06 (s, 3H), 0.81 (s, 3H). ^13^C NMR (100 MHz, CDCl_3_) δ 199.96, 176.50, 169.68, 166.89, 136.67, 132.97, 129.39, 129.26, 128.59, 128.49, 126.61, 72.35, 61.46, 60.45, 55.54, 48.53, 45.47, 43.97, 43.42, 41.23, 40.38, 39.18, 37.85, 37.06, 32.49, 31.95, 31.25, 28.72, 28.45, 27.63, 26.61, 26.54, 23.53, 23.45, 18.72, 18.38, 18.29, 15.82, 14.47. Exact mass calcd for C_39_H_54_ClNNaO_4_ [M+Na]^+^: 658.3634, Found: 658.3620.

Product **13** was a white solid in 78% yield, Mp: 158–160 °C; ^1^H NMR (400 MHz, CDCl_3_) δ 7.46–7.43 (m, 2H), 7.26–7.20 (m, 2H), 5.65 (s, 1H), 4.99 (s, 2H), 4.22–4.08 (m, 2H), 2.96–2.89 (m, 1H), 2.81–2.76 (m, 1H), 2.36 (s, 1H), 2.32–2.23 (m, 1H), 2.13–2.08 (m, 1H), 2.06–1.96 (m, 2H), 1.92–1.89 (m, 1H), 1.82 (td, *J* = 13.6, 4.6 Hz, 1H), 1.68–1.56 (m, 4H), 1.52–1.36 (m, 3H), 1.37–1.29 (m, 2H), 1.34 (s, 3H), 1.26 (t, *J* = 7.1 Hz, 3H), 1.20 (s, 3H), 1.14 (s, 9H), 1.07–0.99 (m, 3H), 1.04 (s, 3H), 0.80 (s, 3H). ^13^C NMR (100 MHz, CDCl_3_) δ 199.96, 176.50, 169.69, 166.70, 137.86, 131.39, 129.91, 128.58, 121.43, 74.52, 61.44, 60.46, 55.57, 48.53, 45.47, 43.97, 43.41, 41.21, 40.40, 39.10, 37.85, 37.04, 32.50, 31.95, 31.25, 28.72, 28.45, 27.57, 26.60, 26.54, 23.56, 23.46, 18.73, 18.37, 18.24, 15.79, 14.47. Exact mass calcd for C_39_H_54_BrNNaO_4_ [M+Na]^+^: 702.3128, Found: 602.3107.

Product **14** was a white solid in 98% yield, Mp: 111–113 °C; ^1^H NMR (400 MHz, CDCl_3_) δ 7.50 (t, *J* = 1.8 Hz, 1H), 7.39 (dt, *J* = 7.8, 1.6 Hz, 1H), 7.28–7.25 (m, 1H), 7.19 (t, *J* = 7.7 Hz, 1H), 5.66 (s, 1H), 5.02 (s, 2H), 4.23–4.09 (m, 2H), 2.98–2.91 (m, 1H), 2.83–2.77 (m, 1H), 2.37 (s, 1H), 2.35–2.25 (m, 1H), 2.14–2.08 (m, 1H), 2.04–1.97 (m, 2H), 1.94–1.89 (m, 1H), 1.83 (td, *J* = 13.6, 4.6 Hz, 1H), 1.67–1.57 (m, 4H), 1.53–1.38 (m, 3H), 1.37–1.30 (m, 2H), 1.35 (s, 3H), 1.27 (t, *J* = 7.1 Hz, 3H), 1.21 (s, 3H), 1.15–1.14 (m, 9H), 1.10–0.99 (m, 3H), 1.05 (s, 3H), 0.81 (s, 3H). ^13^C NMR (100 MHz, CDCl_3_) δ 199.97, 176.52, 169.69, 166.96, 141.26, 131.18, 130.53, 129.91, 128.60, 126.61, 122.37, 74.41, 61.46, 60.47, 55.62, 48.55, 45.49, 43.99, 43.43, 41.23, 40.46, 39.13, 37.86, 37.07, 32.52, 31.97, 31.27, 28.73, 28.46, 27.58, 26.62, 26.55, 23.52, 23.47, 18.74, 18.38, 18.30, 15.82, 14.48. Exact mass calcd for C_39_H_54_BrNNaO_4_ [M+Na]^+^: 702.3128, Found: 702.3108.

Product **15** was a white solid in 79% yield, Mp: 190–192 °C; ^1^H NMR (400 MHz, CDCl_3_) δ 7.52 (dd, *J* = 7.9, 1.2 Hz, 1H), 7.39 (dd, *J* = 7.7, 1.7 Hz, 1H), 7.29 (dd, *J* = 7.5, 1.2 Hz, 1H), 7.12 (td, *J* = 7.7, 1.8 Hz, 1H), 5.66 (s, 1H), 5.14 (s, 2H), 4.23–4.08 (m, 2H), 3.03–2.97 (m, 1H), 2.84–2.79 (m, 1H), 2.39–2.30 (m, 2H), 2.13–2.08 (m, 1H), 2.06–1.96 (m, 2H), 1.94–1.89 (m, 1H), 1.82 (td, *J* = 13.7, 4.6 Hz, 1H), 1.69–1.57 (m, 4H), 1.53–1.37 (m, 3H), 1.37–1.29 (m, 2H), 1.34 (s, 3H), 1.26 (t, *J* = 7.1 Hz, 3H), 1.21 (s, 3H), 1.15–1.14 (m, 9H), 1.08–0.99 (m, 3H), 1.05 (s, 3H), 0.81 (s, 3H). ^13^C NMR (100 MHz, CDCl_3_) δ 199.94, 176.49, 169.67, 166.95, 138.28, 132.51, 129.45, 128.75, 128.58, 127.21, 122.73, 74.59, 61.45, 60.45, 55.53, 48.52, 45.46, 43.96, 43.41, 41.22, 40.38, 39.19, 37.85, 37.05, 32.48, 31.95, 31.24, 28.72, 28.44, 27.64, 26.60, 26.53, 23.51, 23.45, 18.72, 18.38, 18.32, 15.82, 14.47. Exact mass calcd for C_39_H_54_BrNNaO_4_ [M+Na]^+^: 702.3128, Found: 702.3114.

Product **16** was a white solid in 88% yield, Mp: 153–155 °C; ^1^H NMR (400 MHz, CDCl_3_) δ 7.62–7.60 (m, 2H), 7.43–7.41 (m, 2H), 5.65 (s, 1H), 5.09 (s, 2H), 4.22–4.07 (m, 2H), 2.96–2.90 (m, 1H), 2.84–2.78 (m, 1H), 2.36–2.27 (m, 2H), 2.13–2.08 (m, 1H), 2.06–1.96 (m, 2H), 1.93–1.88 (m, 1H), 1.82 (td, J = 13.6, 4.6 Hz, 1H), 1.67–1.55 (m, 4H), 1.52–1.37 (m, 3H), 1.36–1.29 (m, 2H), 1.34 (s, 3H), 1.26 (t, J = 7.1 Hz, 3H), 1.19 (s, 3H), 1.13 (s, 6H), 1.11 (s, 3H), 1.06–0.99 (m, 3H), 1.28 (s, 3H), 0.80 (s, 3H). ^13^C NMR (100 MHz, CDCl_3_) δ 199.89, 176.48, 169.79, 167.20, 144.62, 132.14, 128.55, 128.27, 119.10, 111.15, 74.20, 61.42, 60.44, 55.50, 48.51, 45.44, 43.96, 43.41, 41.22, 40.41, 39.09, 37.83, 37.02, 32.44, 31.94, 31.23, 28.71, 28.43, 27.61, 26.59, 26.52, 23.50, 23.44, 18.70, 18.37, 18.29, 15.81, 14.46. Exact mass calcd for C_40_H_54_N_2_NaO_4_ [M+Na]^+^: 649.3976, Found: 649.3957.

Product **17** was a white solid in 74% yield, Mp: 133–135 °C; ^1^H NMR (400 MHz, CDCl_3_) δ 7.64–7.63 (m, 1H), 7.58–7.41 (m, 2H), 7.43 (t, *J* = 7.7 Hz, 1H), 5.65 (s, 1H), 5.06 (s, 2H), 4.22–4.07 (m, 2H), 2.95–2.88 (m, 1H), 2.82–2.77 (m, 1H), 2.36 (s, 1H), 2.34–2.25 (m, 1H), 2.12–2.08 (m, 1H), 2.06–1.96 (m, 2H), 1.93–1.88 (m, 1H), 1.82 (td, *J* = 13.6, 4.6 Hz, 1H), 1.69–1.56 (m, 4H), 1.52–1.37 (m, 3H), 1.34 (s, 3H), 1.36–1.28 (m, 2H), 1.25 (t, *J* = 7.1 Hz, 3H), 1.19 (s, 3H), 1.13 (s, 9H), 1.09–0.98 (m, 3H), 1.03 (s, 3H), 0.80 (s, 3H). ^13^C NMR (100 MHz, CDCl_3_) δ 199.89, 176.49, 169.73, 167.21, 140.58, 132.37, 131.61, 131.15, 129.10, 128.55, 119.06, 112.32, 73.98, 61.40, 60.44, 55.52, 48.51, 45.45, 43.95, 43.40, 41.20, 40.44, 39.06, 37.83, 37.01, 32.45, 31.94, 31.23, 28.70, 28.43, 27.59, 26.59, 26.52, 23.53, 23.44, 18.70, 18.36, 18.28, 15.78, 14.46. Exact mass calcd for C_40_H_54_N_2_NaO_4_ [M+Na]^+^: 649.3976, Found: 649.3965.

Product **18** was a white solid in 88% yield, Mp: 169–171 °C; ^1^H NMR (400 MHz, CDCl_3_) δ 7.64 (dd, *J* = 7.8, 1.2 Hz, 1H), 7.59–7.51 (m, 2H), 7.36 (td, *J* = 7.4, 1.7 Hz, 1H), 5.65 (s, 1H), 5.24 (s, 2H), 4.23–4.08 (m, 2H), 2.98–2.92 (m, 1H), 2.82–2.76 (m, 1H), 2.37 (s, 1H), 2.35–2.28 (m, 1H), 2.13–2.08 (m, 1H), 2.06–1.96 (m, 2H), 1.94–1.89 (m, 1H), 1.82 (td, *J* = 13.6, 4.6 Hz, 1H), 1.66–1.56 (m, 4H), 1.51–1.36 (m, 3H), 1.36–1.30 (m, 2H), 1.34 (s, 3H), 1.26 (t, *J* = 7.1 Hz, 3H), 1.18 (s, 3H), 1.13 (d, *J* = 1.5 Hz, 9H), 1.07–1.00 (m, 3H), 1.03 (s, 3H), 0.80 (s, 3H). ^13^C NMR (100 MHz, CDCl_3_) δ 199.96, 176.53, 169.73, 167.33, 142.66, 132.79, 132.72, 129.23, 128.58, 127.94, 117.58, 111.77, 72.72, 61.44, 60.48, 55.48, 48.54, 45.48, 43.98, 43.43, 41.23, 40.43, 39.12, 37.86, 37.04, 32.48, 31.97, 31.26, 28.73, 28.46, 27.61, 26.62, 26.55, 23.50, 23.46, 18.72, 18.38, 18.27, 15.82, 14.48. Exact mass calcd for C_40_H_54_N_2_NaO_4_ [M+Na]^+^: 649.3976, Found: 649.3961.

Product **19** was a white solid in 99% yield, Mp: 148–150 °C; ^1^H NMR (400 MHz, CDCl_3_) δ 7.58 (d, *J* = 8.1 Hz, 2H), 7.45 (d, *J* = 8.0 Hz, 2H), 5.66 (s, 1H), 5.10 (s, 2H), 4.22–4.08 (m, 2H), 2.98–2.92 (m, 1H), 2.84–2.78 (m, 1H), 2.37–2.27 (m, 2H), 2.13–2.08 (m, 1H), 2.06–1.96 (m, 2H), 1.94–1.99 (m, 1H), 1.82 (td, *J* = 13.6, 4.6 Hz, 1H), 1.70–1.56 (m, 4H), 1.52–1.38 (m, 3H), 1.37–1.30 (m, 2H), 1.37 (s, 3H), 1.26 (t, *J* = 7.1 Hz, 3H), 1.20 (s, 3H), 1.14 (d, *J* = 1.2 Hz, 9H), 1.06–1.00 (m, 3H), 1.04 (s, 3H), 0.80 (s, 3H). ^13^C NMR (100 MHz, CDCl_3_): δ 199.94, 176.50, 169.73, 166.93, 143.05, 129.62 (q, *J* = 32.3 Hz), 128.59, 128.07, 125.23 (q, *J* = 3.9 Hz), 121.68 (q, *J* = 272.0 Hz), 74.44, 61.45, 60.46, 55.57, 48.54, 45.47, 43.98, 43.42, 41.23, 40.42, 39.12, 37.86, 37.05, 32.49, 31.96, 31.25, 28.72, 28.45, 27.59, 26.61, 26.54, 23.54, 23.44, 18.73, 18.38, 18.28, 15.80, 14.47. ^19^F NMR (377 MHz, CDCl_3_) δ-62.41. Exact mass calcd for C_40_H_54_F_3_NNaO_4_ [M+Na]^+^: 692.3897, Found: 692.3880.

Product **20** was a white solid in 63% yield, Mp: 120–122 °C; ^1^H NMR (400 MHz, CDCl_3_) δ 7.61–7.55 (m, 4H), 7.45–7.01 (m, 4H), 7.35–7.31 (m, 1H), 5.66 (s, 1H), 5.11 (s, 2H), 4.23–4.09 (m, 2H), 3.02–2.96 (m, 1H), 2.83–2.77 (m, 1H), 2.36 (s, 1H), 2.34–2.26 (m, 1H), 2.14–2.08 (m, 1H), 2.06–1.97 (m, 2H), 1.94–1.98 (m, 1H), 1.82 (td, *J* = 13.6, 4.6 Hz, 1H), 1.70–1.58 (m, 4H), 1.53–1.37 (m, 3H), 1.35–1.30 (m, 2H),1.32 (s, 3H) 1.27 (t, *J* = 7.1 Hz, 3H), 1.22 (s, 3H), 1.19 (s, 3H), 1.15 (s, 6H), 1.07–0.99 (m, 3H), 1.07 (s, 3H), 0.81 (s, 3H). ^13^C NMR (100 MHz, CDCl_3_) δ 199.97, 176.49, 169.62, 166.61, 141.12, 140.48, 137.75, 128.85, 128.73, 128.57, 127.31, 127.21, 127.06, 75.09, 61.45, 60.45, 55.63, 48.51, 45.47, 43.96, 43.39, 41.20, 40.42, 39.14, 37.84, 37.06, 32.51, 31.94, 31.24, 28.71, 28.45, 27.56, 26.59, 26.53, 23.57, 23.41, 18.73, 18.36, 18.28, 15.80, 14.47. Exact mass calcd for C_45_H_59_NNaO_4_ [M+Na]^+^: 700.4336, Found: 700.4331.

Product **21** was a white solid in 99% yield, Mp: 127–129 °C; ^1^H NMR (400 MHz, CDCl_3_) δ 7.84–7.80 (m, 4H), 7.52–7.48 (m, 1H), 7.47–7.43 (m, 2H), 5.66 (s, 1H), 5.23 (s, 2H), 4.23–4.09 (m, 2H), 3.04–2.98 (m, 1H), 2.85–2.79 (m, 1H), 2.36–2.28 (m, 2H), 2.14–2.09 (m, 1H), 2.06–1.97 (m, 2H), 1.96–1.90 (m, 1H), 1.82 (td, *J* = 13.7, 4.7 Hz, 1H), 1.74–1.57 (m, 4H), 1.53–1.37 (m, 3H), 1.38–1.30 (m, 2H), 1.33 (s, 3H), 1.27 (t, *J* = 7.1 Hz, 3H), 1.22 (s, 3H), 1.18 (s, 3H), 1.14 (d, *J* = 3.4 Hz, 6H), 1.11–0.98 (m, 3H), 1.07 (s, 3H), 0.81 (s, 3H). ^13^C NMR (100 MHz, CDCl_3_) δ 199.94, 176.46, 169.62, 166.45, 136.28, 133.37, 132.98, 128.54, 128.03, 127.90, 127.74, 126.87, 126.38, 125.99, 125.79, 75.43, 61.42, 60.41, 55.55, 48.49, 45.43, 43.93, 43.36, 41.18, 40.37, 39.13, 37.82, 37.03, 32.46, 31.91, 31.21, 28.68, 28.41, 27.56, 26.55, 26.50, 23.54, 23.41, 18.68, 18.33, 18.24, 15.78, 14.44. Exact mass calcd for C_43_H_57_NNaO_4_ [M+Na]^+^: 674.4180, Found: 674.4157.

Product **22** was a white solid in 99% yield, Mp: 178–150 °C; ^1^H NMR (400 MHz, CDCl_3_) δ 8.15–8.13 (m, 1H), 7.87–7.85 (m, 1H), 7.82–7.79 (m, 1H), 7.54–7.49 (m, 3H), 7.47–7.42 (m, 1H), 5.65 (s, 1H), 5.53 (d, *J* = 2.0 Hz, 2H), 4.23–4.08 (m, 2H), 2.98–2.91 (m, 1H), 2.76–2.70 (m, 1H), 2.35 (s, 1H), 2.31–2.23 (m, 1H), 2.13–2.08 (m, 1H), 2.07–1.97 (m, 2H), 1.94–1.89 (m, 1H), 1.82 (td, *J* = 13.7, 4.6 Hz, 1H), 1.68–1.57 (m, 4H), 1.52–1.37 (m, 3H), 1.34–1.29 (m, 2H), 1.34 (s, 3H), 1.26 (t, *J* = 7.1 Hz, 3H), 1.19 (d, *J* = 2.4 Hz, 6H), 1.14 (d, *J* = 2.8 Hz, 6H), 1.07–0.96 (m, 3H), 1.07 (s, 3H), 0.81 (s, 3H). ^13^C NMR (100 MHz, CDCl_3_) δ 199.94, 176.49, 169.60, 166.62, 134.19, 133.77, 132.00, 128.58, 128.55, 128.51, 126.79, 126.12, 125.75, 125.38, 124.42, 73.84, 61.45, 60.45, 55.56, 48.53, 45.46, 43.97, 43.40, 41.21, 40.39, 39.19, 37.85, 37.05, 32.51, 31.95, 31.25, 28.72, 28.45, 27.61, 26.59, 26.54, 23.56, 23.45, 18.72, 18.37, 18.22, 15.80, 14.47. Exact mass calcd for C_43_H_57_NNaO_4_ [M+Na]^+^: 674.4180, Found: 674.4167.

Product **23** was a white solid in 98% yield, Mp: 139–141 °C; ^1^H NMR (400 MHz, CDCl_3_) δ 6.98 (s, 2H), 6.91 (s, 1H), 5.65 (s, 1H), 4.99 (s, 2H), 4.21–4.10 (m, 2H), 2.99–2.93 (m, 1H), 2.81–2.75 (m, 1H), 2.37 (s, 1H),2.33–2.25 (m, 1H), 2.31 (s, 6H), 2.13–2.08 (m, 1H), 2.07–1.97 (m, 2H), 1.94–1.89 (m, 1H), 1.83 (td, *J* = 13.6, 4.6 Hz, 1H), 1.71–1.57 (m, 4H), 1.53–1.38 (m, 3H), 1.37–1.29 (m, 2H), 1.35 (s, 3H), 1.26 (t, *J* = 7.1 Hz, 3H), 1.21 (s, 3H), 1.18 (s, 3H), 1.14 (d, *J* = 1.5 Hz, 6H), 1.09–1.00 (m, 3H), 1.06 (s, 3H), 0.81 (s, 3H). ^13^C NMR (100 MHz, CDCl_3_) δ 199.98, 176.51, 169.62, 166.25, 138.39, 137.79, 129.23, 128.60, 126.13, 75.50, 61.49, 60.45, 55.59, 48.54, 45.48, 43.97, 43.41, 41.21, 40.36, 39.17, 37.85, 37.06, 32.53, 31.96, 31.25, 28.72, 28.45, 27.62, 26.61, 26.54, 23.53, 23.44, 21.42, 18.73, 18.38, 18.25, 15.81, 14.47. Exact mass calcd for C_41_H_59_NNaO_4_ [M+Na]^+^: 652.4336, Found: 652.4318.

Product **24** was a white solid in 82% yield, Mp: 137–139 °C; ^1^H NMR (400 MHz, CDCl_3_) δ 6.87–6.82 (m, 2H), 6.69 (tt, *J* = 9.0, 2.4 Hz, 1H), 5.66 (s, 1H), 5.02 (s, 2H), 4.23–4.08 (m, 2H), 2.98–2.92 (m, 1H), 2.85–2.79 (m, 1H), 2.37 (s, 1H), 2.35–2.26 (m, 1H), 2.13–2.08 (m, 1H), 2.06–1.96 (m, 2H), 1.94–1.89 (m, 1H), 1.83 (td, *J* = 13.7, 4.6 Hz, 1H), 1.69–1.56 (m, 4H), 1.53–1.37 (m, 3H), 1.37–1.29 (m, 2H), 1.35 (s, 3H), 1.26 (t, *J* = 7.1 Hz, 3H), 1.21 (s, 3H), 1.14 (d, *J* = 2.4 Hz, 9H), 1.07–1.00 (m, 3H), 1.05 (s, 3H) 0.81 (s, 3H). ^13^C NMR (100 MHz, CDCl_3_): δ 198.93, 175.52, 168.71, 166.11, 162.09 (d, *J* = 248.9 Hz),161.97 (d, *J* = 247.8 Hz), 142.22 (t, *J* = 8.7 Hz), 127.59, 109.37 (d, *J* = 24.9 Hz), 109.37 (d, *J* = 11.7 Hz), 101.68 (t, *J* = 25.4 Hz), 72.98, 60.44, 59.47, 54.60, 47.54, 44.48, 42.98, 42.43, 40.23, 39.44, 38.10, 36.86, 36.06, 31.50, 30.96, 30.26, 27.73, 27.45, 26.57, 25.62, 25.55, 22.55, 22.45, 17.74, 17.38, 17.30, 14.79, 13.48. ^19^F NMR (377 MHz, CDCl_3_) δ -110.38. Exact mass calcd for C_39_H_53_F_2_NNaO_4_ [M+Na]^+^: 660.3835, Found: 660.3819.

Product **25** was a white solid in 83% yield, Mp: 131–133 °C; ^1^H NMR (400 MHz, CDCl_3_) δ 7.19–7.12 (m, 1H), 7.10–7.02 (m, 2H), 5.64 (s, 1H), 4.97 (s, 2H), 4.22–4.07 (m, 2H), 2.94–2.88 (m, 1H), 2.82–2.76 (m, 1H), 2.36 (s, 1H), 2.32–2.23 (m, 1H), 2.12–2.07 (m, 1H), 2.06–1.96 (m, 2H), 1.93–1.88 (m, 1H), 1.82 (td, *J* = 13.6, 4.5 Hz, 1H), 1.76–1.56 (m, 4H), 1.52–1.37 (m, 3H), 1.36–1.28 (m, 2H), 1.34 (s, 3H), 1.25 (t, *J* = 7.1, 1.1 Hz, 3H), 1.19 (s, 3H), 1.13 (s, 9H), 1.07–0.99 (m, 3H), 1.04 (s, 3H), 0.80 (s, 3H). ^13^C NMR (100 MHz, CDCl_3_): δ 199.89, 176.47, 169.70, 166.85, 150.21 (dd, *J* = 248.5, 12.9 Hz), 149.80 (dd, *J* = 248.2, 12.5 Hz), 135.98 (dd, *J* = 5.3, 4.2 Hz), 128.54, 124.00 (dd, *J* = 6.5, 3.6 Hz), 117.01 (dd, *J* = 17.3, 12.3 Hz), 74.00, 61.41, 60.42, 55.54, 48.50, 45.43, 43.94, 43.38, 41.19, 40.39, 39.06, 37.82, 37.01, 32.45, 31.92, 31.21, 28.69, 28.41, 27.56, 26.57, 26.50, 23.52, 23.40, 18.69, 18.35, 18.23, 15.76, 14.44. ^19^F NMR (377 MHz, CDCl_3_): δ-138.34 (d, *J* = 20.8 Hz, 1F), -139.99 (d, *J* = 20.8 Hz, 1F). Exact mass calcd for C_39_H_53_F_2_NNaO_4_ [M+Na]^+^: 660.3835, Found: 660.3833.

Product **26** is a white solid in 69% yield, Mp: 162–164 °C; ^1^H NMR (400 MHz, CDCl_3_) δ 7.43 (d, *J* = 2.0 Hz, 1H), 7.39 (d, *J* = 8.2 Hz, 1H), 7.17 (dd, *J* = 8.2, 2.0 Hz, 1H), 5.65 (s, 1H), 4.99 (s, 2H), 4.22–4.08 (m, 2H), 2.95–2.89 (m, 1H), 2.83–2.77 (m, 1H), 2.36 (s, 1H), 2.33–2.24 (m, 1H), 2.13–2.08 (m, 1H), 2.05–1.96 (m, 2H), 1.94–1.89 (m, 1H), 1.82 (td, *J* = 13.6, 4.6 Hz, 1H), 1.66–1.56 (m, 4H), 1.52–1.36 (m, 3H), 1.34–1.28 (m, 2H), 1.34 (s, 3H), 1.26 (t, *J* = 7.1 Hz, 3H), 1.20 (s, 3H), 1.14 (s, 9H), 1.05–0.99 (m, 3H), 1.04 (s, 3H), 0.80 (s, 3H). ^13^C NMR (100 MHz, CDCl_3_) δ 199.95, 176.52, 169.74, 167.11, 139.29, 132.27, 131.36, 130.28, 130.08, 128.57, 127.39, 73.79, 61.44, 60.47, 55.60, 48.53, 45.48, 43.98, 43.42, 41.22, 40.47, 39.11, 37.85, 37.05, 32.49, 31.96, 31.25, 28.72, 28.45, 27.57, 26.61, 26.54, 23.52, 23.46, 18.73, 18.37, 18.29, 15.81, 14.47. HRMS (ESI): Exact mass calcd for C_39_H_53_Cl_2_NNaO_4_ [M+Na]^+^: 692.3244, Found: 692.3221.

Product **27** was a white solid in 95% yield, Mp: 142–144 °C; ^1^H NMR (400 MHz, CDCl_3_) δ 7.55 (t, *J* = 1.8 Hz, 1H), 7.41 (d, *J* = 1.8 Hz, 2H), 5.66 (s, 1H), 4.98 (s, 2H), 4.23–4.08 (m, 2H), 2.96–2.90 (m, 1H), 2.84–2.79 (m, 1H), 2.38 (s, 1H), 2.34–2.26 (m, 1H), 2.13–2.08 (m, 1H), 2.07–1.97 (m, 2H), 1.95–1.89 (m, 1H), 1.83 (td, *J* = 13.7, 4.6 Hz, 1H), 1.67–1.56 (m, 4H), 1.52–1.38 (m, 3H), 1.37–1.30 (m, 2H), 1.35 (s, 3H), 1.26 (t, *J* = 7.1 Hz, 3H), 1.21 (s, 3H), 1.15–1.12 (m, 9H), 1.08–0.99 (m, 3H), 1.05 (s, 3H), 0.81 (s, 3H). ^13^C NMR (100 MHz, CDCl_3_) δ 199.94, 176.53, 169.73, 167.41, 143.09, 132.96, 129.73, 128.60, 122.79, 73.60, 61.46, 60.48, 55.64, 48.55, 45.49, 43.99, 43.44, 41.23, 40.54, 39.13, 37.86, 37.08, 32.50, 31.97, 31.27, 28.73, 28.47, 27.58, 26.63, 26.56, 23.49, 23.48, 18.74, 18.38, 18.35, 15.84, 14.49. Exact mass calcd for C_39_H_53_Br_2_NNaO_4_ [M+Na]^+^: 780.2234, Found: 780.2198.

Product **28** was a white solid in 84% yield, Mp: 147–149 °C; ^1^H NMR (400 MHz, CDCl_3_) δ 7.28–7.25 (m, 1H), 7.23–7.22 m, 1H), 7.12–7.10 (m, 1H), 5.65 (s, 1H), 5.05 (s, 2H), 4.23–4.08 (m, 2H), 2.97–2.91 (m, 1H), 2.81–2.75 (m, 1H), 2.37 (s, 1H), 2.31–2.22 (m, 1H), 2.13–2.08 (m, 1H), 2.07–1.97 (m, 2H), 1.94–1.89 (m, 1H), 1.83 (td, *J* = 13.6, 4.8 Hz, 1H), 1.70–1.57 (m, 4H), 1.54–1.38 (m, 3H), 1.35–1,30 (m, 2H), 1.35 (s, 3H), 1.26 (t, *J* = 7.1 Hz, 3H), 1.21 (s, 3H), 1.18 (s, 3H), 1.14 (s, 6H), 1.06–1.01 (m, 3H), 1.06 (s, 3H), 0.81 (s, 3H). ^13^C NMR (100 MHz, CDCl_3_) δ 199.95, 176.48, 169.63, 166.36, 139.73, 128.57, 127.96, 125.50, 123.14, 70.48, 61.43, 60.44, 55.57, 48.51, 45.46, 43.95, 43.40, 41.19, 40.36, 39.10, 37.83, 37.03, 32.50, 31.94, 31.23, 28.70, 28.43, 27.58, 26.59, 26.52, 23.57, 23.44, 18.71, 18.35, 18.18, 15.77, 14.45. HRMS (ESI): Exact mass calcd for C_37_H_53_NNaO_3_S [M+Na]^+^: 630.3588, Found: 630.3571.

Product **29** is a white solid in 53% yield, Mp: 131–133 °C; ^1^H NMR (400 MHz, CDCl_3_) δ 7.44 (t, *J* = 2 Hz, 1H), 5.65 (s, 1H), 5.09 (d, *J* = 0.8 Hz, 2H), 4.23–4.08 (m, 2H), 2.89–2.77 (m, 2H), 2.36 (s, 1H), 2.28–2.20 (m, 2H), 2.13–2.08 (m, 1H), 2.07–1.97 (m, 2H), 1.94–1.89 (m, 1H), 1.83 (td, *J* = 13.7, 4.6 Hz, 1H), 1.60–1.58L (m, 7H), 1.51–1.38 (m, 4H), 1.35 (s, 3H), 1.26 (t, *J* = 7.1 Hz, 3H), 1.21 (d, *J* = 2.6 Hz, 6H), 1.14 (d, *J* = 3.9 Hz, 6H), 1.08 (s, 3H), 0.81 (s, 3H). ^13^C NMR (100 MHz, CDCl_3_) δ 199.95, 176.54, 169.81, 167.84, 152.82, 139.76, 137.96, 128.58, 67.14, 61.42, 60.49, 55.60, 48.55, 45.49, 44.00, 43.45, 41.23, 40.66, 39.02, 37.86, 37.05, 32.51, 31.97, 31.27, 28.74, 28.46, 27.61, 26.63, 26.55, 23.59, 23.48, 18.75, 18.37, 18.33, 15.81, 14.48. HRMS (ESI): Exact mass calcd for C_36_H_51_ClN_2_NaO_4_S [M+Na]^+^: 665.3150, Found: 665.3135.

Product **30** was a white solid in 85% yield, Mp: 174–176 °C; ^1^H NMR (400 MHz, CDCl_3_) δ 6.01 (s, 1H), 5.64 (s, 1H), 4.96 (s, 2H), 4.20–4.09 (m, 2H), 3.82 (s, 3H), 2.90–2.84 (m, 1H), 2.80–2.74 (m, 1H), 2.35 (s, 1H), 2.22 (s, 3H), 2.13–2.11 (m, 1H), 2.09–1.96 (m, 2H), 1.93–1.88 (m, 1H), 1.86–1.76 (m, 2H), 1.68–1.56 (m, 3H), 1.49–1.35 (m, 4H), 1.35–1.29 (m, 2H), 1.35 (s, 3H), 1.25 (t, *J* = 7.1 Hz, 3H), 1.19 (s, 3H), 1.13 (d, *J* = 3.3 Hz, 9H), 1.04–0.97 (m, 3H), 1.04 (s, 3H), 0.80 (s, 3H).^13^C NMR (100 MHz, CDCl_3_) δ 199.91, 176.46, 169.71, 167.85, 166.64, 160.23, 128.50, 111.84, 63.64, 61.40, 60.41, 55.54, 48.48, 45.43, 43.93, 43.39, 41.17, 40.36, 39.12, 37.81, 37.03, 32.45, 31.91, 31.20, 28.68, 28.40, 27.38, 26.56, 26.49, 23.48, 23.44, 18.69, 18.30, 17.95, 15.76, 14.43, 11.25, 10.28. Exact mass calcd for C_38_H_57_N_3_NaO_4_ [M+Na]^+^: 642.4241, Found: 642.4234.

Product **31** was a white solid in 90% yield, Mp: 185–187 °C; ^1^H NMR (400 MHz, CDCl_3_) δ 5.63 (s, 1H), 4.76 (s, 2H), 4.21–4.06 (m, 2H), 2.86–2.80 (m, 1H), 2.78–2.73 (m, 1H), 2.38 (s, 3H), 2.34 (s, 1H), 2.26 (s, 3H), 2.22–1.13 (m, 1H), 2.11–2.06 (m, 1H), 2.02–1.95 (m, 2H), 1.92–1.87 (m, 1H), 1.83–1.79 (m, 2H), 1.68–1.54 (m, 3H), 1.50–1.36 (m, 3H), 1.33–1.28 (m, 2H), 1.33 (s, 3H), 1.24 (t, *J* = 7.1 Hz, 3H), 1.17 (s, 3H), 1.11 (d, *J* = 5.9 Hz, 9H), 1.02–0.94 (m, 3H), 1.01 (s, 3H), 0.79 (s, 3H).^13^C NMR (100 MHz, CDCl_3_) δ 199.91, 176.46, 169.71, 167.85, 166.64, 160.23, 128.50, 111.84, 63.64, 61.40, 60.41, 55.54, 48.48, 45.43, 43.93, 43.39, 41.17, 40.36, 39.12, 37.81, 37.03, 32.45, 31.91, 31.20, 28.68, 28.40, 27.38, 26.56, 26.49, 23.48, 23.44, 18.69, 18.30, 17.95, 15.76, 14.43, 11.25, 10.28. Exact mass calcd for C_38_H_56_N_2_NaO_5_ [M+Na]^+^: 643.4081, Found: 643.4071.

Product **32** was a white solid in 54% yield, Mp: 159–161 °C; ^1^H NMR (400 MHz, CDCl_3_) δ 7.14 (d, *J* = 3.5 Hz, 1H), 6.42 (d, *J* = 3.5 Hz, 1H), 5.65 (s, 1H), 5.02 (s, 2H), 4.21–4.10 (m, 2H), 3.89 (s, 3H), 2.93–2.87 (m, 1H), 2.81–2.75 (m, 1H), 2.36 (s, 1H), 2.30–2.21 (m, 1H), 2.13–2.08 (m, 1H), 2.06–1.97 (m, 2H), 1.94–1.89 (m, 1H), 1.83 (td, *J* = 13.6, 4.6 Hz, 1H), 1.60–1.56 (m, 6H), 1.47–1.37 (m, 3H), 1.34 (s, 3H), 1.26 (t, *J* = 7.1 Hz, 3H), 1.20 (s, 3H), 1.14 (d, *J* = 3.2 Hz, 9H), 1.07–0.99 (m, 3H), 1.04 (s, 3H), 0.80 (s, 3H). ^13^C NMR (100 MHz, CDCl_3_) δ 199.95, 176.53, 169.69, 167.18, 159.37, 157.02, 144.02, 128.60, 119.08, 111.02, 67.33, 61.43, 60.49, 55.55, 52.03, 48.56, 45.49, 43.99, 43.43, 41.24, 40.44, 39.05, 37.87, 37.04, 32.52, 31.98, 31.27, 28.74, 28.47, 27.56, 26.63, 26.56, 23.59, 23.47, 18.75, 18.38, 18.22, 15.79, 14.49. Exact mass calcd for C_39_H_55_NNaO_7_ [M+Na]^+^: 672.3871, Found: 672.3844.

Product **33** was a white solid in 60% yield, Mp: 134–136 °C; ^1^H NMR (400 MHz, CDCl_3_) δ 7.12 (d, *J* = 3.5 Hz, 1H), 6.40 (d, *J* = 3.4 Hz, 1H), 5.64 (s, 1H), 5.02 (s, 2H), 4.35 (q, *J* = 7.1 Hz, 2H), 4.22–4.08 (m, 2H), 2.93–2,87 (m, 1H), 2.80–2,74 (m, 1H), 2.36 (s, 1H), 2.29–2.20 (m, 1H), 2.12–2.07 (m, 1H), 2.06–1.96 (m, 2H), 1.94–1.88 (m, 1H), 1.82 (td, *J* = 13.6, 4.6 Hz, 1H), 1.65–1.56 (m, 3H), 1.49–1.39 (m, 2H), 1.38–1.34 (m, 7H), 1.28–1.24 (m, 6H), 1.19 (s, 3H), 1.14 (d, *J* = 4.7 Hz, 9H), 1.07–0.99 (m, 3H), 1.04 (s, 3H), 0.80 (s, 3H).^13^C NMR (100 MHz, CDCl_3_) δ 199.94, 176.51, 169.67, 167.11, 158.99, 156.86, 144.31, 128.58, 118.81, 110.95, 61.41, 61.02, 60.46, 55.54, 48.54, 45.47, 43.97, 43.42, 41.22, 40.43, 39.03, 37.85, 37.02, 32.50, 31.96, 31.25, 29.83, 28.72, 28.45, 27.54, 26.61, 26.54, 23.58, 23.45, 18.73, 18.36, 18.20, 15.77, 14.50, 14.47. Exact mass calcd for C_40_H_57_NNaO_7_ [M+Na]^+^: 686.4027, Found: 686.4010.

Product **34** is a white solid in 48% yield, Mp: 164–166 °C; ^1^H NMR (400 MHz, CDCl_3_) δ 5.65 (s, 1H), 4.23–4.08 (m, 2H), 3.99–3.95 (m, 2H), 3.89–3.85 (m, 2H), 3.43–3.36 (m, 2H), 2.93–2.87 (m, 1H), 2.82–2.76 (m, 1H), 2.38 (s, 1H), 2.30–2.22 (m, 1H), 2.13–2.08 (m, 1H), 2.06–1.95 (m, 2H), 1.94–1.89 (m, 1H), 1.83 (td, *J* = 13.6, 4.7 Hz, 1H), 1.63–1.60 (m, 8H), 1.44–1.36 (m, 3H), 1.35–1.30 (m, 3H), 1.35 (s, 3H), 1.26 (t, *J* = 7.1 Hz, 3H), 1.22 (s, 3H), 1.16 (s, 3H), 1.14 (d, *J* = 3.7 Hz, 6H), 1.10–1.00 (m, 3H), 1.06 (s, 3H), 0.81 (s, 3H). ^13^C NMR (100 MHz, CDCl_3_) δ 200.04, 176.55, 169.73, 165.71, 128.60, 78.02, 67.92, 61.49, 60.49, 55.61, 48.55, 45.51, 44.00, 43.44, 41.24, 40.26, 39.20, 37.87, 37.10, 35.19, 32.53, 31.97, 31.27, 30.02, 28.74, 28.47, 27.65, 26.63, 26.56, 23.60, 23.47, 18.75, 18.42, 18.01, 15.82, 14.48. HRMS (ESI): Exact mass calcd for C_38_H_59_NNaO_5_ [M+Na]^+^: 632.4285, Found: 632.4273.

Product **35** was a white solid in 89% yield, Mp: 185–187 °C; ^1^H NMR (400 MHz, CDCl_3_) δ 5.65 (s, 1H), 4.63–4.60 (m, 2H), 4.32 (dd, *J* = 5.8, 1.2 Hz, 2H), 4.22–4.08 (m, 4H), 2.93–2.86 (m, 1H), 2.82–2.76 (m, 1H), 2.37 (s, 1H), 2.30–2.22 (m, 1H), 2.12–2.08 (m, 1H), 2.06–1.96 (m, 2H), 1.93–1.88 (m, 1H), 1.82 (td, *J* = 13.6, 4.6 Hz, 1H), 1.71–1.56 (m, 4H), 1.52–1.37 (m, 3H), 1.34 (s, 3H), 1.33–1.30 (m, 2H), 1.33 (s, 3H), 1.25 (t, *J* = 7.1 Hz, 3H), 1.21 (s, 3H), 1.13 (d, *J* = 3.0 Hz, 9H), 1.05–0.99 (m, 3H), 1.04 (s, 3H), 0.80 (s, 3H). ^13^C NMR (100 MHz, CDCl_3_) δ 199.93, 176.50, 169.69, 166.20, 128.58, 80.74, 80.66, 77.83, 61.43, 60.45, 55.55, 48.52, 45.47, 43.97, 43.42, 41.22, 40.37, 40.23, 39.12, 37.84, 37.07, 32.49, 31.95, 31.24, 28.72, 28.44, 27.66, 26.61, 26.54, 23.54, 23.45, 21.65, 18.72, 18.40, 17.97, 15.79, 14.46. Exact mass calcd for C_37_H_57_NNaO_5_ [M+Na]^+^: 618.4129, Found: 618.4118.

### 3.4. Inhibitory Effects on HIV-1 Protease

The enzyme activity of HIV-1 protease was assessed using a FRET assay, following the guidelines provided by the manufacturer. Briefly, HIV-1 protease activity was analyzed using 384-well microplates with a well volume of 120 µL. Recombinant HIV-1 protease (8 µL) was mixed with 2 µL of test compound (0.01~1.0 mg/mL). The reactions were initiated by adding 10 µL of HIV protease substrate (diluted 50 times with assay buffer). Fluorescence measurement was conducted using an EX/Em = 490/520 nm filter module on a Synergy II microplate reader. The inhibition rate was subsequently calculated using Microsoft Office Excel 2019 and SPSS software (version 19), followed by statistical analysis.

### 3.5. Cell Culture

Human cancer cell lines K562 and Hela and mouse cancer cell lines CT26 Cells were cultured aseptically in RPMI-1640 media with 10% (*v*/*v*) FBS and 1% (*v*/*v*) penicillin-streptomycin at 37 °C with 5% CO_2_. All cells used in the experiment were in the logarithmic growth stage.

### 3.6. MTT Assay

A total of 5 × 10^3^ logarithmically growing cells were inoculated into each well of a 96-well plate and incubated for 24 h at 37 °C with 5% CO_2_. Subsequently, various concentrations of compounds were added to each well and incubated for an additional 48 h. Next, 10 µL of MTT reagent (5 mg/mL) was added to each well to react with the mitochondria of viable cells for approximately 4 h. Finally, the medium was discarded, and the blue crystals were completely dissolved in 150 µL of DMSO. Absorbance at a wavelength of 490 nm was measured using a microplate reader. The concentration of compounds that resulted in a 50% inhibition of cell growth (IC_50_) was calculated using IBM SPSS Statistics (version 19). The IC_50_ value for each compound was determined in a minimum of three independent experiments.

### 3.7. Docking Studies

Molecular docking was conducted using the ligand docking module within the Schrödinger Suite (Schrödinger, LLC, New York City, NY, USA). The crystal structures of HIV-1 protease (PDB ID: 1QBS) were selected for docking. Prior to docking, protein preparation was performed using the protein preparation wizard, which involved assigning bond orders, adding hydrogen atoms, and eliminating water molecules. Subsequently, the protein structure was energy-minimized through restrained minimization employing the OPLS3 force field. The docking grid was generated at the orthosteric site, which was identified by selecting the original co-crystalized ligand using the receptor grid generation module. For the small-molecule compounds, 3D structures were generated and subjected to energy minimization using the LigPrep module. The ligand docking module was employed to dock the compound molecules onto the orthosteric site of HIV-1 protease, with the choice of Glide XP for docking precision.

## 4. Conclusions

In this study, a total of 32 derivatives of glycyrrhetinic acid were designed and synthesized. All compounds were characterized by ^1^H NMR, ^13^C NMR, and HRMS. Towards HIV-1 protease, compounds **28** and **32** exhibited potent inhibitory activities, with corresponding inhibition rates of 76% and 70.5%, respectively, at 1 mg/mL concentration. Further molecular docking implies that a 3-substituted polar group would provide GA derivatives with better inhibition of HIV-1 protease. We also evaluated the compounds for their inhibitory effects on the cell viability of the human cancer cell lines K562 and Hela and the mouse cancer cell line CT26 using the MTT assay. The results demonstrated that the introduction of the thiazole heterocycle, as seen in compound **29**, significantly improved its anti-proliferative activity against K562 cells with an IC_50_ value of 8.86 ± 0.93 µM. The introduction of an electron-withdrawing substituent on the benzene ring enhanced anti-proliferative activity against Hela and CT26 cells. Compound **13** exhibited the highest inhibitory activity against Hela cells with the IC_50_ value of 9.89 ± 0.86 µM, whereas compound **7** demonstrated the most potent inhibition against CT26 cells with the IC_50_ value of 4.54 ± 0.37 µM. In conclusion, our current study shows that further structural modification of glycyrrhetinic acid is a very promising strategy for developing novel anti-HIV and anticancer therapeutics.

## Figures and Tables

**Table 1:** In vitro inhibitory activity against HIV-1 protease.

Compound	Inhibition Rate (%) 1 mg/mL	Compound	Inhibition Rate(%) 1 mg/mL
**4**	11.80 ± 3.12	**21**	29.00 ± 7.75
**5**	1.00 ± 0.60	**22**	26.60 ± 1.53
**6**	4.51 ± 0.58	**23**	2.25 ± 0.87
**7**	53.60 ± 1.87	**24**	39.30 ± 2.21
**8**	12.50 ± 3.28	**25**	38.50 ± 1.01
**9**	21.10 ± 2.85	**26**	7.38 ± 3.48
**10**	11.80 ± 3.74	**27**	33.60 ± 2.25
**11**	12.40 ± 4.54	**28**	76.00 ± 1.53
**12**	21.50 ± 1.45	**29**	20.90 ± 1.07
**13**	26.80 ± 2.15	**30**	36.20 ± 2.94
**14**	25.50 ± 2.48	**31**	17.10 ± 2.88
**15**	34.30 ± 1.83	**32**	70.50 ± 2.46
**16**	23.40 ± 3.34	**33**	34.60 ± 13.0
**17**	28.10 ± 4.55	**34**	46.80 ± 4.54
**18**	29.20 ± 2.32	**35**	38.90 ± 1.34
**19**	26.20 ± 7.48	**Pepstatin A**	99.84 ± 1.06
**20**	20.30 ± 2.28		

**Table 2:** Docking of compounds **28** and **32** into the active site of HIV-1 protease (PDB ID: 1QBS).

Compound	Docking Score	Glide Emodel (kcal/moL)
**28**	−7.236	−53.102
**32**	−6.834	−64.549

**Table 3:** In vitro effect of compounds on viability of cancer cells.

Compound	IC_50_ (µM) ^a^			Compound	IC_50_ (µM)		
	K562	Hela	CT26		K562	Hela	CT26
**4**	12.25 ± 1.18	24.65 ± 1.27	76.65 ± 3.56	**21**	>100	81.28 ± 4.01	71.43 ± 3.81
**5**	53.89 ± 2.79	28.03 ± 1.86	25.62 ± 1.46	**22**	>100	>100	>100
**6**	78.10 ± 3.22	42.19 ± 2.54	22.45 ±1.86	**23**	66.16 ± 4.14	42.24 ± 2.14	33.42 ± 1.66
**7**	13.26 ± 0.89	29.49 ± 2.01	4.54 ± 0.37	**24**	19.65 ± 1.45	12.65 ± 0.85	10.38 ± 0.93
**8**	15.32 ± 1.07	41.04 ± 2.62	8.69 ± 0.72	**25**	22.42 ± 1.36	23.73 ± 1.52	39.52 ± 2.92
**9**	20.67 ± 1.36	34.96 ± 2.26	21.72 ± 2.02	**26**	30.83 ± 1.91	43.65 ± 2.93	54.85 ± 3.21
**10**	36.85 ± 2.34	56.12 ± 3.34	14.33 ± 1.27	**27**	24.34 ± 1.27	18.81 ± 1.03	74.86 ± 4.74
**11**	66.50 ± 3.52	23.41 ± 1.32	96.28 ± 4.32	**28**	14.38 ± 1.23	24.85 ± 1.61	24.51 ± 1.77
**12**	73.69 ± 3.25	46.40 ± 3.52	31.36 ± 1.83	**29**	8.86 ± 0.93	15.36 ± 0.87	12.29 ± 1.09
**13**	25.01 ± 1.27	9.89 ± 0.86	55.87 ± 2.37	**30**	24.51 ± 1.77	14.51 ± 0.74	20.21 ± 1.82
**14**	86.53 ± 4.32	>100	>100	**31**	22.49 ± 1.39	12.27 ± 0.84	7.39 ± 0.93
**15**	53.31 ± 3.24	69.71 ± 4.21	45.14 ± 3.03	**32**	18.11 ± 0.82	20.53 ± 1.18	31.28 ± 2.02
**16**	>100	>100	>100	**33**	27.39 ± 2.03	67.36 ± 3.93	25.26 ± 1.37
**17**	45.59 ± 2.47	>100	>100	**34**	21.28 ± 1.82	30.31 ± 1.62	37.39 ± 1.93
**18**	55.24 ± 3.19	23.34 ± 1.76	76.52 ± 4.25	**35**	55.46 ± 3.38	38.35 ± 2.07	49.28 ± 2.62
**19**	16.14 ± 1.02	>100	>100	**Cisplatin** **^b^**	15.57 ± 1.08	14.83 ± 1.12	8.59 ± 0.38
**20**	>100	38.29 ± 2.25	19.83 ± 0.95				

^a^ IC_50_ concentration represents the concentration of a compound that produces a 50% reduction in cancer cell growth after 48 h of incubation. The given values are mean values of three experiments. ^b^ Cisplatin was used as a positive control.

## Data Availability

Not applicable.

## Supplementary Materials

The following supporting information can be downloaded at: https://www.mdpi.com/article/10.3390/ijms241915012/s1.
