# Supplementary material for: Synthesis and Antiviral and Antitumor Activities of Novel 18β-Glycyrrhetinic Acid Derivatives
Source: Int J Mol Sci. 2023 Oct 9;24(19):15012. doi: 10.3390/ijms241915012 (PMC10573640; doi:10.3390/ijms241915012)
Supplement: Supplementary file 1 [file ijms-24-15012-s001.zip › ijms-2639088-supplementary.pdf]

Synthesis and Antiviral and Antitumor Activities of Novel 18*β*-Glycyrrhetinic Acid Derivatives

Bo-Wen Pan [1,†], Liang-Liang Zheng [1,†], Yang Shi [1], Zhang-Chao Dong [1], Ting-Ting Feng [1], Jian Yang [2], Ying Wei [1,*] and Ying Zhou [1,*]

[1] College of Pharmacy, Guizhou University of Traditional Chinese Medicine, Guiyang 550025, China; bwpan1105@163.com (B.-W.P.); zx15117508142@163.com (L.-L.Z.); xiaoyewater@163.com (Y.S.); dongzhangchao0824@163.com (Z.-C.D.); ftt0809@163.com (T.-T.F.)
[2] College of Pharmacy and Nutrition, University of Saskatchewan, Saskatoon, SK S7N 5E5, Canada; jian.yang@usask.ca
* Correspondence: weiying479@gzy.edu.cn (Y.W.); zhouying@gzy.edu.cn (Y.Z.)
† These authors contributed equally to this work.

Contents | Page

1. X-ray crystallographic data of compound **12** — 2–10

2. NMR spectra — 11-80

1. X-ray crystallographic data of 12

Data intensity of **12**[1] was collected using a 'XtaLAB AFC12 (RINC)' diffractometer at 169.99(10) K. Data collection and reduction were done by using Olex2 and the structure was solved with the ShelXS structure solution program using Intrinsic Phasing and refined with the ShelXL refinement package using Least Squares minimization. Crystal data for **12**: $C_{39}H_{54}ClNO_4$, T = 169.99(10) K, orthorhombic, $P2_12_12_1$, a = 7.4071(4) Å, b = 11.548(3) Å, c = 39.996(5) Å, α = 90°, β = 90°, γ = 90°, V = 3421.1(9) $Å^3$. Z = 4, ρ_{calc} = 1.235 g/cm^3. 8460 reflections collected, 5441 [R_{int} = 0.1077, R_{sigma} = 0.1236] independent reflections, R_1 = 0.1399, wR_2 = 0.3426 ($I > 2\sigma(I)$, final), R_1 = 0.1546, wR_2 = 0.3670 (all data), GOF = 1.287, and 414 parameters.

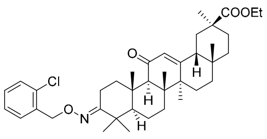

12

≡

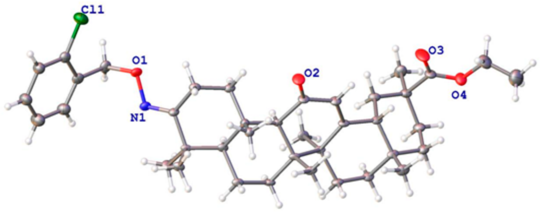

Table S1 Crystal data and structure refinement for 12

Identification code	12
Empirical formula	$C_{39}H_{54}ClNO_4$
Formula weight	636.28
Temperature/K	169.99(10)
Crystal system	orthorhombic
Space group	$P2_12_12_1$
a/Å	7.4071(4)
b/Å	11.548(3)
c/Å	39.996(5)
α/°	90
β/°	90
γ/°	90
Volume/$Å^3$	3421.1(9)
Z	4
$\rho_{calc}g/cm^3$	1.235
μ/mm^{-1}	1.306
F(000)	1376.0
Crystal size/mm^3	0.13 × 0.1 × 0.08
Radiation	Cu Kα (λ = 1.54184)
2Θ range for data collection/°	4.418 to 133.138

[1] Supplementary crystallographic data have been deposited at Cambridge Crystallographic Data Center (CCDC number: 2287206).

Index ranges	$-4 \leq h \leq 8$, $-13 \leq k \leq 12$, $-46 \leq l \leq 47$
Reflections collected	8460
Independent reflections	5441 [R_{int} = 0.1077, R_{sigma} = 0.1236]
Data/restraints/parameters	5441/408/414
Goodness-of-fit on F^2	1.287
Final R indexes [I>=2σ (I)]	R_1 = 0.1399, wR_2 = 0.3426
Final R indexes [all data]	R_1 = 0.1546, wR_2 = 0.3670
Largest diff. peak/hole / e Å^{-3}	0.74/-0.80
Flack parameter	0.04(7)

Table S2 Fractional Atomic Coordinates (×10^4) and Equivalent Isotropic Displacement Parameters (Å^2×10^3) for 12. U_{eq} is defined as 1/3 of the trace of the orthogonalised U_{IJ} tensor.

Atom	*x*	*y*	*z*	U(eq)
Cl1	158(2)	6350.3(15)	9353.1(4)	53.9(4)
O1	3386(5)	5544(3)	8750.1(9)	33.7(7)
O2	4339(5)	7945(4)	7210.3(10)	41.9(9)
O3	3735(6)	6162(5)	5756.4(12)	61.8(10)
O4	5257(6)	5482(4)	5320.7(10)	46.9(9)
N1	5270(6)	5440(4)	8673.7(10)	29.4(7)
C1	3476(8)	5277(5)	9354.1(14)	35.6(8)
C2	5078(9)	5032(6)	9508.1(16)	47.0(11)
C3	5509(10)	5463(7)	9821.3(16)	57.5(12)
C4	4292(9)	6155(6)	9983.2(16)	48.7(11)
C5	2642(9)	6428(6)	9839.2(14)	43.4(11)
C6	2205(8)	6001(5)	9525.1(14)	35.2(8)
C7	2994(8)	4779(5)	9019.2(13)	36.6(10)
C8	5676(6)	6074(4)	8421.9(12)	25.1(7)
C9	7680(5)	6089(4)	8318.9(11)	21.3(6)
C10	7833(6)	6100(4)	7933.0(11)	24.0(7)
C11	6533(6)	6915(4)	7739.5(11)	23.8(4)
C12	4601(6)	6681(4)	7857.7(12)	26.6(8)
C13	4376(6)	6813(4)	8233.5(12)	27.3(8)
C14	9771(6)	6237(4)	7809.1(12)	25.8(8)
C15	9949(6)	5867(4)	7449.3(12)	26.6(8)
C16	8614(6)	6471(4)	7206.3(12)	22.6(6)
C17	6696(6)	6580(4)	7368.4(11)	19.7(7)
C18	5479(6)	7261(4)	7122.9(13)	29.3(7)
C19	5750(7)	6993(5)	6769.5(13)	31.4(8)
C20	7047(6)	6289(4)	6647.3(12)	25.8(7)
C21	8415(6)	5722(4)	6880.3(12)	21.3(6)

C22	10266(6)	5569(4)	6708.4(12)	28.9(8)
C23	10139(7)	5159(4)	6350.4(12)	29.5(8)
C24	8970(6)	5963(4)	6129.8(13)	26.4(6)
C25	7048(6)	6026(4)	6278.4(12)	26.1(7)
C26	5871(7)	4946(4)	6209.7(13)	29.9(8)
C27	5837(6)	4565(5)	5847.2(13)	29.8(7)
C28	7763(7)	4429(5)	5722.9(13)	34.7(10)
C29	8896(7)	5491(5)	5771.2(13)	34.8(8)
C30	4838(7)	5506(5)	5640.3(13)	37.0(8)
C31	4716(9)	3446(5)	5808.7(15)	43.2(12)
C32	4364(11)	6349(6)	5112.4(17)	59.3(12)
C34	5364(15)	6579(9)	4814(2)	80(2)
C35	9800(8)	7165(4)	6121.6(14)	38.0(12)
C36	7675(7)	4494(4)	6956.9(12)	28.2(10)
C37	9362(6)	7675(4)	7129.3(13)	27.8(10)
C38	6952(7)	8234(4)	7790.8(11)	27.1(7)
C39	8577(7)	7143(4)	8489.1(12)	30.8(10)
C40	8609(7)	4985(4)	8449.8(13)	33.5(11)

Table S3 Anisotropic Displacement Parameters (Å^2×10^3) for12. The Anisotropic displacement factor exponent takes the form: $-2\pi^2[h^2a^{*2}U_{11}+2hka^*b^*U_{12}+\ldots]$.

Atom	U_{11}	U_{22}	U_{33}	U_{23}	U_{13}	U_{12}
Cl1	46.0(6)	48.6(9)	67.0(8)	-0.7(7)	-14.5(6)	4.7(7)
O1	24.3(10)	29.9(16)	46.8(14)	8.7(11)	1.9(11)	-7.1(13)
O2	37.7(14)	35.7(15)	52.3(19)	0.7(14)	3.1(13)	24.7(10)
O3	47.8(15)	76(2)	62(2)	-2.3(17)	-5.6(14)	44.3(12)
O4	47.4(19)	46.4(19)	46.9(11)	8.0(12)	0.4(12)	21.1(16)
N1	24.3(11)	21.6(16)	42.4(13)	2.1(10)	0.9(11)	-4.3(13)
C1	36.5(13)	23.3(19)	46.9(13)	0.6(12)	3.4(10)	-10.9(12)
C2	41.0(14)	43(3)	57.1(14)	-5.4(15)	-2.2(12)	-2.3(19)
C3	51(2)	64(3)	57.6(15)	-9.7(15)	-6.4(14)	5.5(16)
C4	44.3(13)	52(3)	50(2)	-2.7(18)	-7.7(12)	0.0(14)
C5	41.0(14)	46(3)	43.6(14)	-3.1(14)	-1.7(12)	0.6(19)
C6	37.6(14)	25.4(19)	42.6(13)	4.0(12)	0.8(10)	-6.3(13)
C7	45(2)	21.8(19)	43.4(12)	3.7(10)	3.8(13)	-12.0(19)
C8	20.5(10)	16.6(15)	38.3(13)	-1.0(10)	2.5(10)	3.0(11)
C9	15.7(10)	7.6(11)	40.6(11)	0.1(11)	-2.8(10)	0.2(10)
C10	20.7(11)	10.4(16)	40.9(11)	-1.0(12)	-2.7(10)	-3.6(11)
C11	22.8(7)	22.3(7)	26.4(7)	-0.2(6)	0.0(6)	0.5(6)
C12	20.7(12)	14(2)	44.8(12)	-0.1(15)	1.7(12)	1.9(14)

C13	24.3(15)	13.2(18)	44.5(12)	-2.1(13)	-0.2(12)	3.3(13)
C14	20.4(12)	10.6(19)	46.3(12)	0.2(13)	-1.5(12)	0.5(15)
C15	14.6(14)	18.0(19)	47.3(12)	-2.7(12)	-3.5(11)	1.5(14)
C16	15.8(11)	8.2(11)	43.7(13)	-1.0(9)	-0.1(9)	-0.3(10)
C17	15.9(11)	10.0(17)	33.3(10)	-2.4(10)	-2.1(9)	-2.0(12)
C18	22.1(13)	21.2(15)	44.6(11)	3.0(11)	-0.2(10)	11.0(10)
C19	25.0(15)	23.8(18)	45.4(12)	0.4(13)	0.3(12)	11.6(12)
C20	17.1(12)	14.8(16)	45.4(11)	2.5(11)	-0.7(9)	5.7(10)
C21	17.1(11)	4.2(11)	42.5(12)	2.5(9)	-0.2(9)	2.4(10)
C22	18.4(12)	19(2)	49.2(13)	-0.9(14)	2.2(11)	4.1(15)
C23	25.8(17)	15.1(17)	47.6(12)	1.9(11)	1.9(13)	6.7(14)
C24	21.9(11)	11.0(12)	46.2(12)	3.8(11)	5.1(11)	5.3(10)
C25	20.5(11)	12.1(14)	45.5(12)	3.5(13)	2.6(11)	5.5(11)
C26	23.4(17)	18.5(15)	47.7(13)	-2.5(13)	-1.4(15)	2.1(12)
C27	22.6(11)	20.5(13)	46.4(12)	-0.2(11)	-2.2(11)	3.9(10)
C28	26.8(12)	31.0(16)	46(2)	-4.9(19)	2.1(14)	7.4(12)
C29	28.7(17)	29.4(16)	46.2(14)	-0.4(14)	4.8(16)	6.5(13)
C30	24.4(15)	39.5(16)	47.1(11)	4.1(11)	-3.4(11)	14.1(11)
C31	48(2)	32.4(16)	50(3)	-6(2)	3(2)	-13.2(14)
C32	60(3)	53(2)	65.5(17)	18.8(15)	-12.3(14)	16.6(19)
C34	86(4)	76(5)	78(2)	20(3)	0.8(17)	-5(4)
C35	36(2)	14.5(13)	64(3)	5.7(19)	5(2)	0.0(14)
C36	33(2)	8.7(12)	43(2)	7.2(15)	-0.6(18)	-4.8(13)
C37	22.2(17)	7.6(12)	54(2)	-0.4(16)	4.6(17)	-0.1(13)
C38	28.0(13)	22.6(10)	30.7(13)	0.0(10)	1.1(11)	1.6(10)
C39	26.9(19)	19.9(16)	46(2)	-8.7(14)	-7.3(16)	-3.6(14)
C40	33(2)	20.6(15)	47(2)	6.9(15)	-3.1(18)	12.1(14)

Table S4 Bond Lengths for 12.

Atom	Atom	Length/Å	Atom	Atom	Length/Å
Cl1	C6	1.713(6)	C12	C13	1.520(7)
O1	N1	1.434(5)	C14	C15	1.507(7)
O1	C7	1.423(6)	C15	C16	1.552(6)
O2	C18	1.208(6)	C16	C17	1.567(6)
O3	C30	1.207(7)	C16	C21	1.572(6)
O4	C30	1.316(7)	C16	C37	1.528(6)
O4	C32	1.461(8)	C17	C18	1.548(6)
N1	C8	1.281(6)	C18	C19	1.461(7)
C1	C2	1.367(9)	C19	C20	1.350(7)
C1	C6	1.433(8)	C20	C21	1.524(6)

C1	C7	1.501(8)	C20	C25	1.506(7)
C2	C3	1.385(9)	C21	C22	1.544(6)
C3	C4	1.367(10)	C21	C36	1.551(6)
C4	C5	1.388(9)	C22	C23	1.511(7)
C5	C6	1.388(8)	C23	C24	1.546(7)
C8	C9	1.540(6)	C24	C25	1.545(6)
C8	C13	1.491(7)	C24	C29	1.536(7)
C9	C10	1.548(6)	C24	C35	1.519(7)
C9	C39	1.545(6)	C25	C26	1.546(7)
C9	C40	1.540(6)	C26	C27	1.515(7)
C10	C11	1.553(6)	C27	C28	1.519(7)
C10	C14	1.527(6)	C27	C30	1.553(7)
C11	C12	1.531(6)	C27	C31	1.544(8)
C11	C17	1.539(6)	C28	C29	1.498(8)
C11	C38	1.568(7)	C32	C34	1.431(11)

Table S5 Bond Angles for 12.

Atom	Atom	Atom	Angle/°	Atom	Atom	Atom	Angle/°
C7	O1	N1	107.9(4)	C37	C16	C21	111.6(4)
C30	O4	C32	115.7(5)	C11	C17	C16	119.4(4)
C8	N1	O1	110.4(4)	C11	C17	C18	116.0(4)
C2	C1	C6	118.4(5)	C18	C17	C16	107.8(4)
C2	C1	C7	122.0(5)	O2	C18	C17	123.8(5)
C6	C1	C7	119.6(5)	O2	C18	C19	121.0(5)
C1	C2	C3	122.3(6)	C19	C18	C17	115.2(4)
C4	C3	C2	119.1(7)	C20	C19	C18	125.2(5)
C3	C4	C5	121.1(6)	C19	C20	C21	120.7(4)
C4	C5	C6	120.0(6)	C19	C20	C25	118.4(4)
C1	C6	Cl1	121.8(4)	C25	C20	C21	120.8(4)
C5	C6	Cl1	119.1(5)	C20	C21	C16	109.5(4)
C5	C6	C1	119.1(5)	C20	C21	C22	111.5(4)
O1	C7	C1	112.8(4)	C20	C21	C36	106.2(4)
N1	C8	C9	116.3(4)	C22	C21	C16	110.4(4)
N1	C8	C13	124.9(4)	C22	C21	C36	107.3(4)
C13	C8	C9	118.7(4)	C36	C21	C16	111.9(4)
C8	C9	C10	109.7(4)	C23	C22	C21	113.7(4)
C8	C9	C39	107.8(4)	C22	C23	C24	112.8(4)
C39	C9	C10	113.7(4)	C25	C24	C23	108.9(4)
C40	C9	C8	109.3(4)	C29	C24	C23	109.9(4)
C40	C9	C10	108.2(4)	C29	C24	C25	110.1(4)

C40	C9	C39	108.1(4)	C35	C24	C23	109.5(4)
C9	C10	C11	117.2(4)	C35	C24	C25	109.8(4)
C14	C10	C9	113.2(4)	C35	C24	C29	108.6(4)
C14	C10	C11	111.0(4)	C20	C25	C24	112.7(4)
C10	C11	C38	113.6(4)	C20	C25	C26	109.7(4)
C12	C11	C10	108.6(4)	C24	C25	C26	114.4(4)
C12	C11	C17	109.1(4)	C27	C26	C25	114.4(4)
C12	C11	C38	108.4(4)	C26	C27	C28	109.1(4)
C17	C11	C10	106.2(4)	C26	C27	C30	108.4(4)
C17	C11	C38	110.8(4)	C26	C27	C31	110.3(4)
C13	C12	C11	112.9(4)	C28	C27	C30	110.2(4)
C8	C13	C12	111.8(4)	C28	C27	C31	112.7(5)
C15	C14	C10	111.3(4)	C31	C27	C30	106.0(4)
C14	C15	C16	114.5(4)	C29	C28	C27	113.5(5)
C15	C16	C17	110.8(4)	C28	C29	C24	115.5(4)
C15	C16	C21	109.4(4)	O3	C30	O4	123.1(5)
C17	C16	C21	107.6(3)	O3	C30	C27	123.8(5)
C37	C16	C15	107.7(4)	O4	C30	C27	113.0(5)
C37	C16	C17	109.8(4)	C34	C32	O4	111.7(7)

Table S6 Torsion Angles for 12.

A	B	C	D	Angle/°	A	B	C	D	Angle/°
O1	N1	C8	C9	177.6(4)	C17	C18	C19	C20	-5.6(7)
O1	N1	C8	C13	-0.2(7)	C18	C19	C20	C21	-1.7(8)
O2	C18	C19	C20	176.4(5)	C18	C19	C20	C25	175.1(5)
N1	O1	C7	C1	81.6(5)	C19	C20	C21	C16	-25.0(6)
N1	C8	C9	C10	141.1(4)	C19	C20	C21	C22	-147.5(5)
N1	C8	C9	C39	-94.7(5)	C19	C20	C21	C36	96.0(5)
N1	C8	C9	C40	22.5(6)	C19	C20	C25	C24	142.0(5)
N1	C8	C13	C12	-134.4(5)	C19	C20	C25	C26	-89.2(5)
C1	C2	C3	C4	0.1(11)	C20	C21	C22	C23	-41.9(5)
C2	C1	C6	Cl1	-179.7(5)	C20	C25	C26	C27	-177.4(4)
C2	C1	C6	C5	0.1(9)	C21	C16	C17	C11	160.5(4)
C2	C1	C7	O1	-96.4(7)	C21	C16	C17	C18	-64.3(4)
C2	C3	C4	C5	0.1(11)	C21	C20	C25	C24	-41.3(6)
C3	C4	C5	C6	-0.2(11)	C21	C20	C25	C26	87.5(5)
C4	C5	C6	Cl1	179.9(5)	C21	C22	C23	C24	56.3(5)
C4	C5	C6	C1	0.1(9)	C22	C23	C24	C25	-59.1(5)
C6	C1	C2	C3	-0.2(10)	C22	C23	C24	C29	-179.8(4)
C6	C1	C7	O1	85.8(6)	C22	C23	C24	C35	61.0(5)

C7	O1	N1	C8	177.0(4)
C7	C1	C2	C3	-178.1(6)
C7	C1	C6	Cl1	-1.7(7)
C7	C1	C6	C5	178.0(5)
C8	C9	C10	C11	43.1(5)
C8	C9	C10	C14	174.3(4)
C9	C8	C13	C12	47.8(6)
C9	C10	C11	C12	-51.7(5)
C9	C10	C11	C17	-168.8(4)
C9	C10	C11	C38	69.1(5)
C9	C10	C14	C15	161.8(4)
C10	C11	C12	C13	56.0(5)
C10	C11	C17	C16	-49.6(5)
C10	C11	C17	C18	178.6(4)
C10	C14	C15	C16	53.9(5)
C11	C10	C14	C15	-64.1(5)
C11	C12	C13	C8	-55.0(5)
C11	C17	C18	O2	-6.5(7)
C11	C17	C18	C19	175.6(4)
C12	C11	C17	C16	-166.5(4)
C12	C11	C17	C18	61.7(5)
C13	C8	C9	C10	-40.9(6)
C13	C8	C9	C39	83.3(5)
C13	C8	C9	C40	-159.5(4)
C14	C10	C11	C12	176.2(4)
C14	C10	C11	C17	59.0(5)
C14	C10	C11	C38	-63.1(5)
C14	C15	C16	C17	-40.8(5)
C14	C15	C16	C21	-159.2(4)
C14	C15	C16	C37	79.4(5)
C15	C16	C17	C11	41.0(5)
C15	C16	C17	C18	176.2(4)
C15	C16	C21	C20	177.7(4)
C15	C16	C21	C22	-59.1(5)
C15	C16	C21	C36	60.3(5)
C16	C17	C18	O2	-143.4(5)
C16	C17	C18	C19	38.6(5)
C16	C21	C22	C23	-163.8(4)
C17	C11	C12	C13	171.4(4)
C17	C16	C21	C20	57.4(4)
C17	C16	C21	C22	-179.5(4)
C17	C16	C21	C36	-60.0(5)
C23	C24	C25	C20	49.8(5)
C23	C24	C25	C26	-76.5(5)
C23	C24	C29	C28	72.8(5)
C24	C25	C26	C27	-49.6(6)
C25	C20	C21	C16	158.3(4)
C25	C20	C21	C22	35.9(6)
C25	C20	C21	C36	-80.7(5)
C25	C24	C29	C28	-47.1(6)
C25	C26	C27	C28	52.5(6)
C25	C26	C27	C30	-67.5(5)
C25	C26	C27	C31	176.9(4)
C26	C27	C28	C29	-54.7(6)
C26	C27	C30	O3	-25.1(7)
C26	C27	C30	O4	157.8(5)
C27	C28	C29	C24	54.4(6)
C28	C27	C30	O3	-144.4(6)
C28	C27	C30	O4	38.4(6)
C29	C24	C25	C20	170.3(4)
C29	C24	C25	C26	44.0(5)
C30	O4	C32	C34	156.8(7)
C30	C27	C28	C29	64.2(6)
C31	C27	C28	C29	-177.6(5)
C31	C27	C30	O3	93.3(7)
C31	C27	C30	O4	-83.8(6)
C32	O4	C30	O3	3.0(9)
C32	O4	C30	C27	-179.9(5)
C35	C24	C25	C20	-70.2(5)
C35	C24	C25	C26	163.6(4)
C35	C24	C29	C28	-167.4(4)
C36	C21	C22	C23	74.0(5)
C37	C16	C17	C11	-77.9(5)
C37	C16	C17	C18	57.4(5)
C37	C16	C21	C20	-63.2(5)
C37	C16	C21	C22	60.0(5)
C37	C16	C21	C36	179.4(4)
C38	C11	C12	C13	-67.9(5)
C38	C11	C17	C16	74.2(5)
C38	C11	C17	C18	-57.5(5)
C39	C9	C10	C11	-77.6(5)
C39	C9	C10	C14	53.5(5)
C40	C9	C10	C11	162.3(4)
C40	C9	C10	C14	-66.5(5)

Table S7 Hydrogen Atom Coordinates (Å×10[4]) and Isotropic Displacement Parameters (Å[2]×10[3]) for 12.

Atom	*x*	*y*	*z*	U(eq)
H2	5925.37	4550.49	9396.56	56
H3	6633.76	5279.33	9922.34	69
H4	4581.92	6453.07	10197.95	58
H5	1811.7	6908.47	9955.62	52
H7A	3668.76	4047.63	8986.11	44
H7B	1689.41	4591.94	9016.39	44
H10	7474.27	5301.74	7862.59	29
H12A	3771.63	7223.17	7743.14	32
H12B	4254.69	5883.93	7792.54	32
H13A	4565.13	7634.32	8295.82	33
H13B	3128.37	6598.35	8296.84	33
H14A	10143.51	7056.35	7831.23	31
H14B	10585.56	5761.79	7949.51	31
H15A	9758.23	5019.96	7435.75	32
H15B	11196.73	6028.21	7373.84	32
H17	6206.81	5773.56	7359.6	24
H19	4951.3	7341.26	6613.34	38
H22A	10915	6318.43	6713.19	35
H22B	10988.43	5003.84	6837.72	35
H23A	9616.57	4370.39	6346.96	35
H23B	11368.87	5114.41	6254.57	35
H25	6432.06	6691.96	6166.44	31
H26A	6325.18	4296.71	6347.81	36
H26B	4618.82	5111.76	6281.94	36
H28A	8335.22	3774.21	5842.35	42
H28B	7736.03	4233.97	5481.97	42
H29A	8426.95	6108.17	5623.07	42
H29B	10142.96	5313.54	5698.27	42
H31A	5155.12	2862.05	5966.62	65
H31B	4837.05	3153	5579.87	65
H31C	3443.17	3614.03	5855.09	65
H32A	3141.9	6070.9	5052.17	71
H32B	4226.73	7075.72	5241.3	71
H34A	4603.62	7009.78	4656.16	120
H34B	5738.07	5846.12	4711.61	120
H34C	6434.73	7040.33	4868.34	120
H35A	9164.83	7641.92	5956.46	57

H35B	11077.67	7106.72	6060.03	57
H35C	9694.3	7523.27	6342.87	57
H36A	6363.72	4532.36	6987.91	42
H36B	8239.46	4197.15	7161.29	42
H36C	7953.61	3975.33	6769.88	42
H37A	10568.43	7602.57	7030.82	42
H37B	9437.68	8125.97	7336.45	42
H37C	8560.2	8070.06	6971.17	42
H38A	6474.34	8486.46	8007.34	41
H38B	6381.71	8682.99	7611.63	41
H38C	8260.42	8356.81	7785.34	41
H39A	9819.85	7223.74	8408.28	46
H39B	8586.37	7028.77	8731.93	46
H39C	7894.59	7845.67	8435.19	46
H40A	8197.52	4316.05	8319.84	50
H40B	8301.12	4872.44	8685.8	50
H40C	9920.68	5065.32	8426.88	50

2. NMR spectra

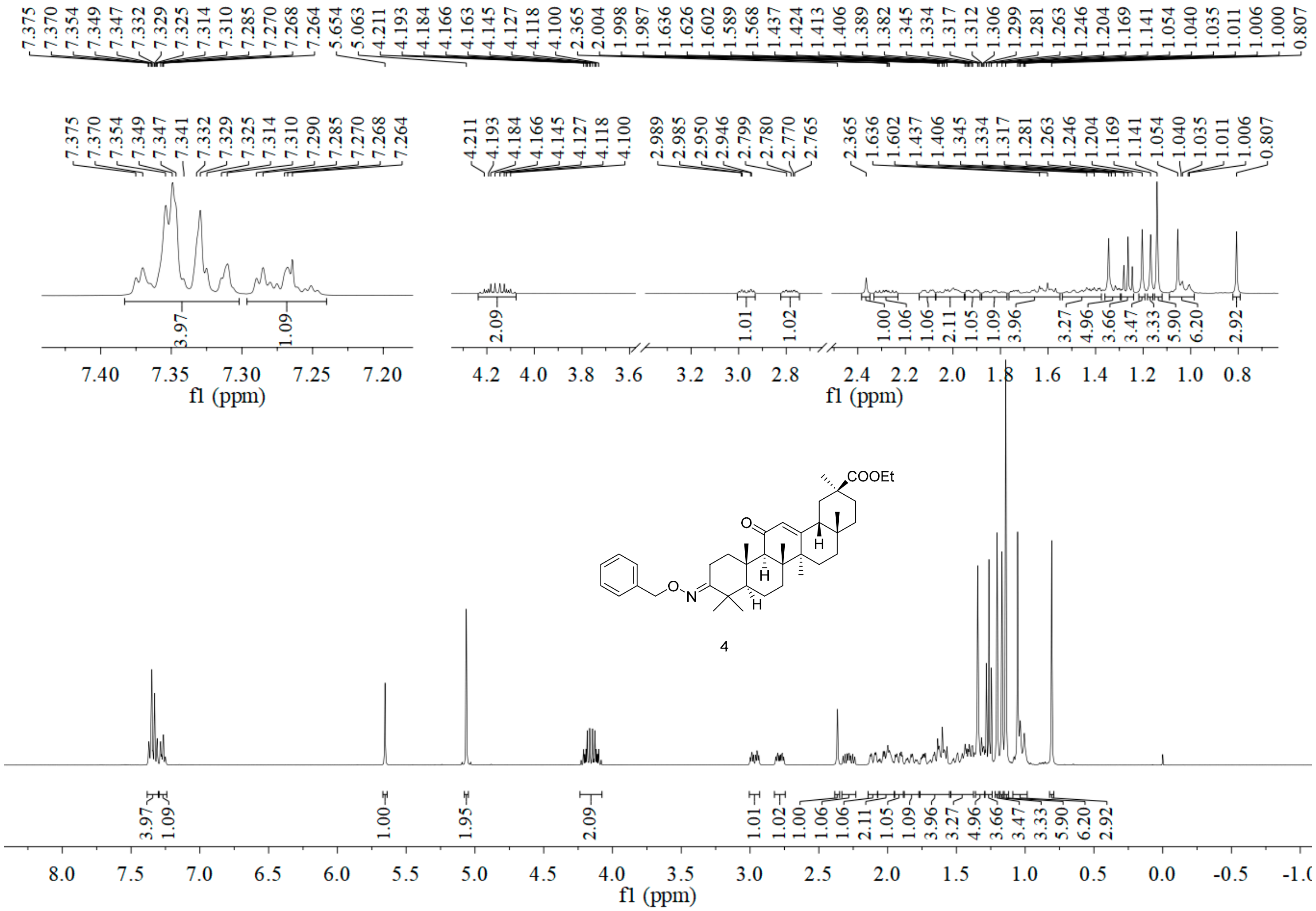

^{1}H NMR of Compound **4** (400 MHz, $CDCl_3$)

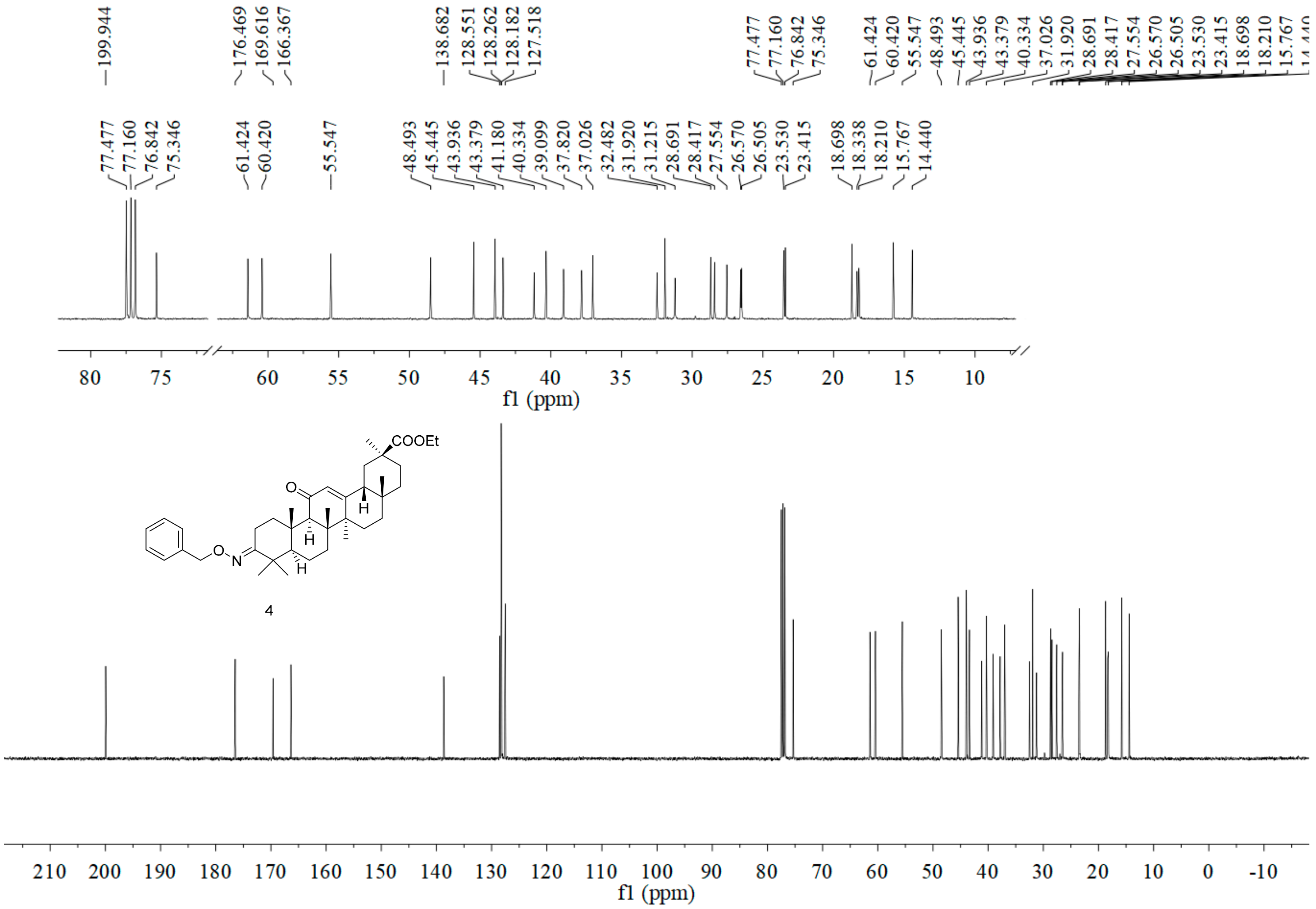

^{13}C NMR of Compound **4** (100 MHz, $CDCl_3$)

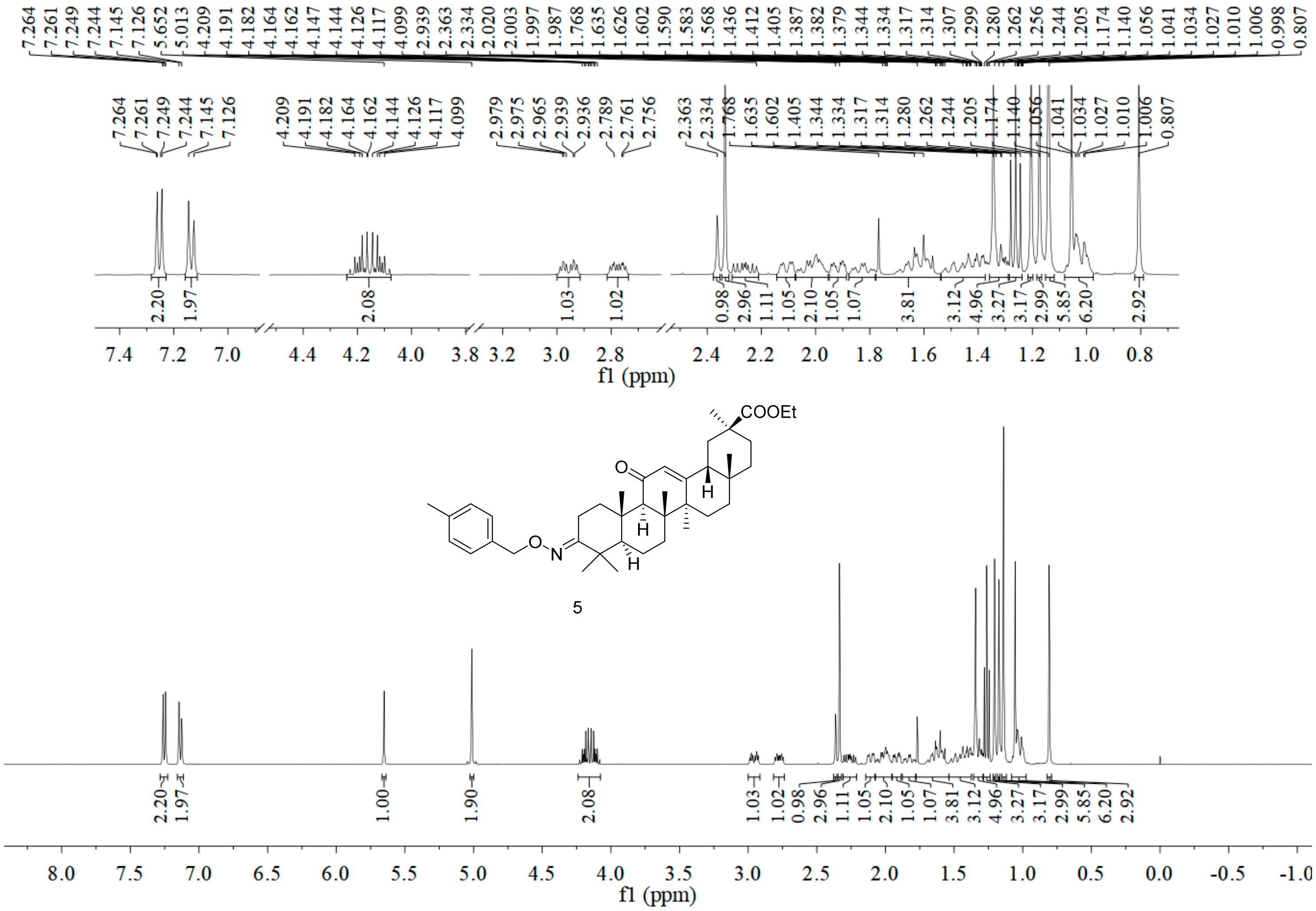

^{1}H NMR of Compound **5** (400 MHz, $CDCl_3$)

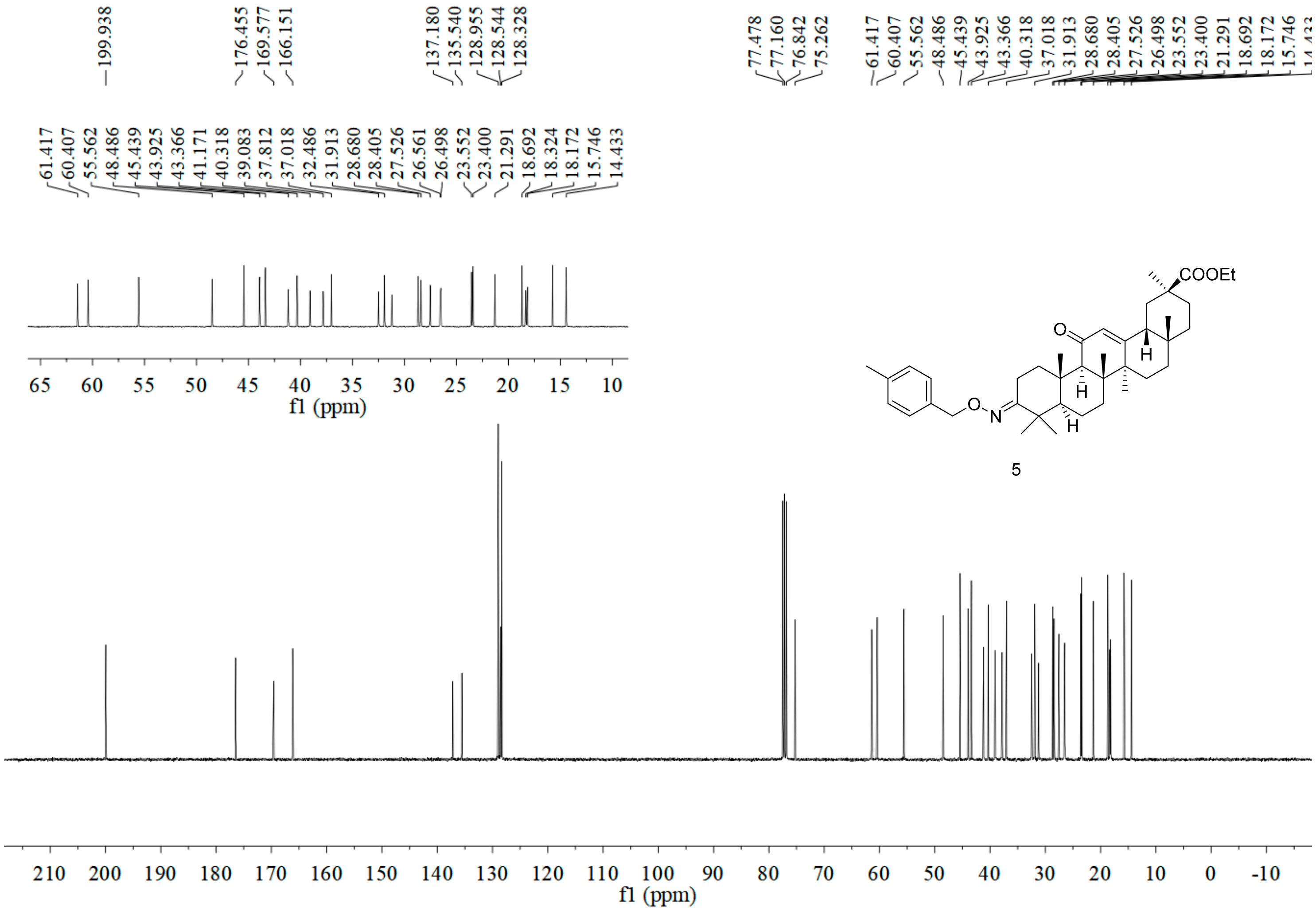

^{13}C NMR of Compound **5** (100 MHz, $CDCl_3$)

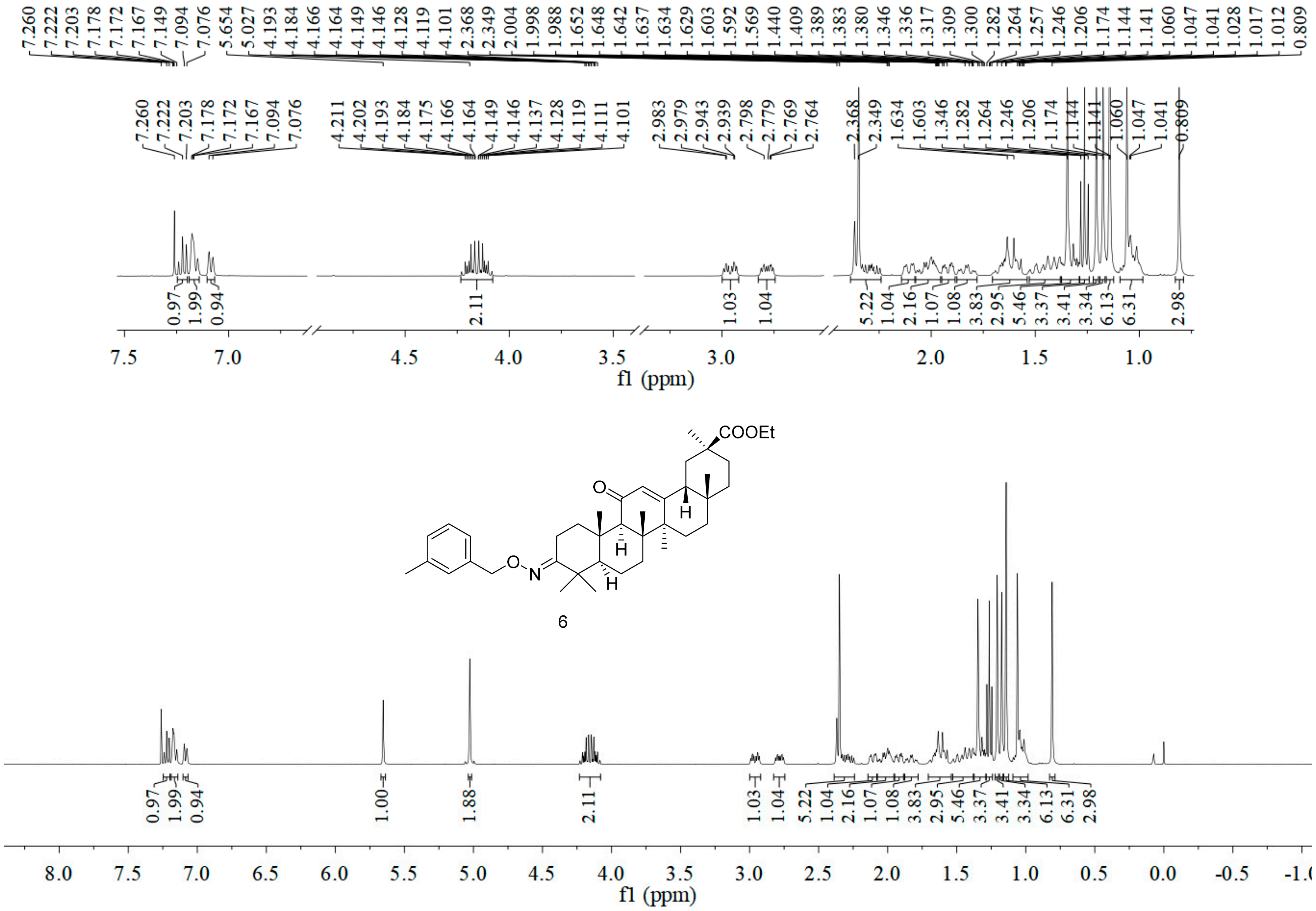

^{1}H NMR of Compound **6** (400 MHz, $CDCl_3$)

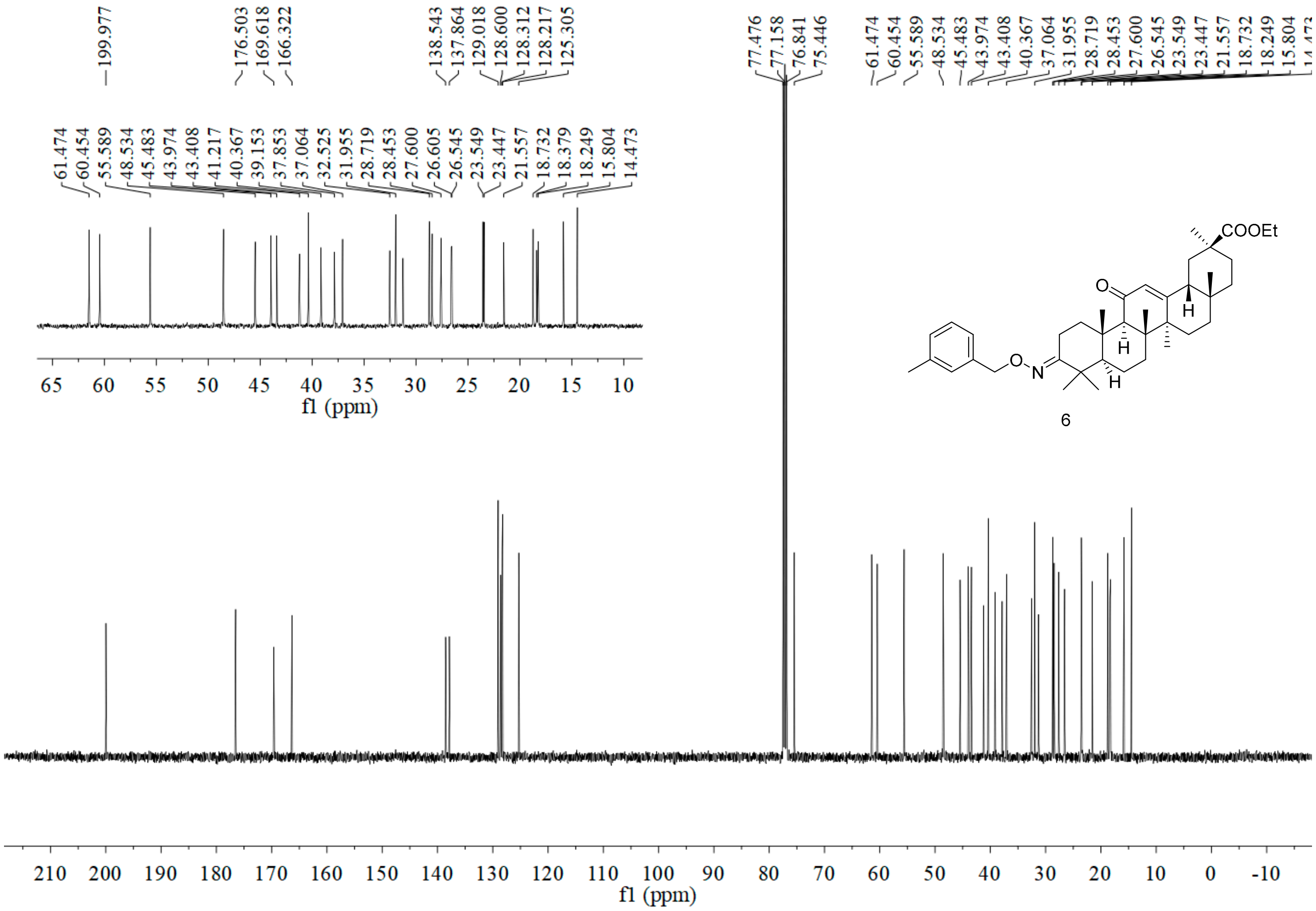

^{13}C NMR of Compound **6** (100 MHz, $CDCl_3$)

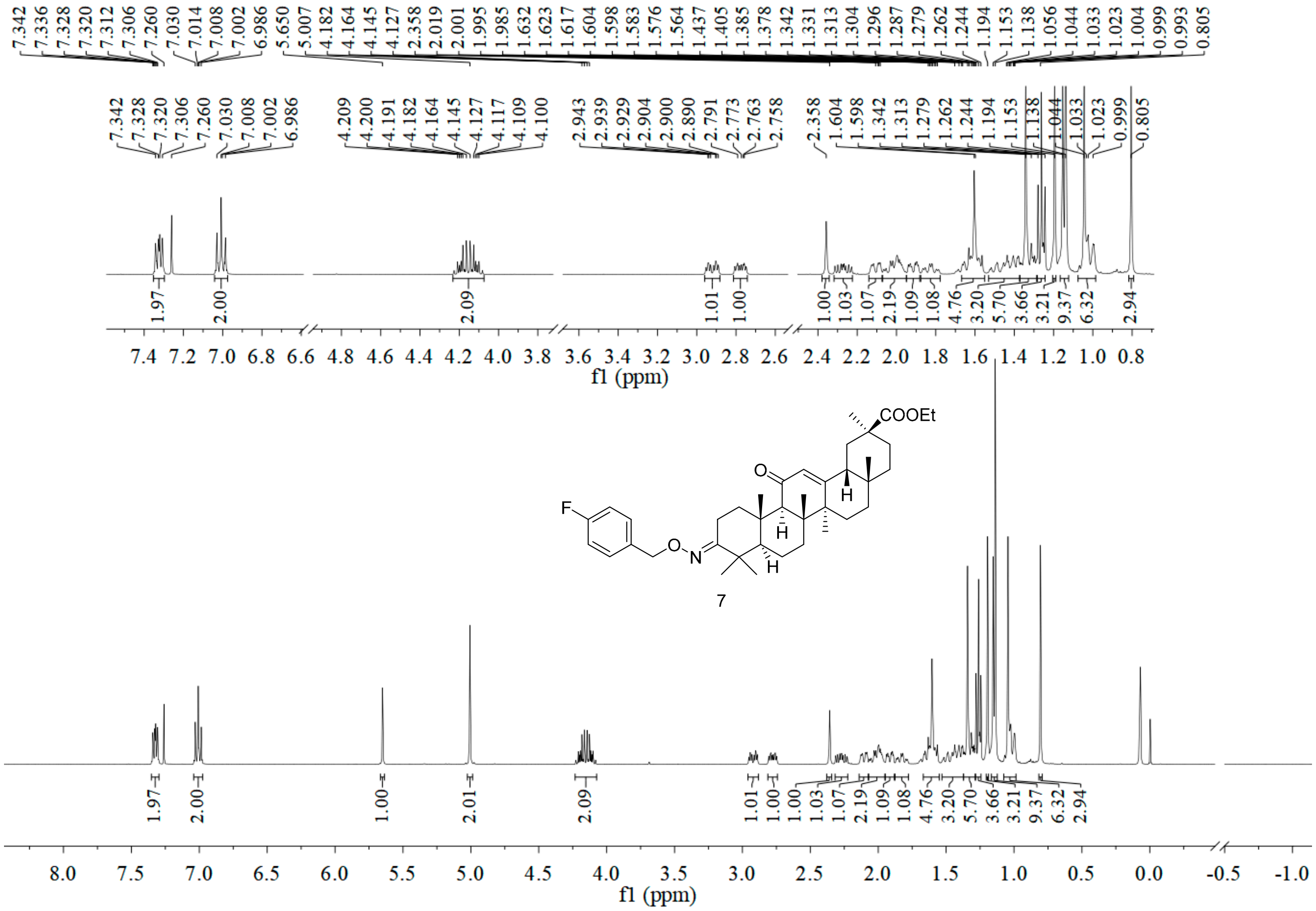

^{1}H NMR of Compound **7** (400 MHz, $CDCl_3$)

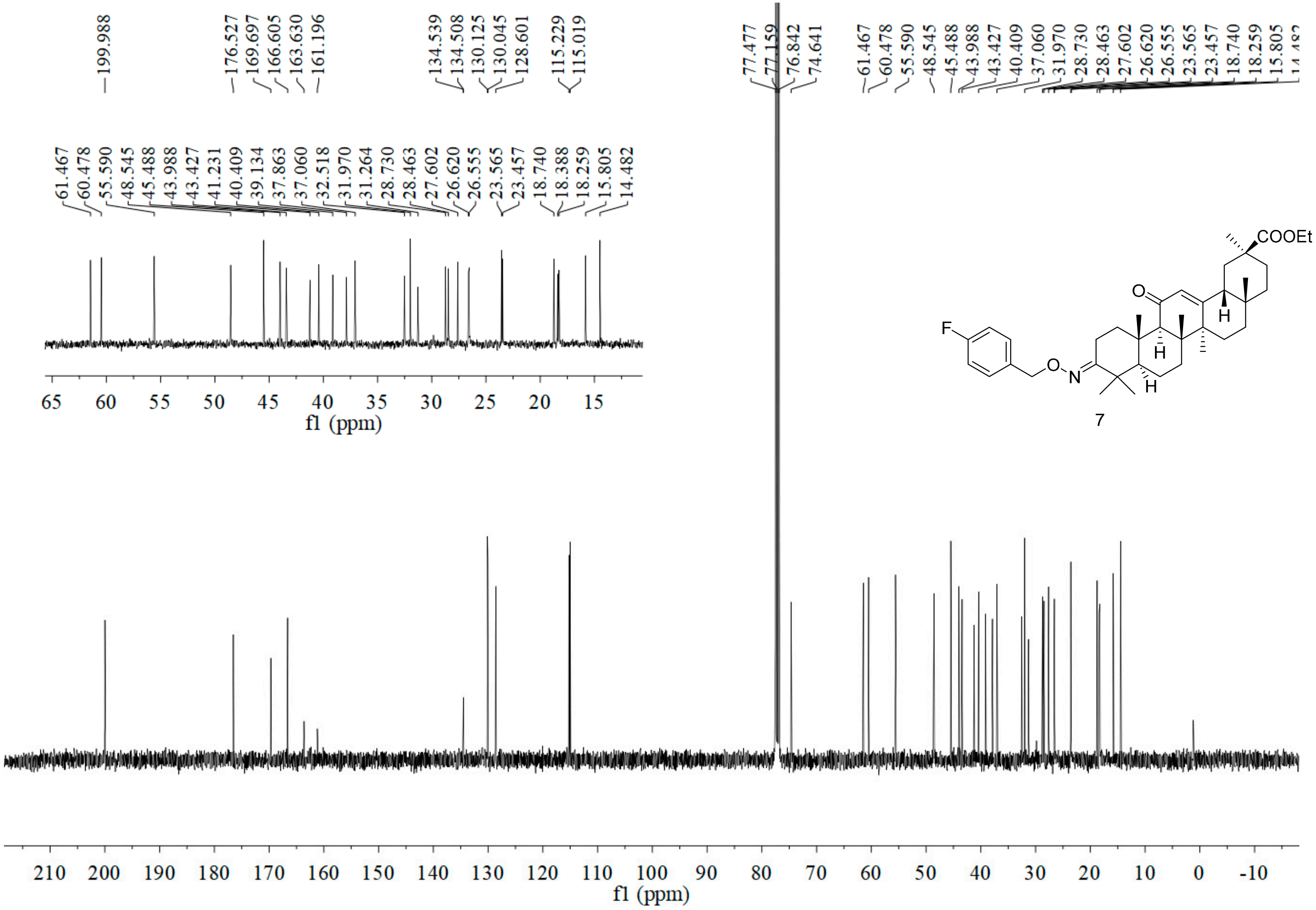

^{13}C NMR of Compound **7** (100 MHz, $CDCl_3$)

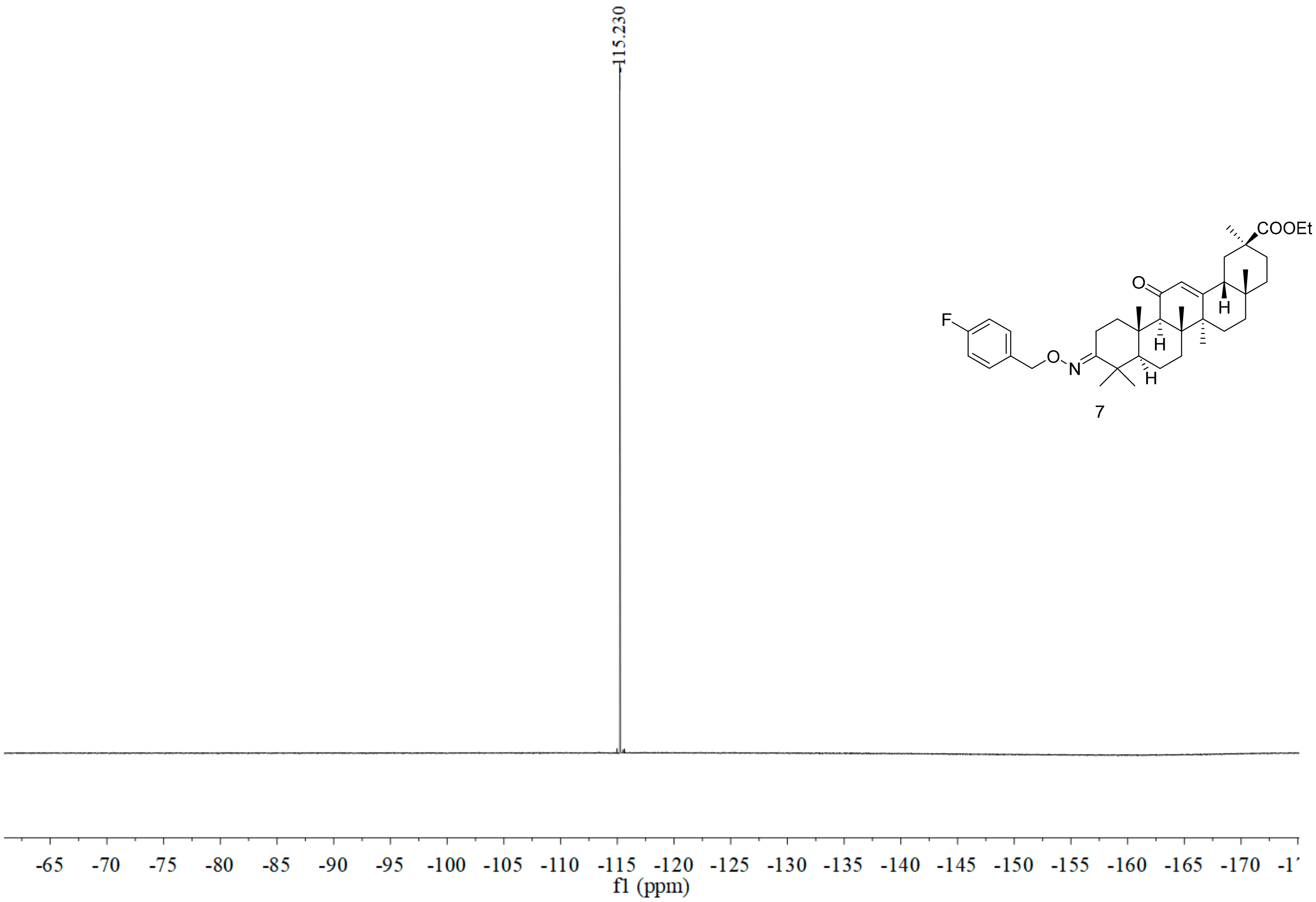

^{19}F NMR of Compound **7** (377 MHz, $CDCl_3$)

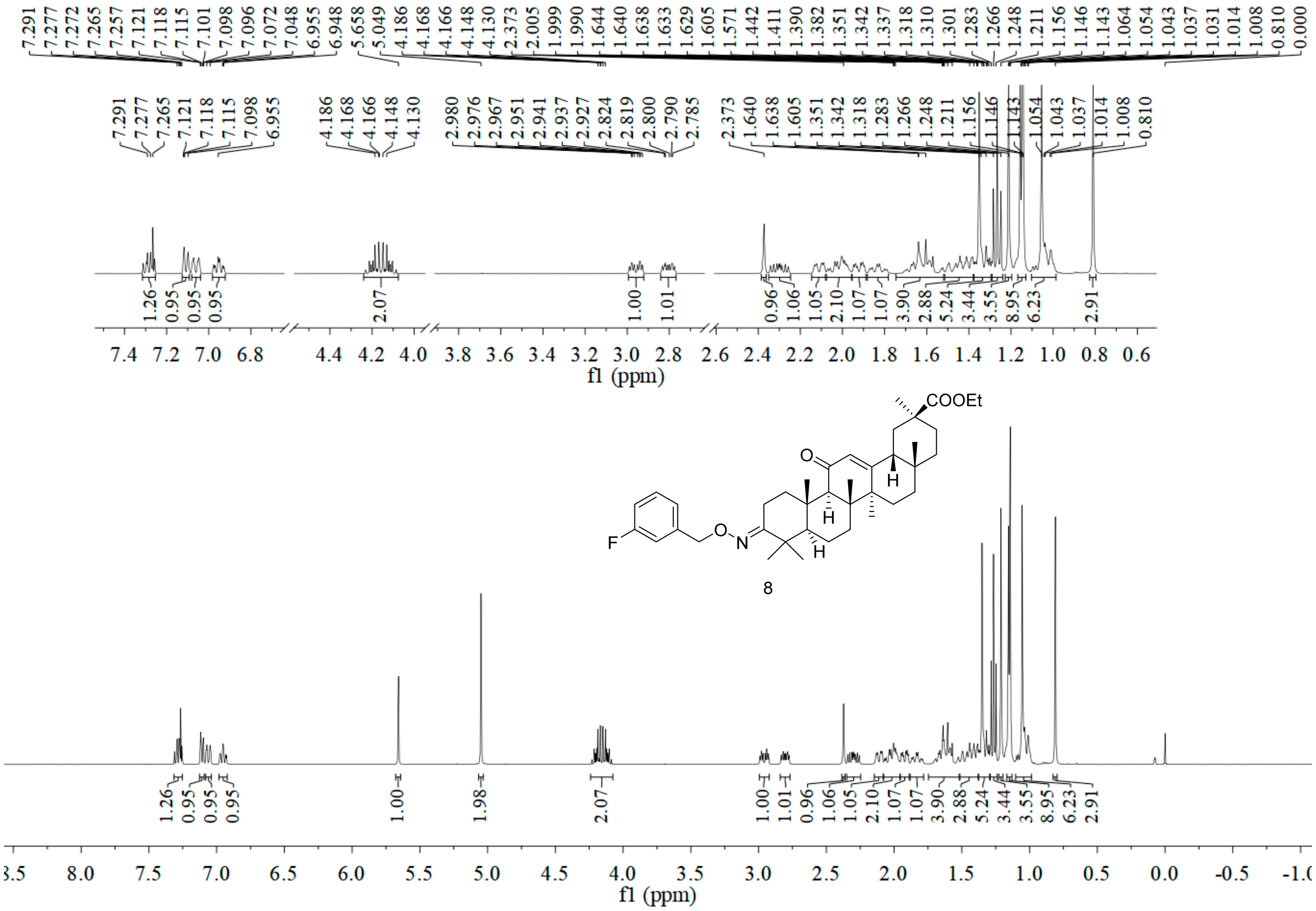

^{1}H NMR of Compound **8** (400 MHz, $CDCl_3$)

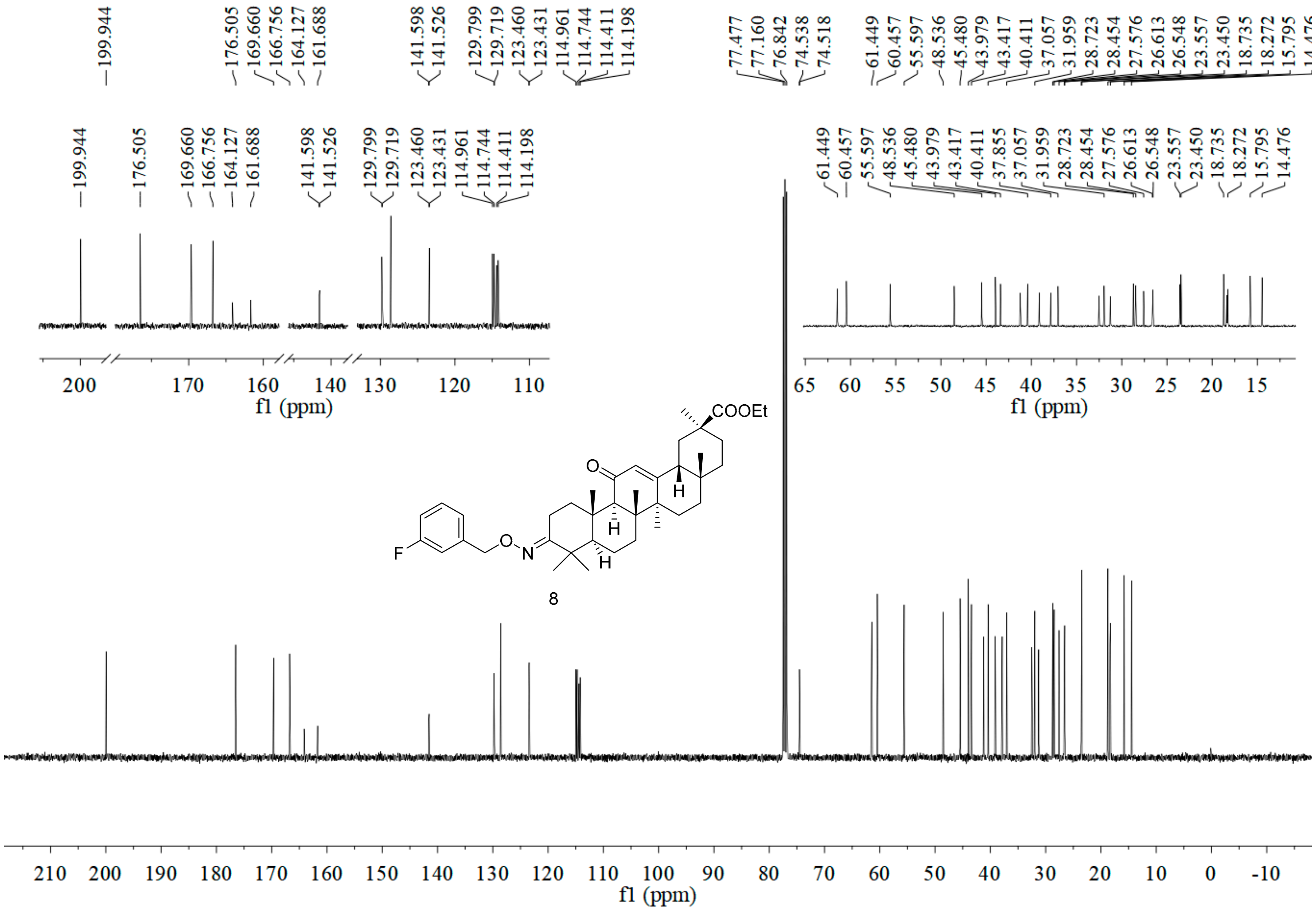

^{13}C NMR of Compound **8** (100 MHz, $CDCl_3$)

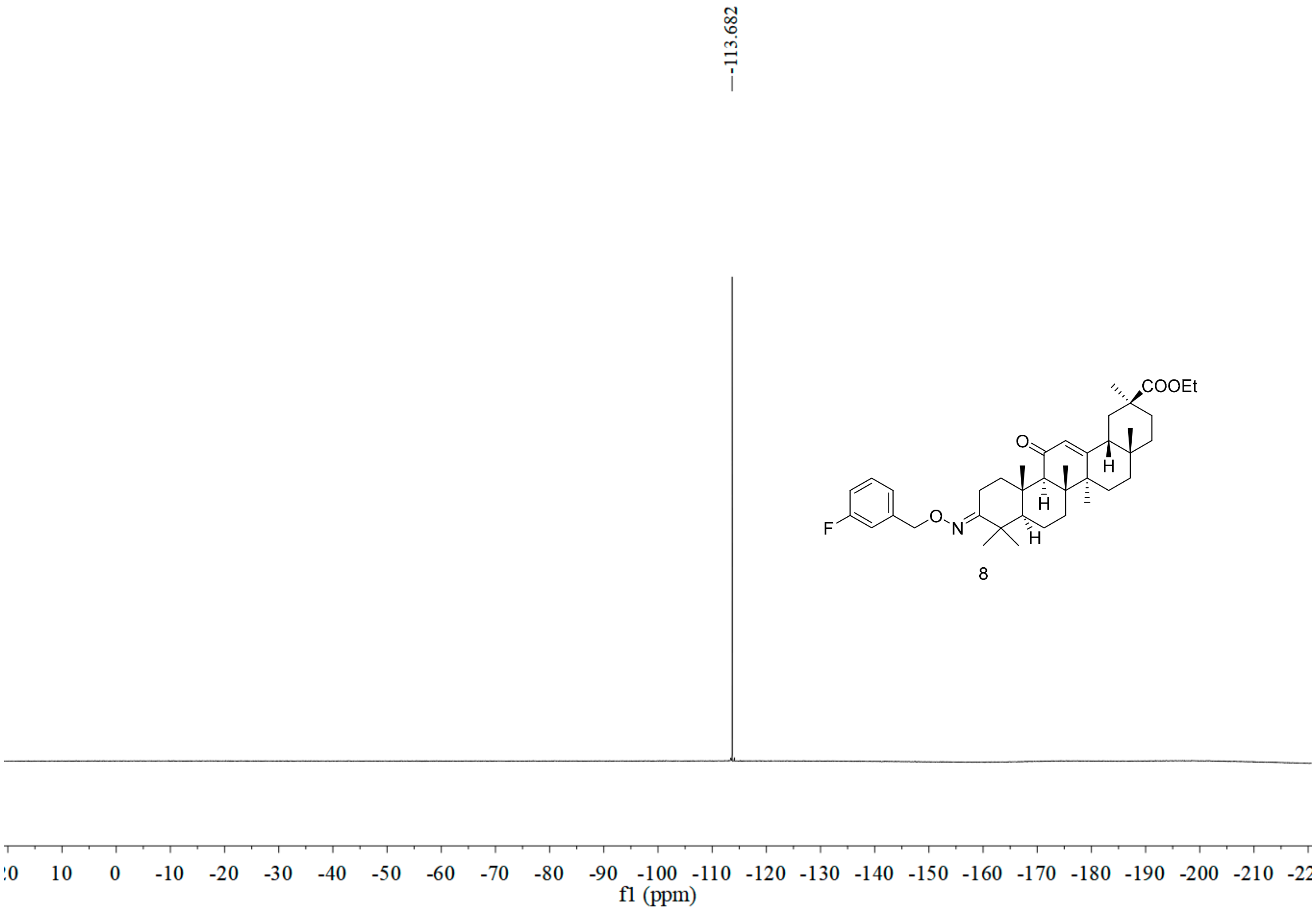

^{19}F NMR of Compound **8** (377 MHz, $CDCl_3$)

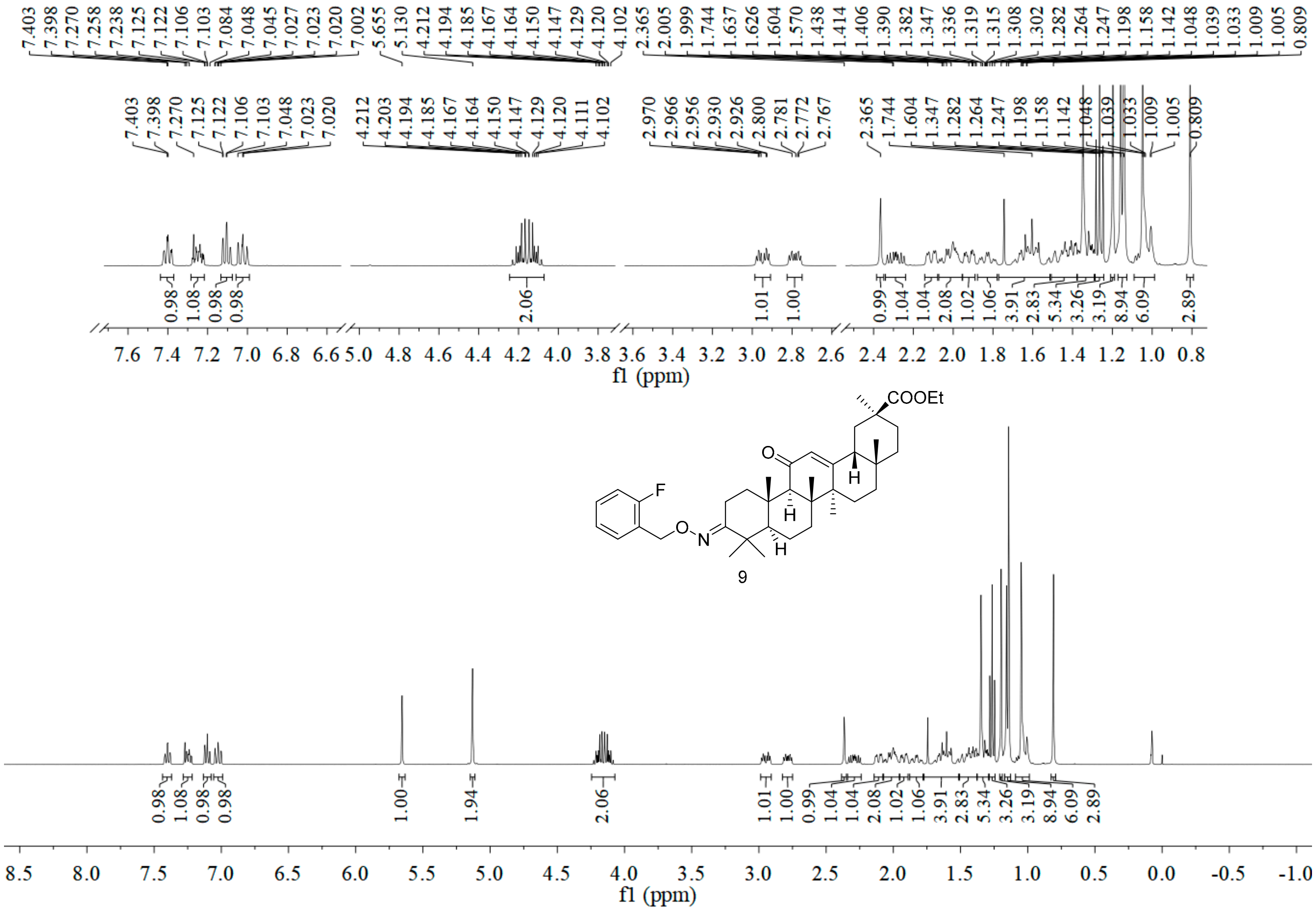

^{1}H NMR of Compound **9** (400 MHz, $CDCl_3$)

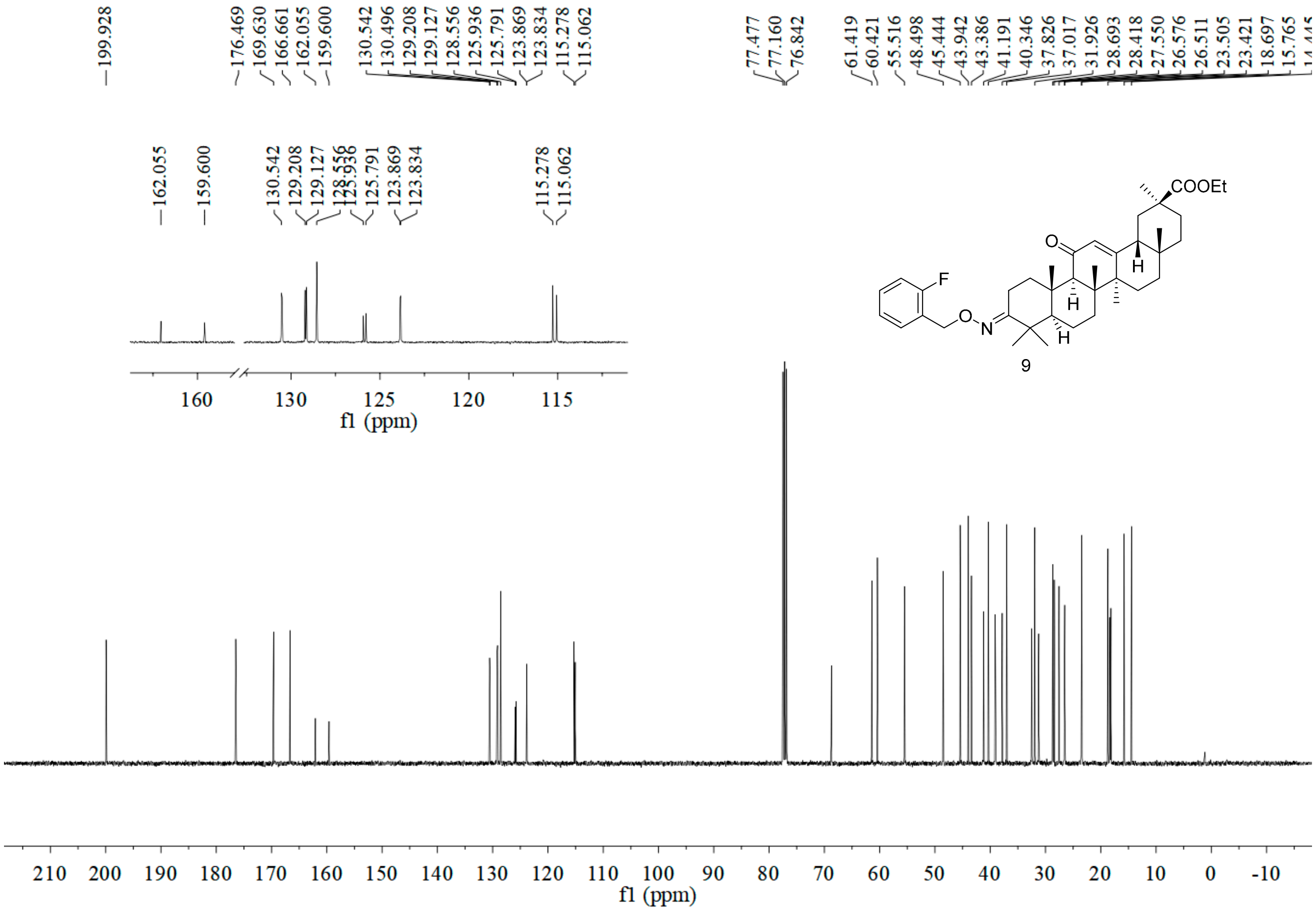

^{13}C NMR of Compound **9** (100 MHz, $CDCl_3$)

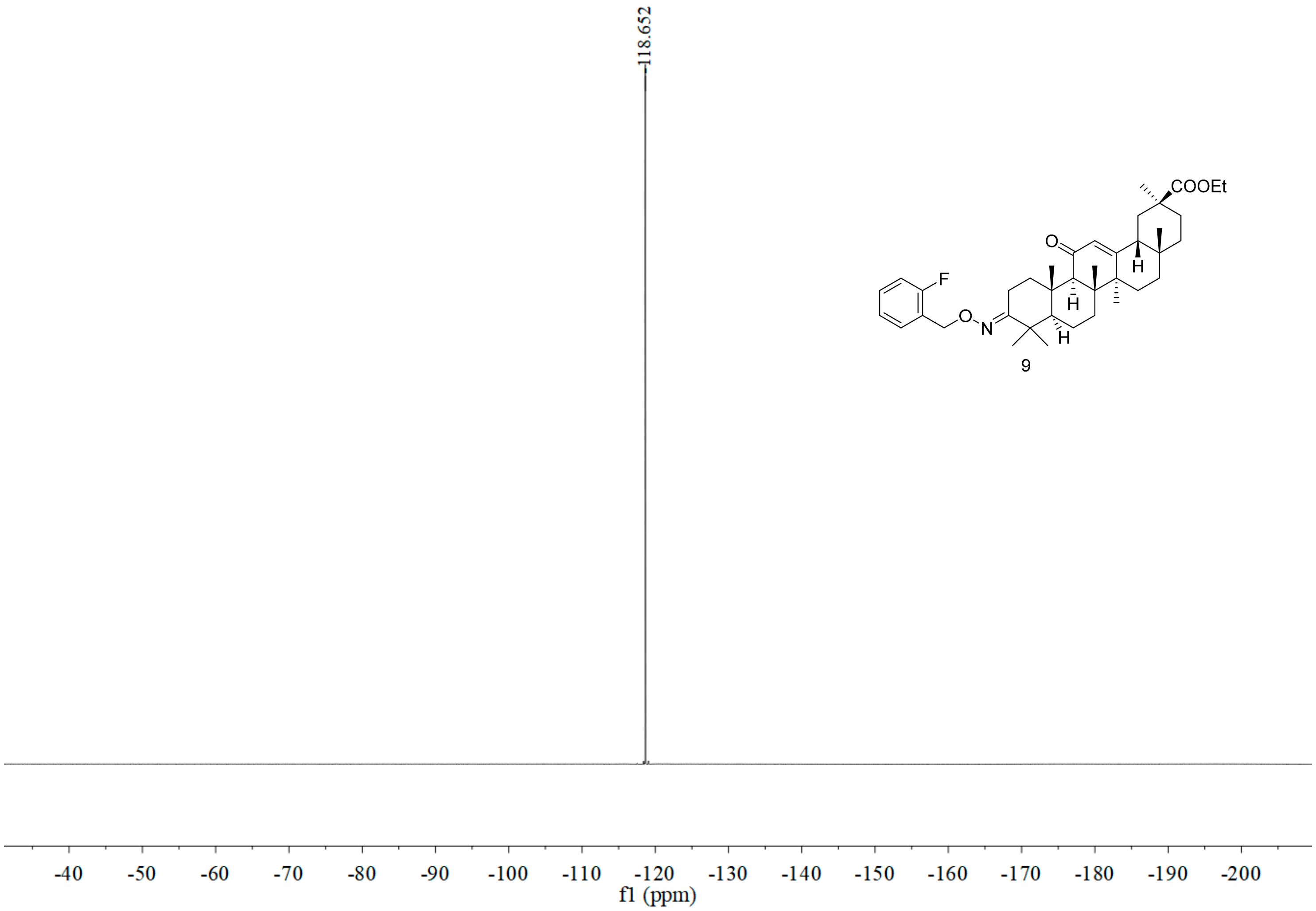

^{19}F NMR of Compound **9** (377 MHz, $CDCl_3$)

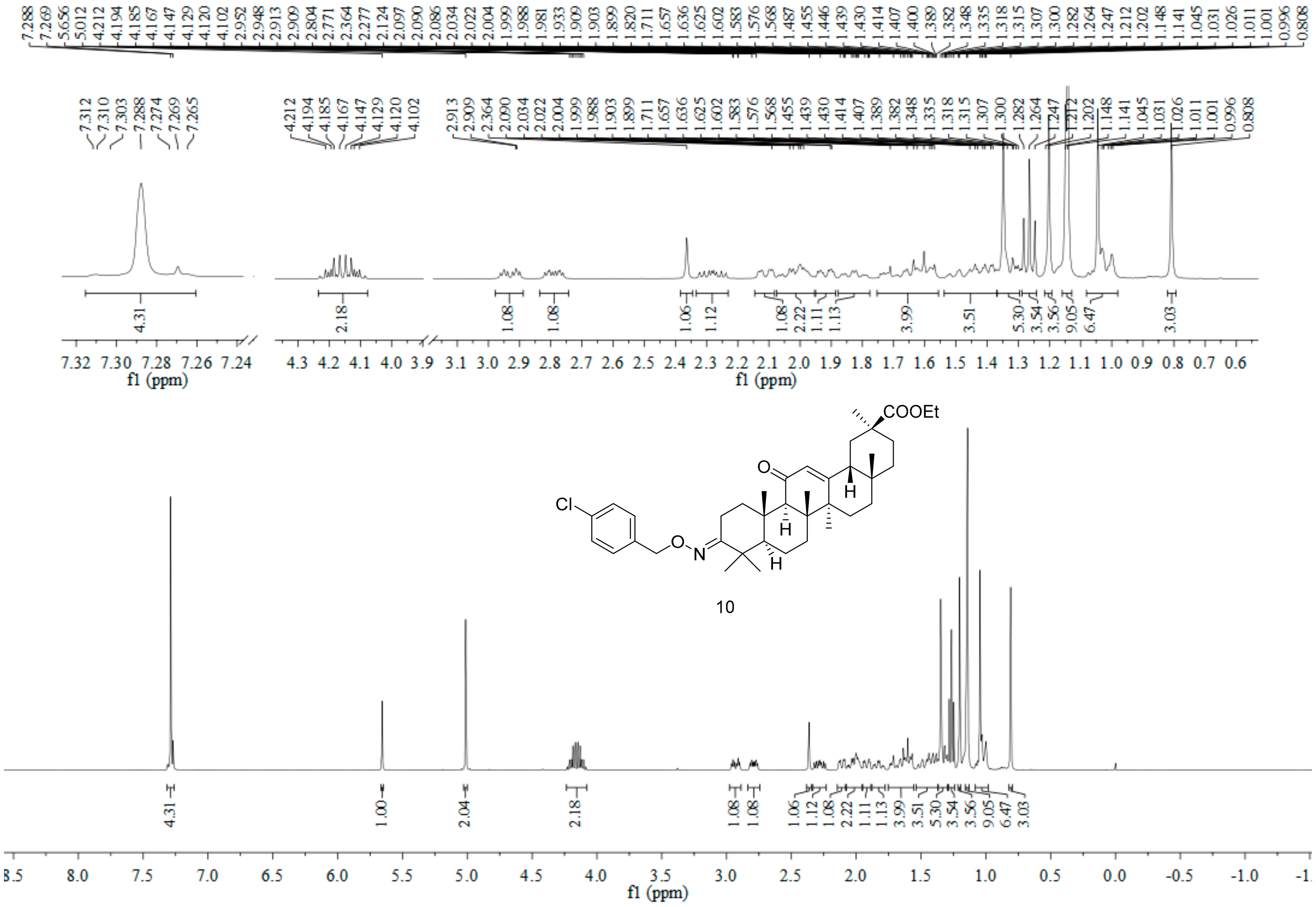

10

^{1}H NMR of Compound **10** (400 MHz, $CDCl_3$)

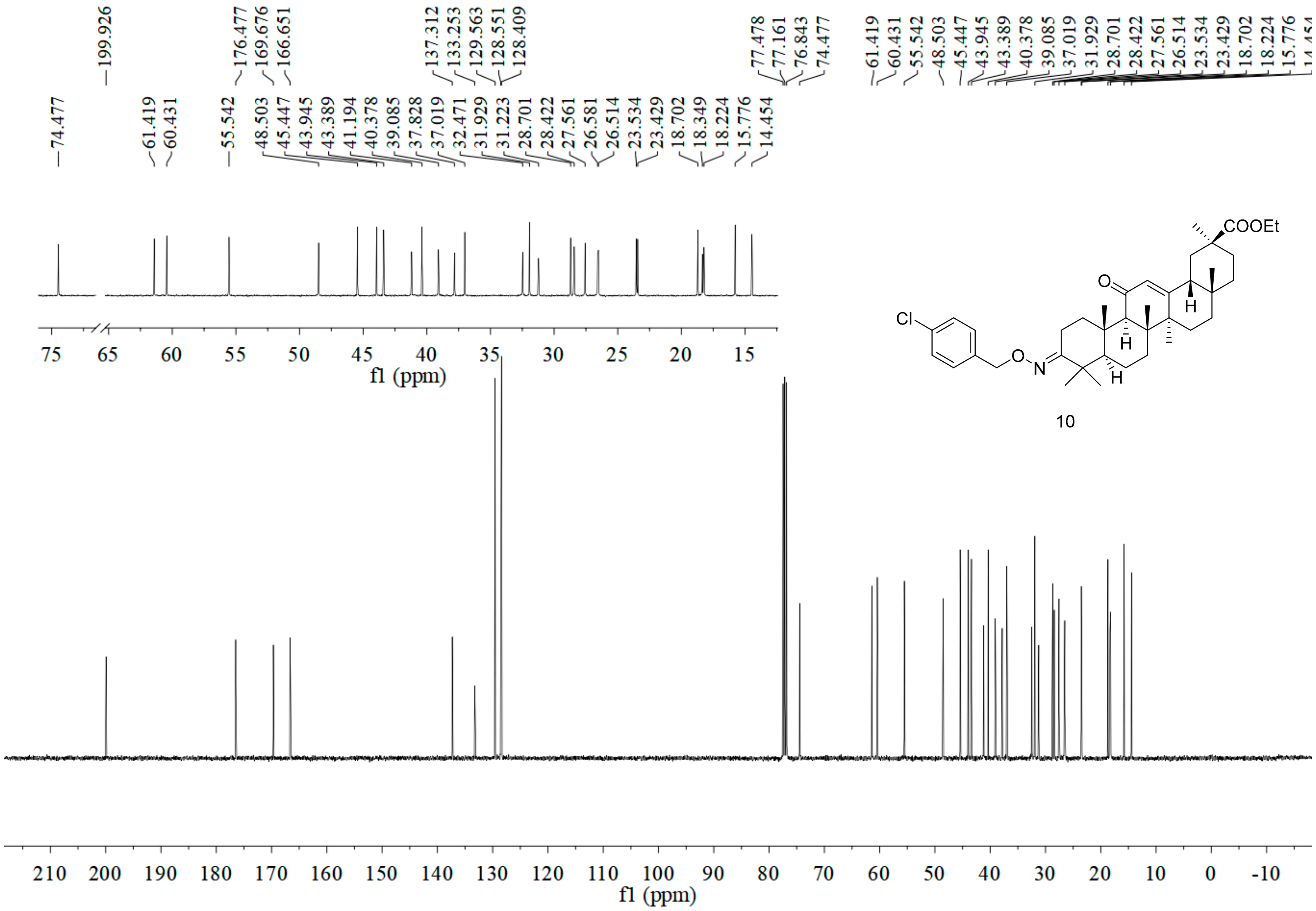

10

^{13}C NMR of Compound **10** (100 MHz, $CDCl_3$)

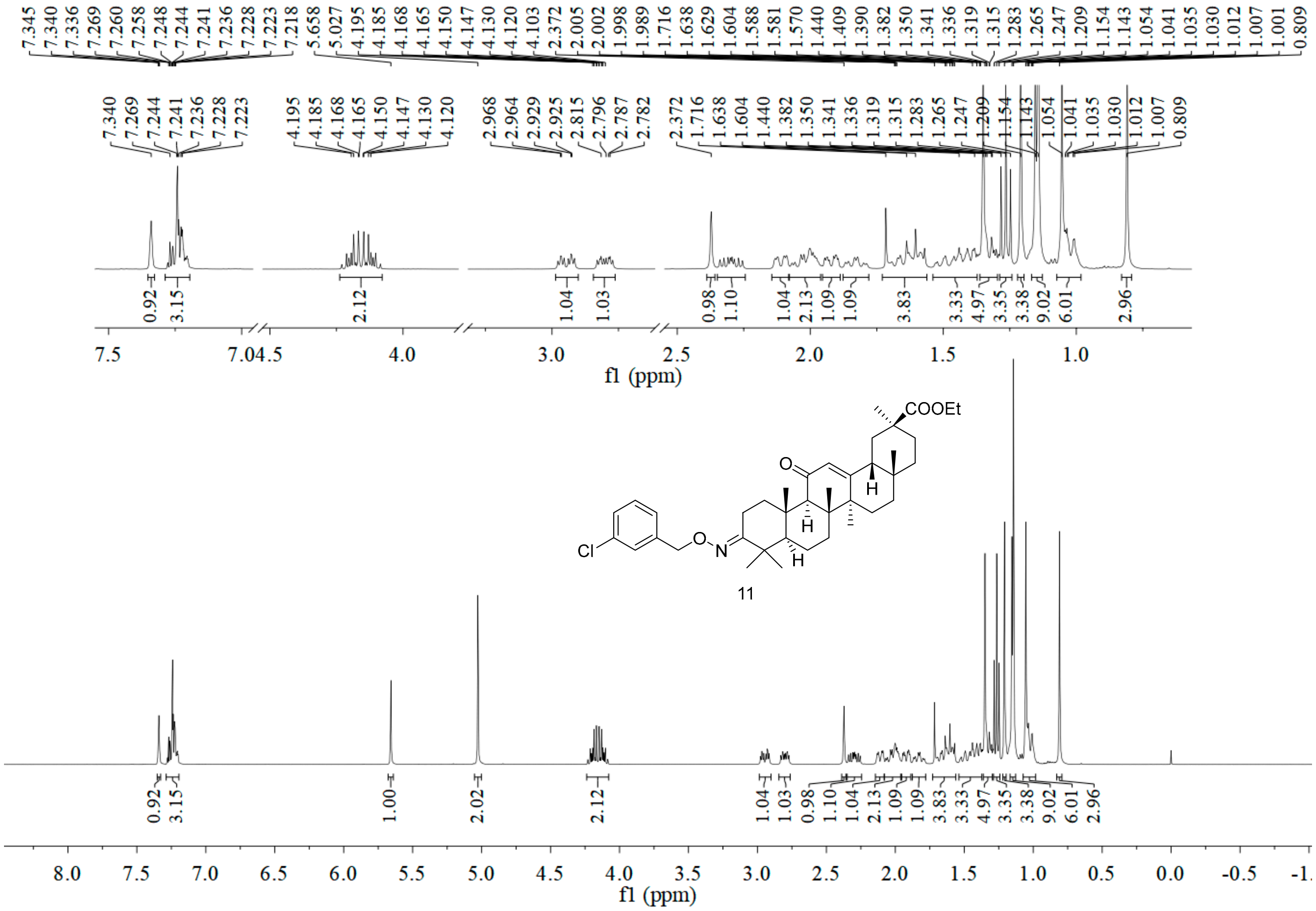

^{1}H NMR of Compound **11** (400 MHz, $CDCl_3$)

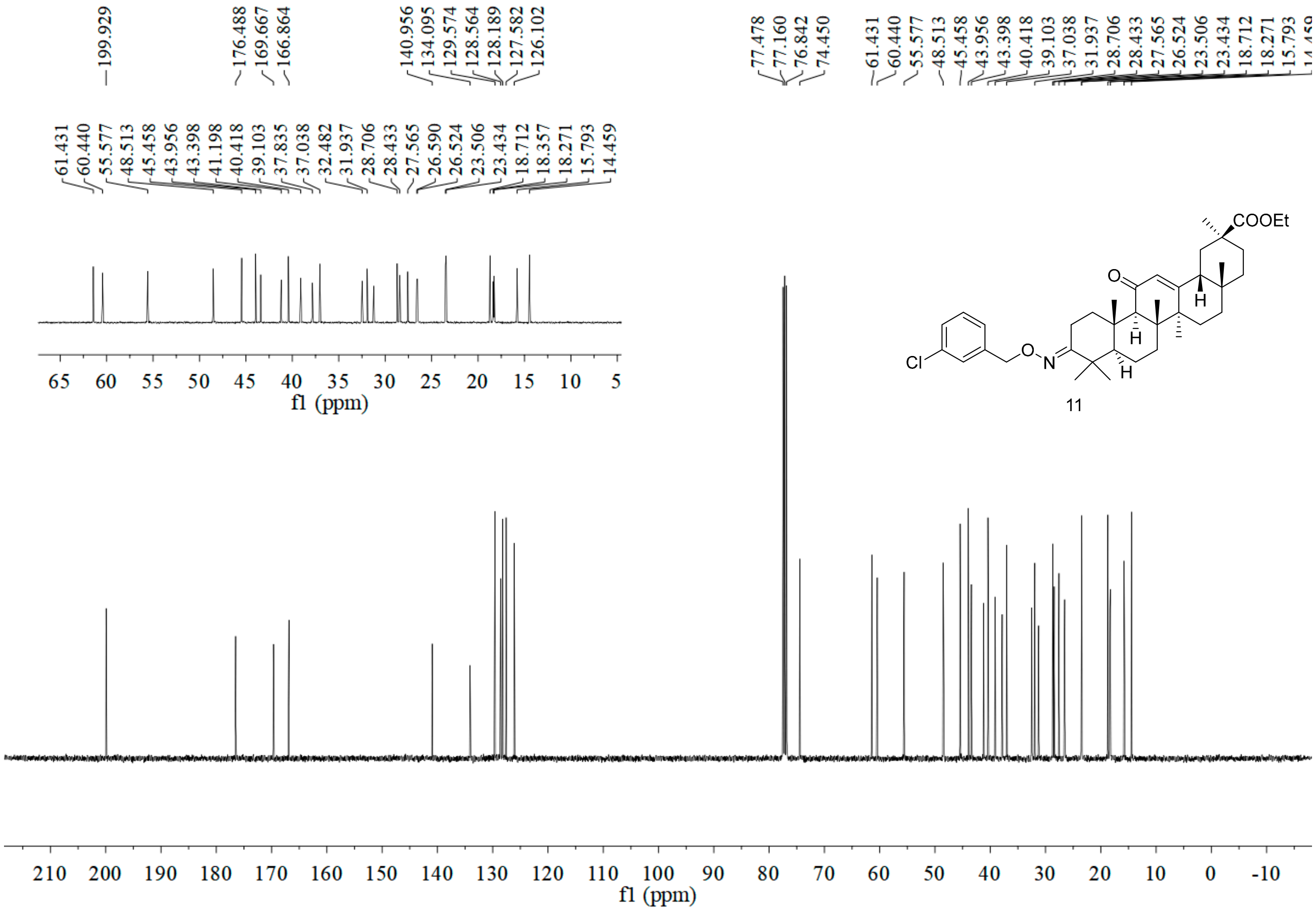

^{13}C NMR of Compound **11** (100 MHz, $CDCl_3$)

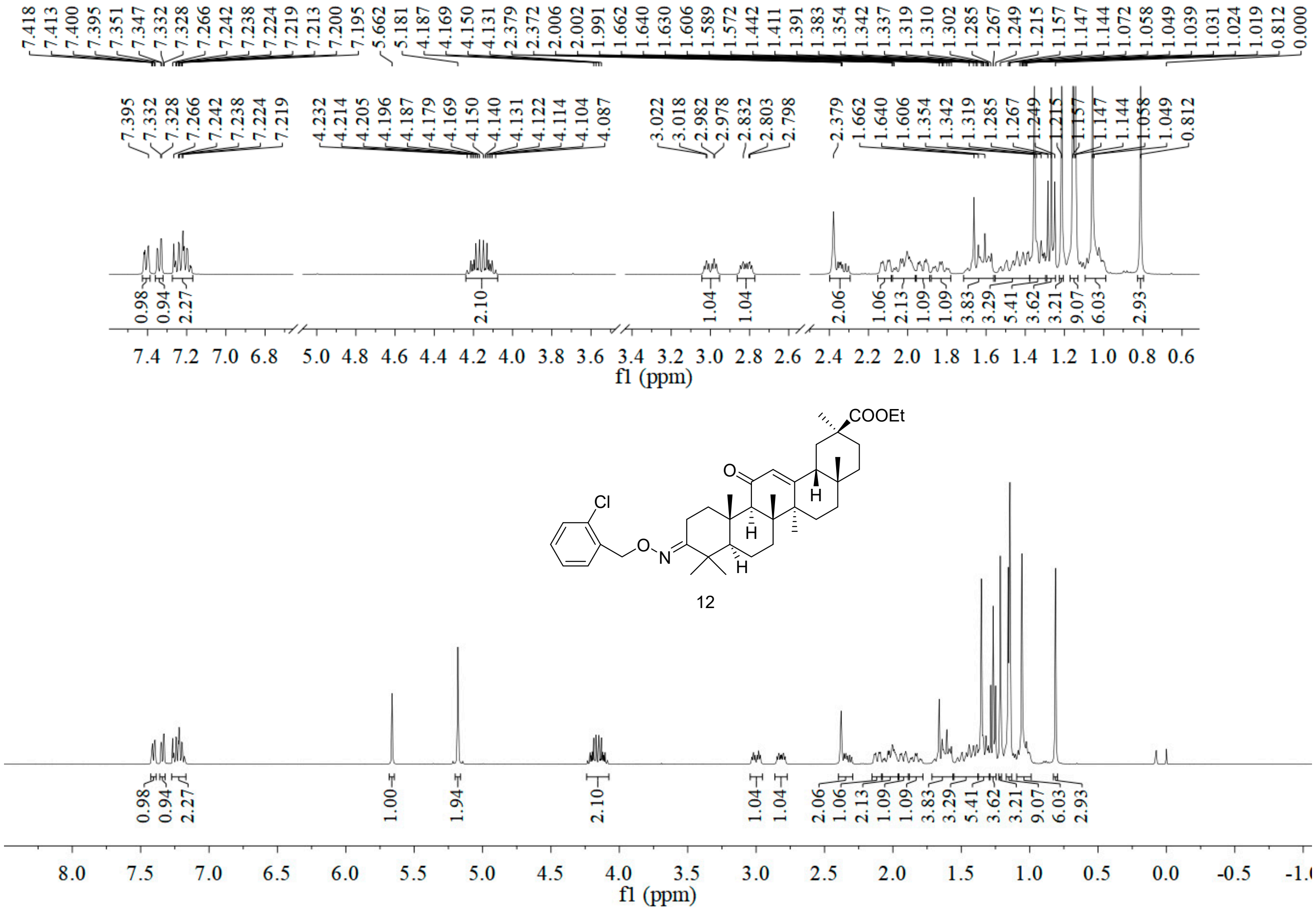

^{1}H NMR of Compound **12** (400 MHz, $CDCl_3$)

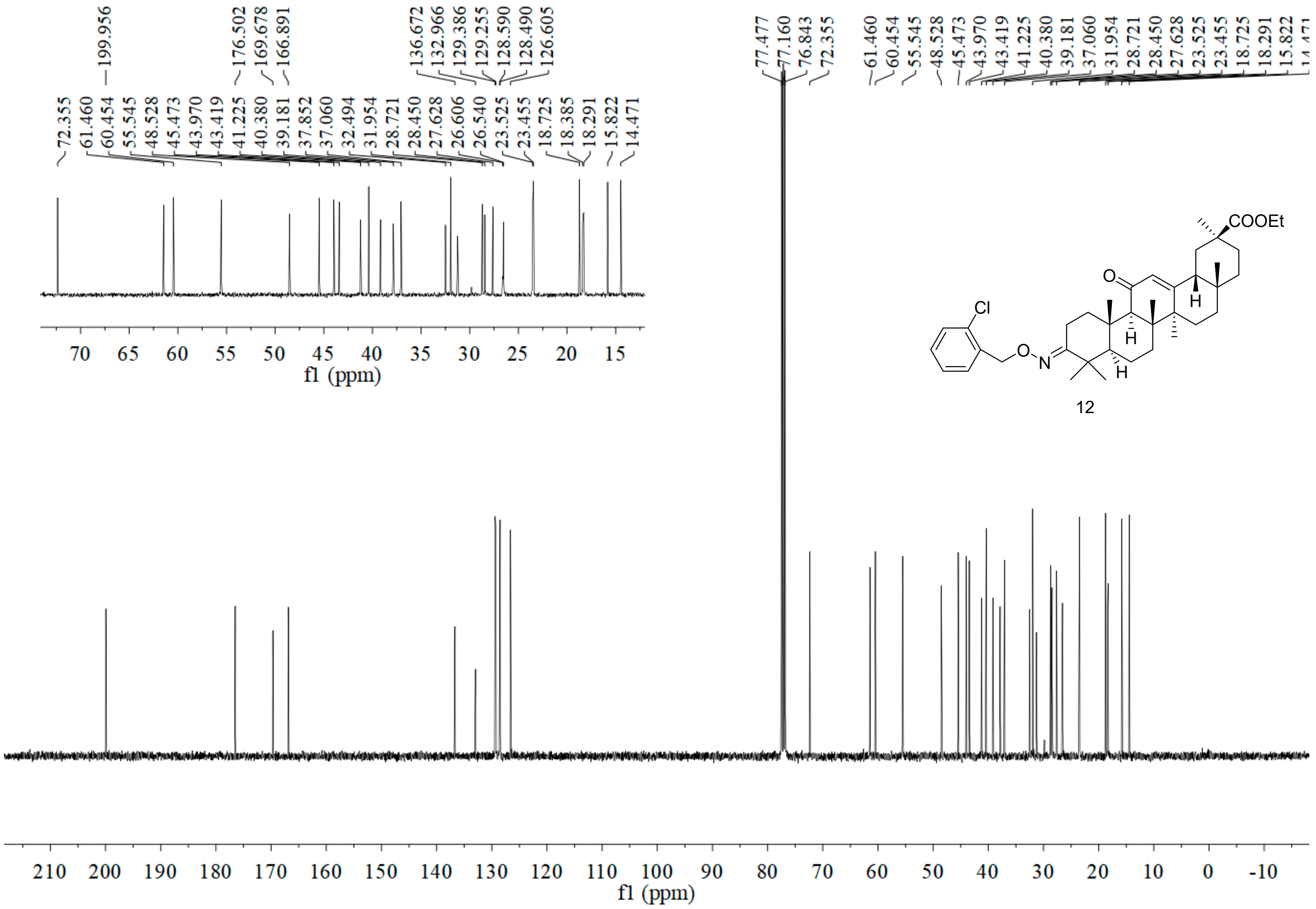

^{13}C NMR of Compound **12** (100 MHz, $CDCl_3$)

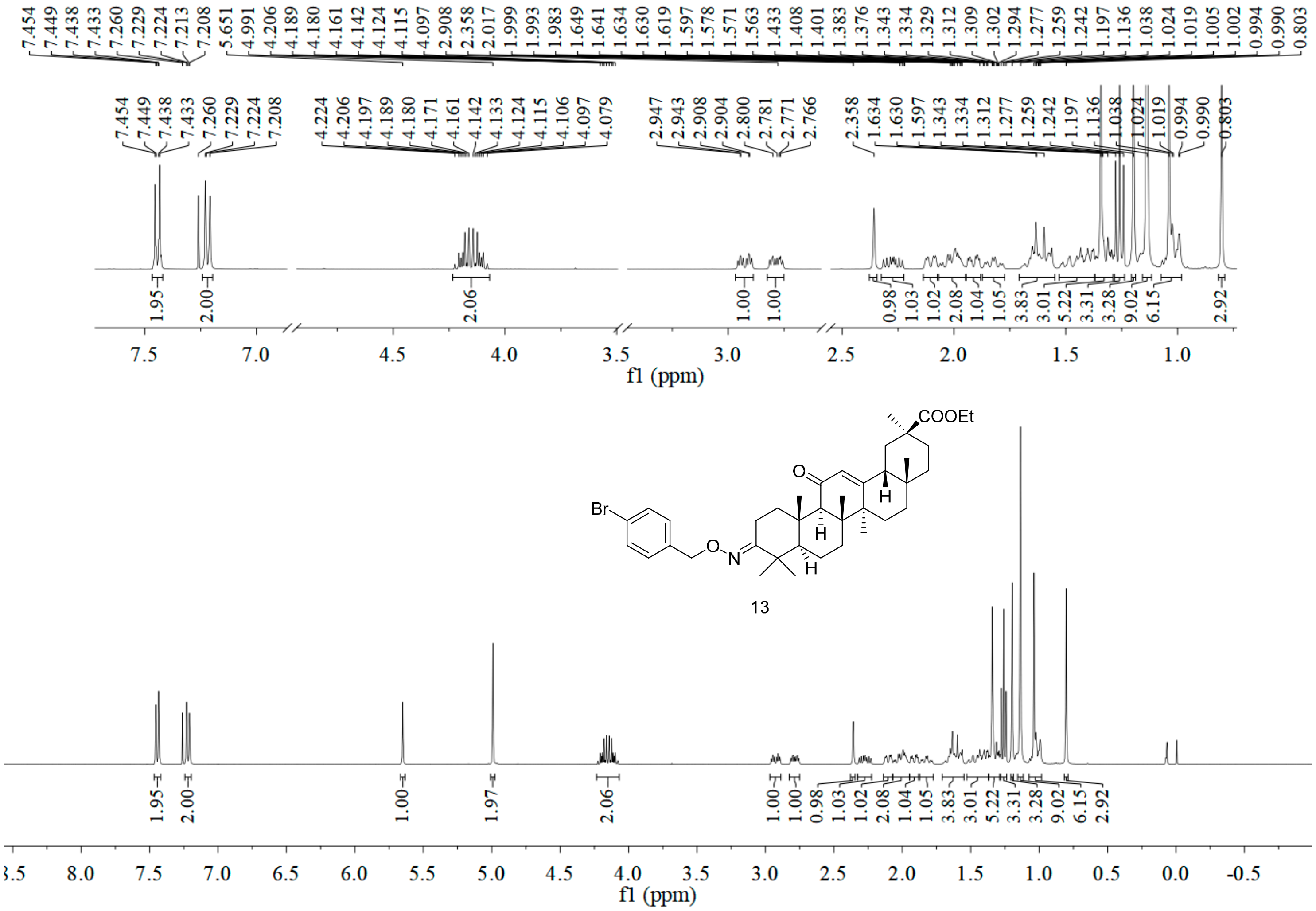

^{1}H NMR of Compound **13** (400 MHz, $CDCl_3$)

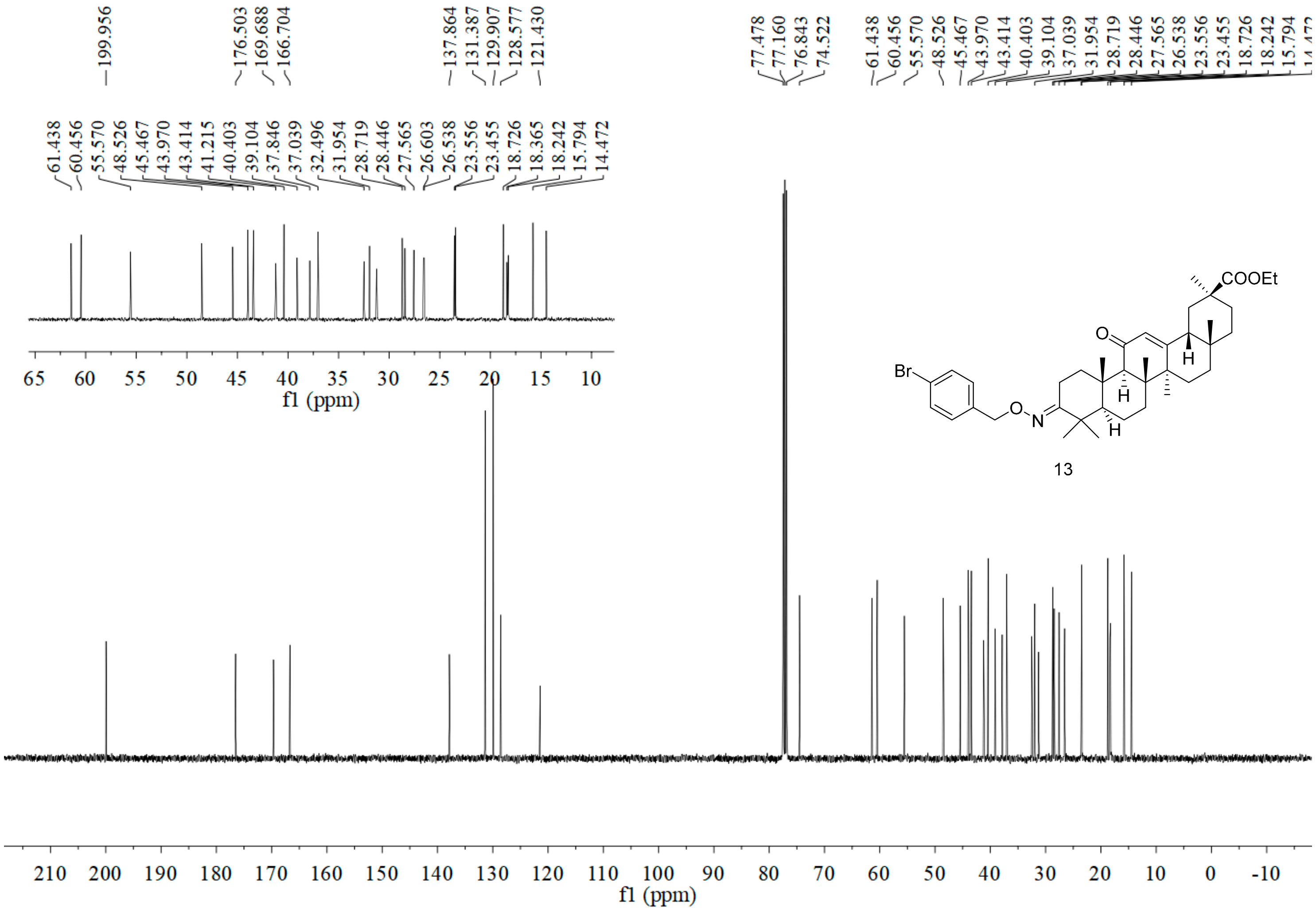

^{13}C NMR of Compound **13** (100 MHz, $CDCl_3$)

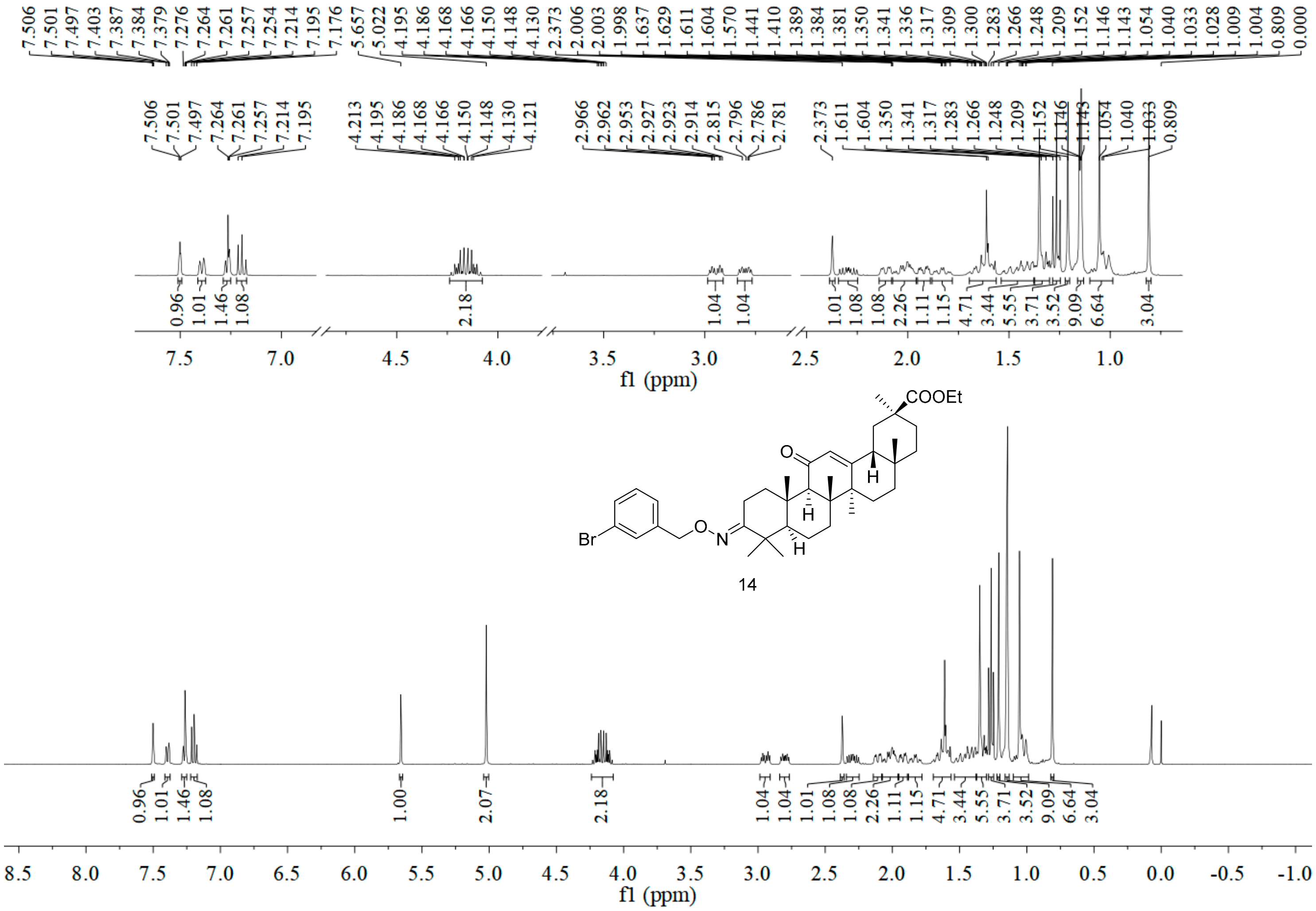

^{1}H NMR of Compound **14** (400 MHz, $CDCl_3$)

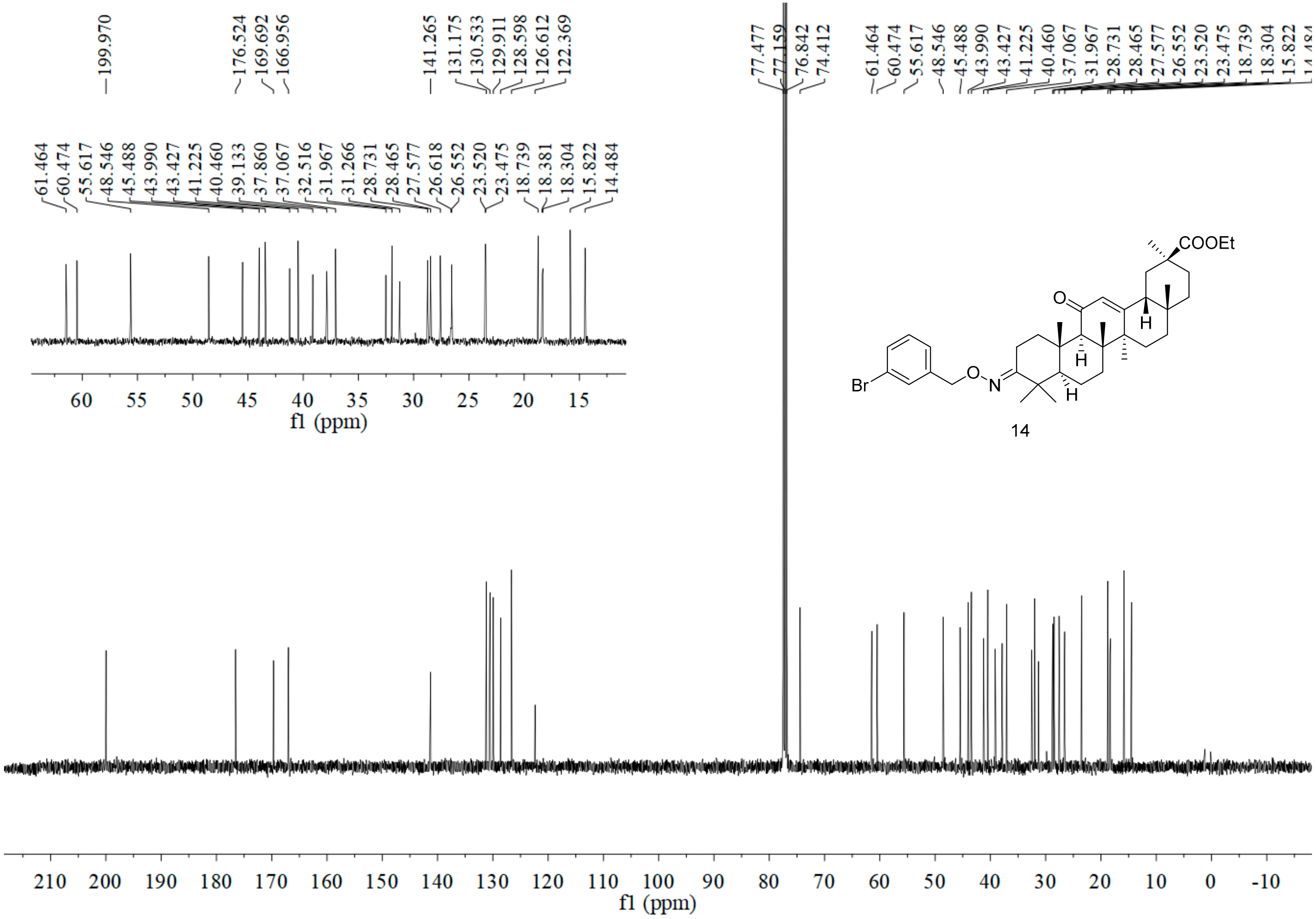

^{13}C NMR of Compound **14** (100 MHz, $CDCl_3$)

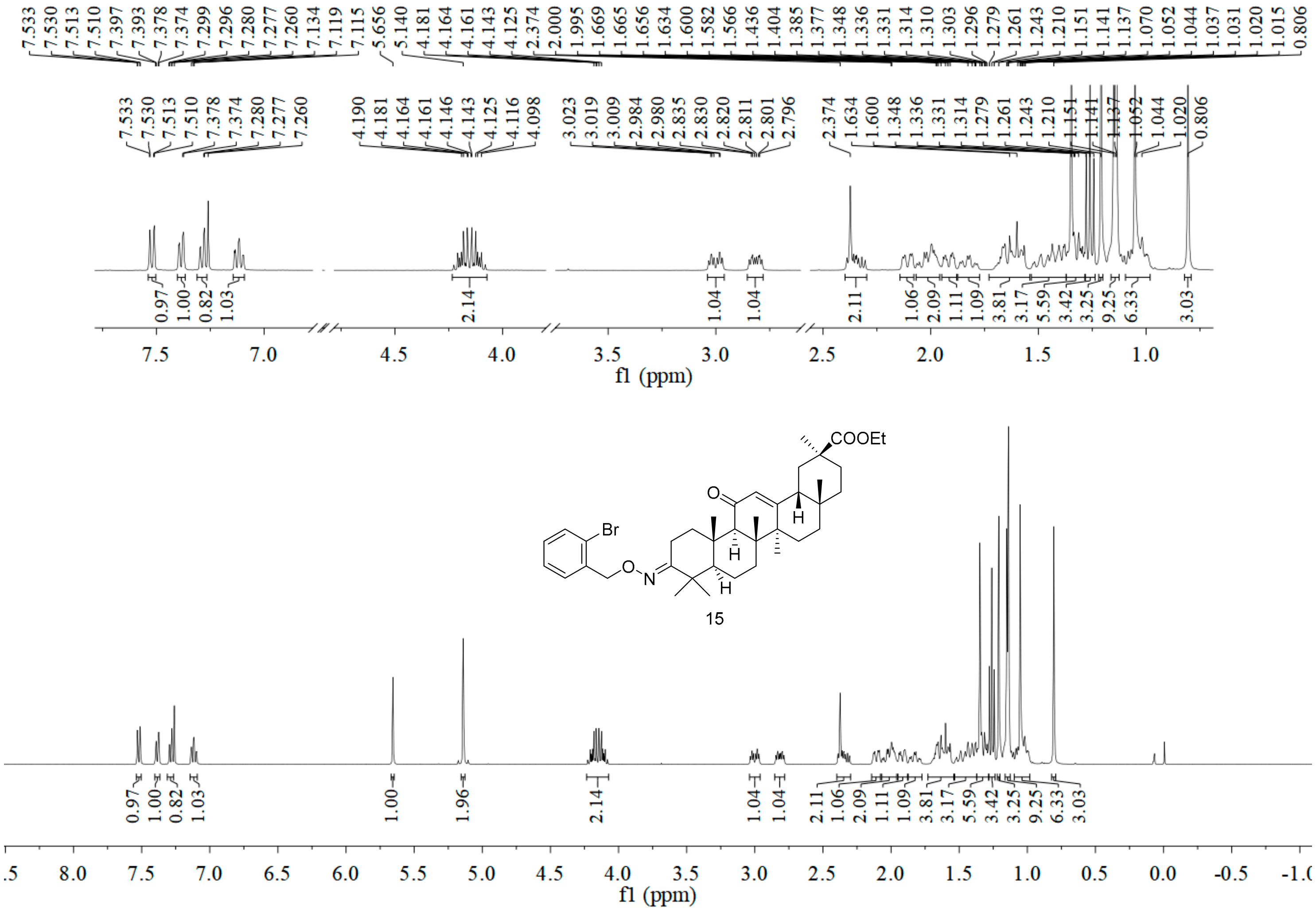

^{1}H NMR of Compound **15** (400 MHz, $CDCl_3$)

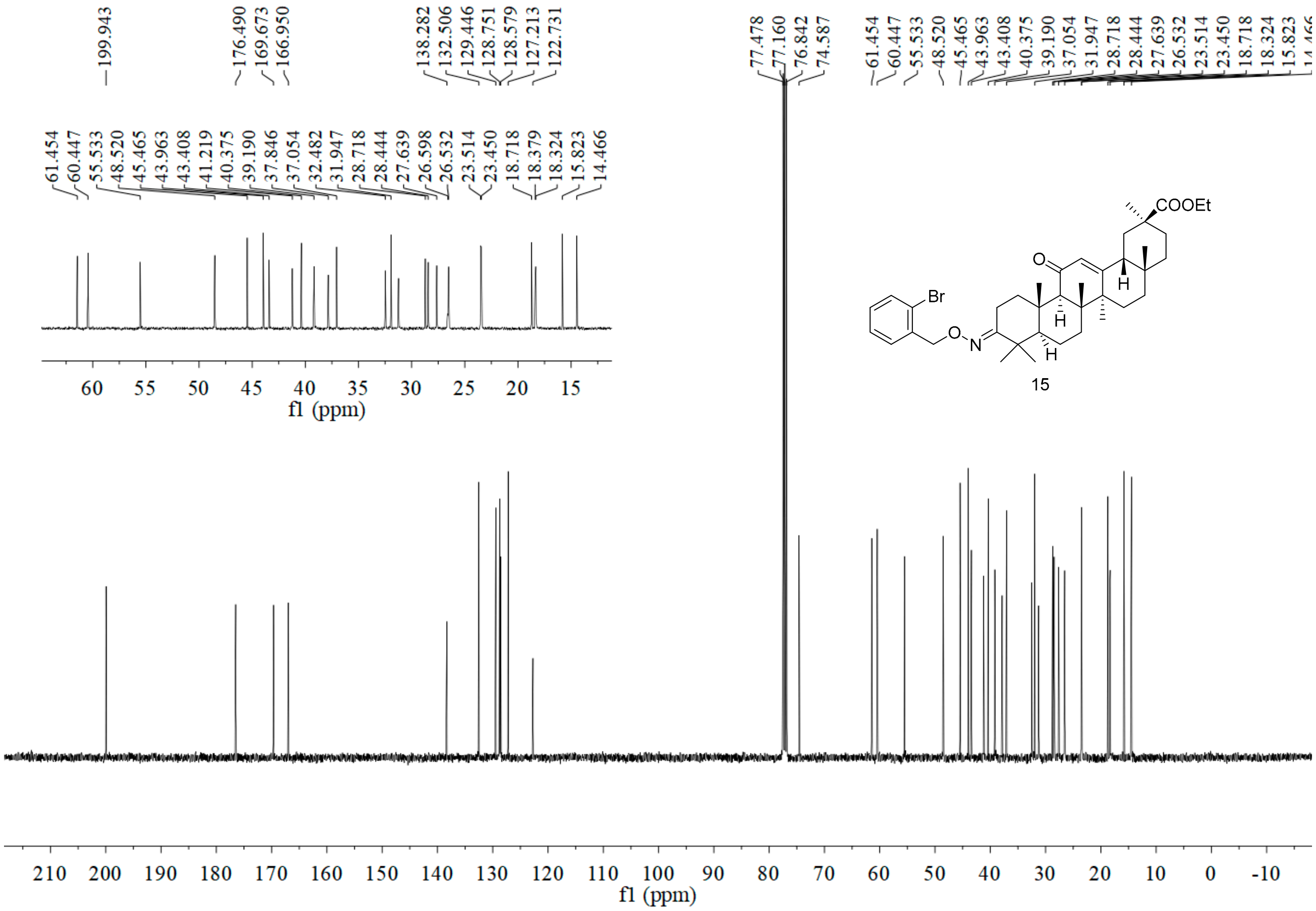

^{13}C NMR of Compound **15** (100 MHz, $CDCl_3$)

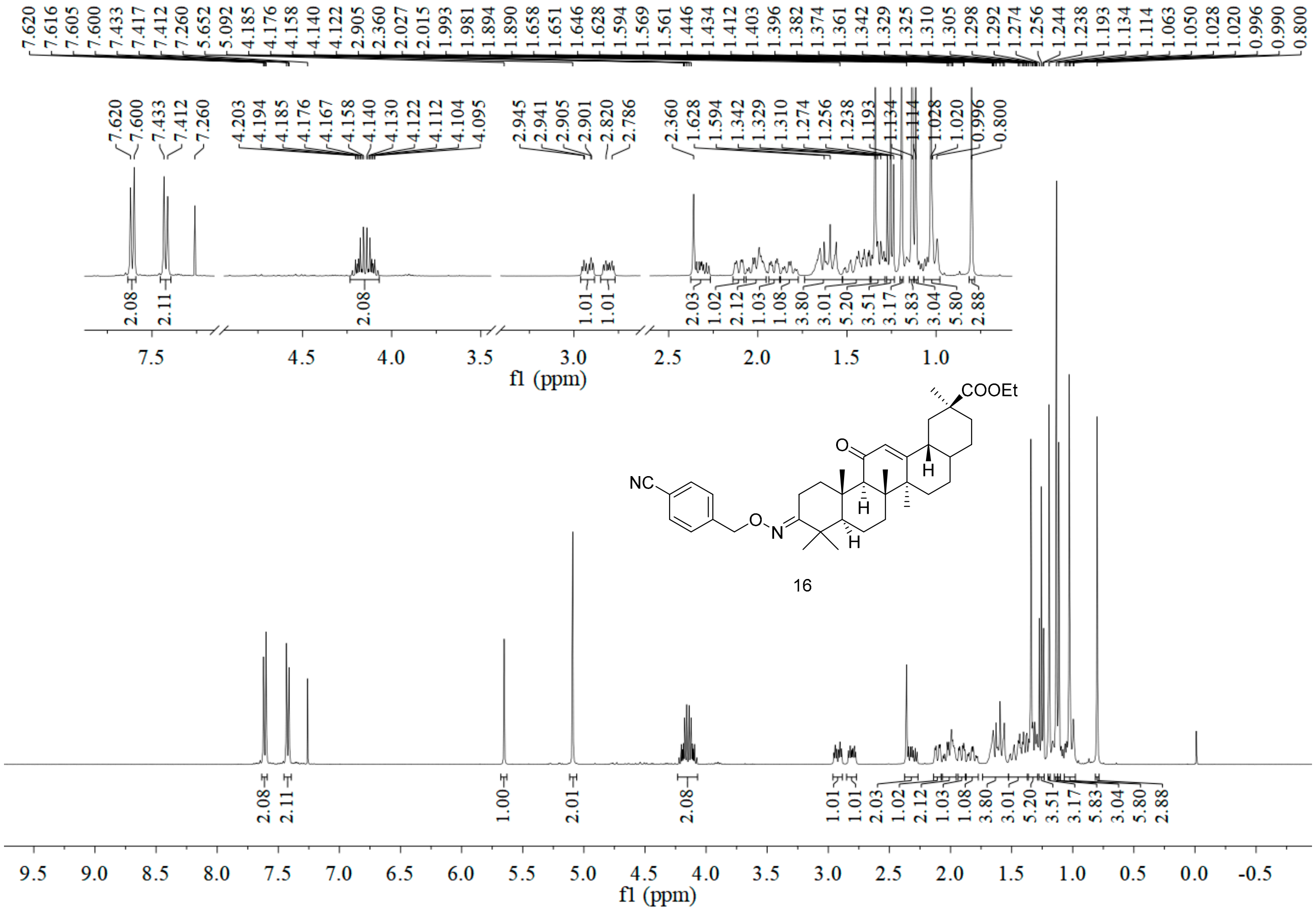

^{1}H NMR of Compound **16** (400 MHz, $CDCl_3$)

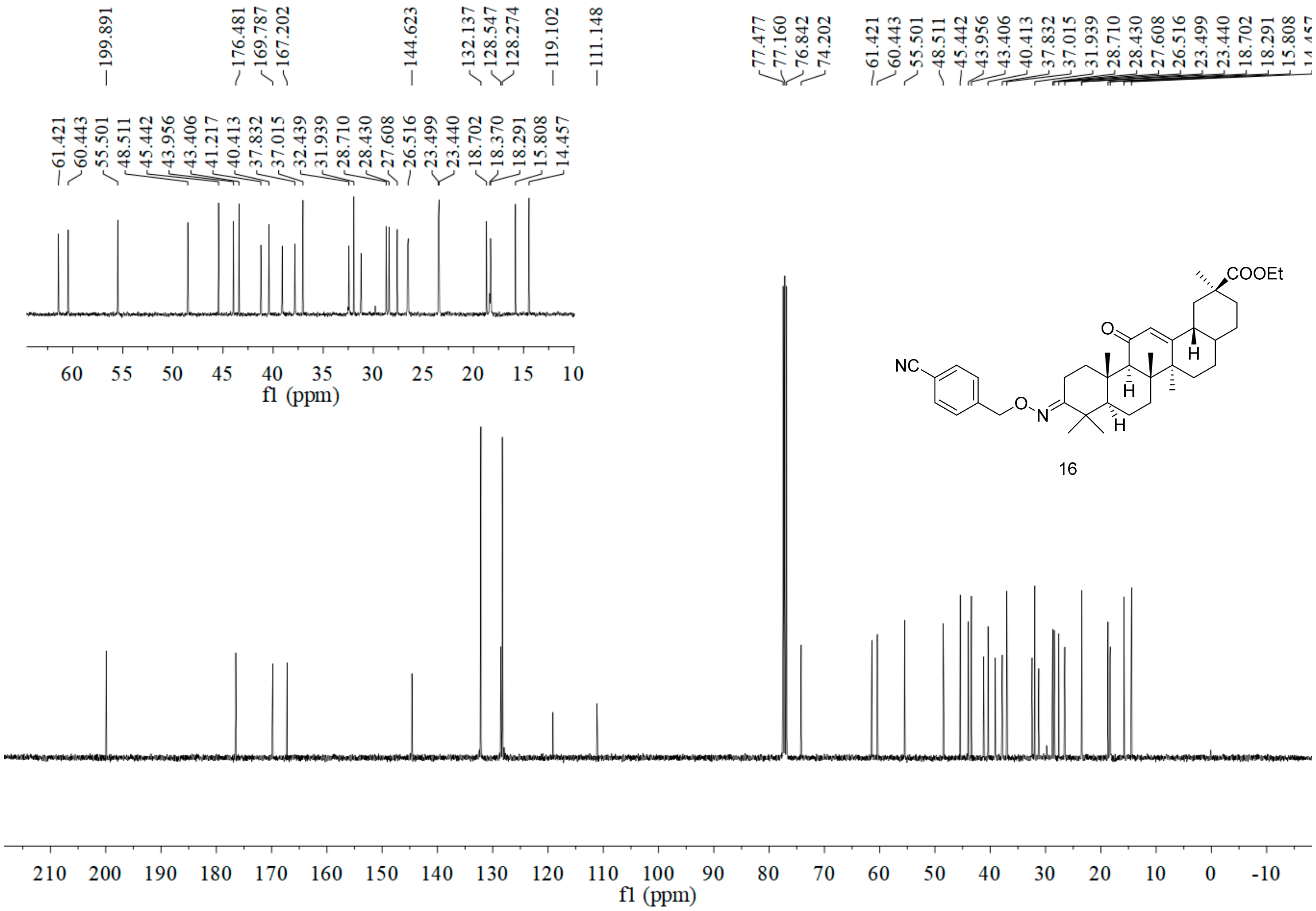

^{13}C NMR of Compound **16** (100 MHz, $CDCl_3$)

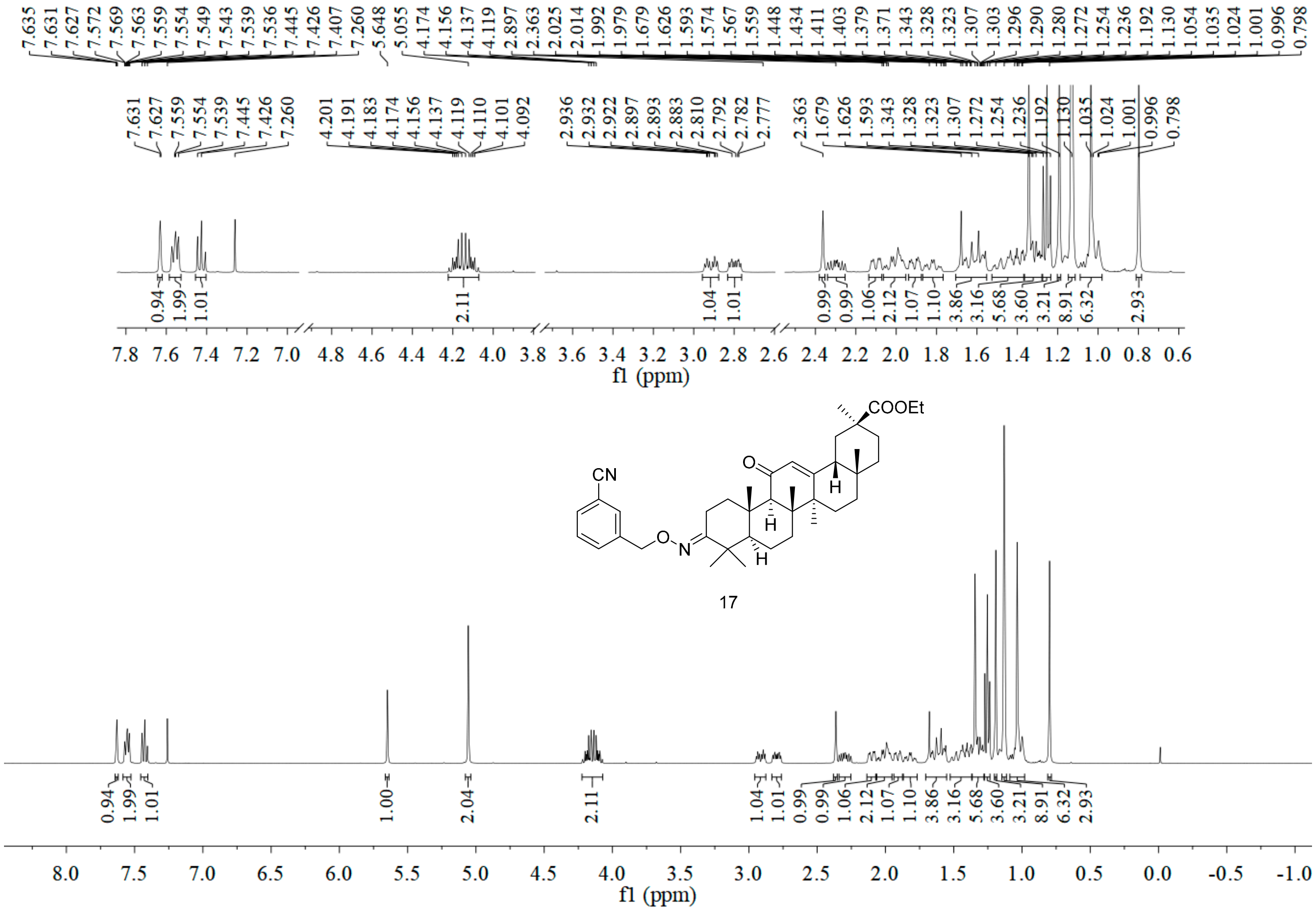

17

^{1}H NMR of Compound **17** (400 MHz, $CDCl_3$)

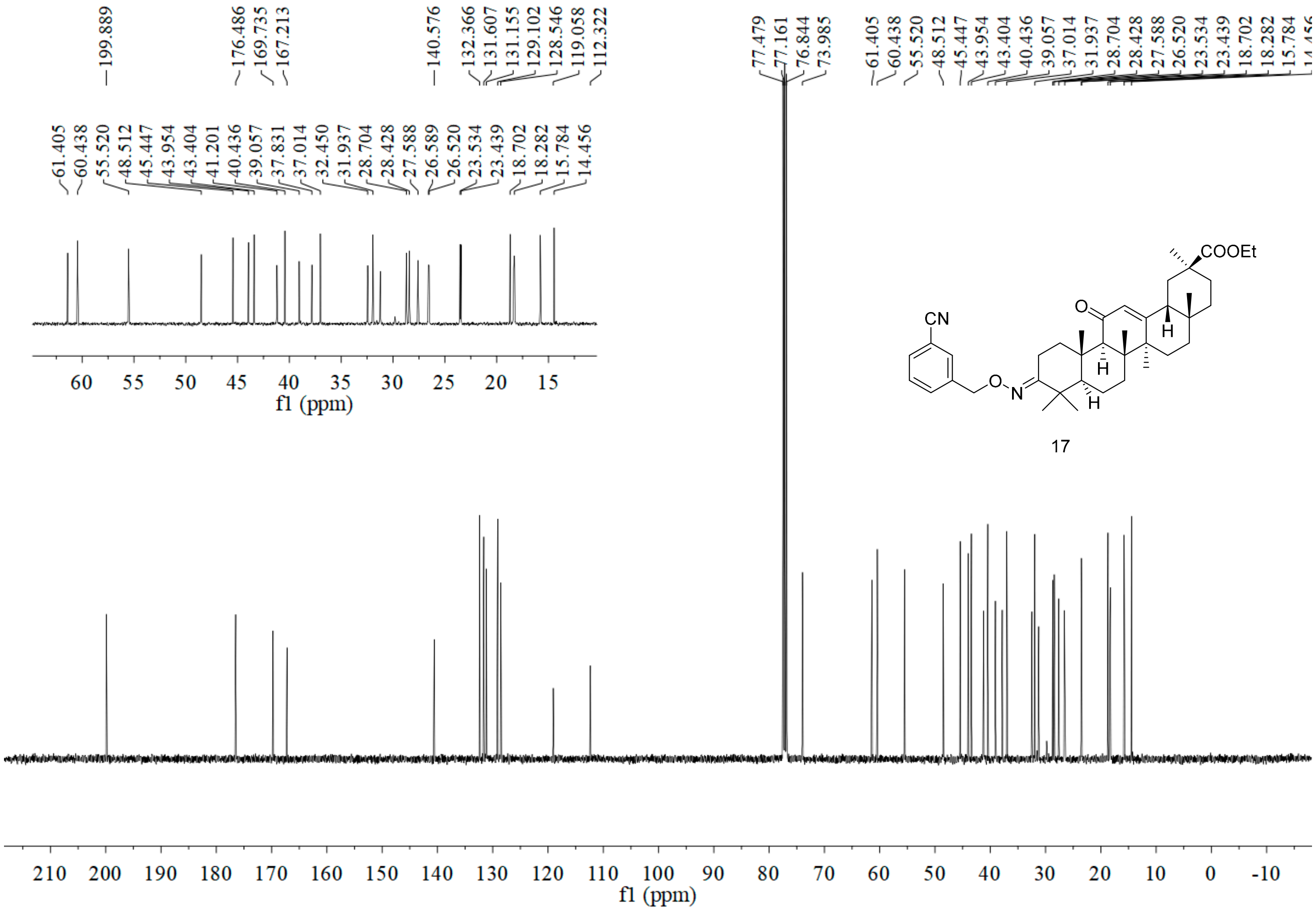

^{13}C NMR of Compound **17** (100 MHz, $CDCl_3$)

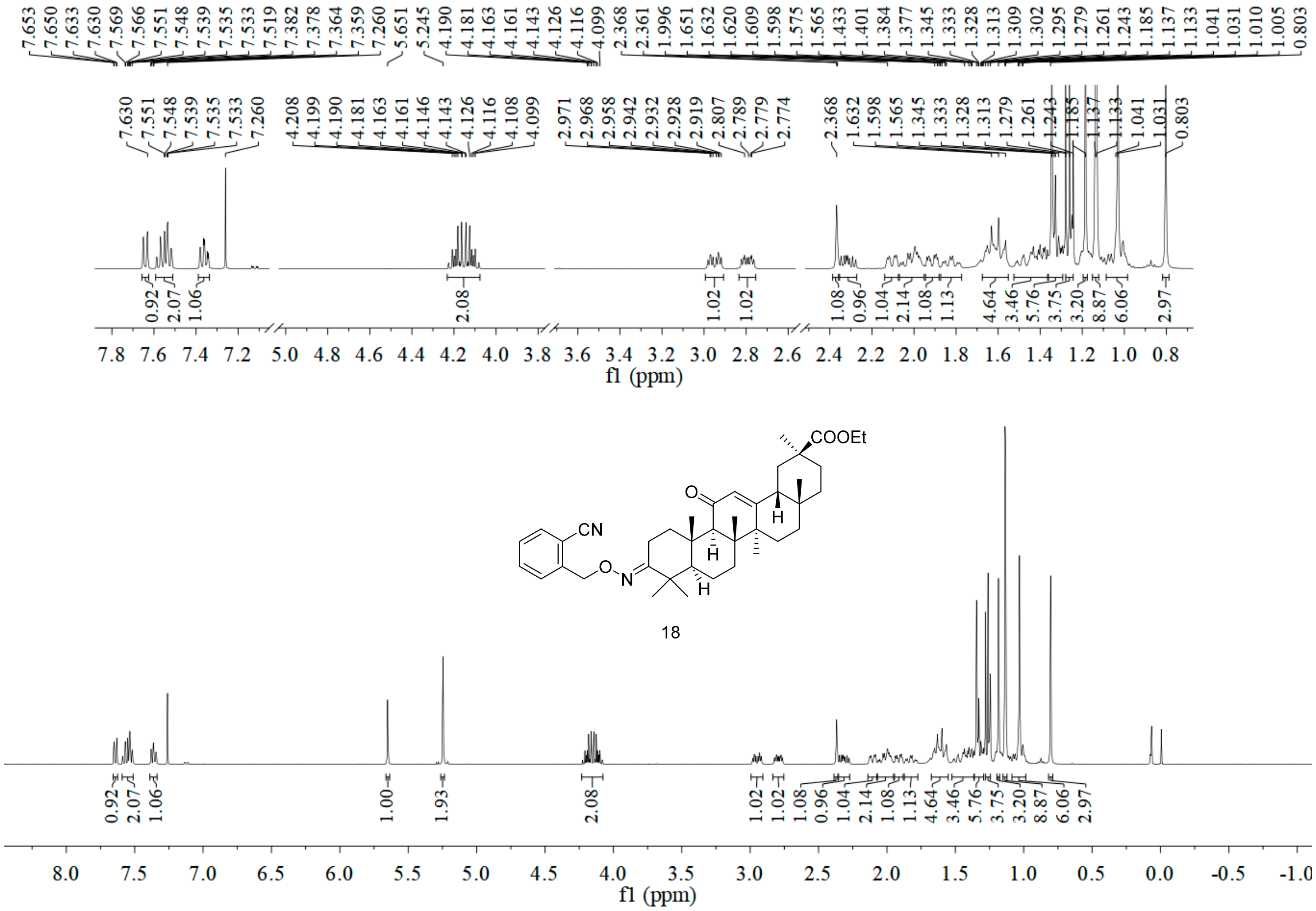

18

^{1}H NMR of Compound **18** (400 MHz, $CDCl_3$)

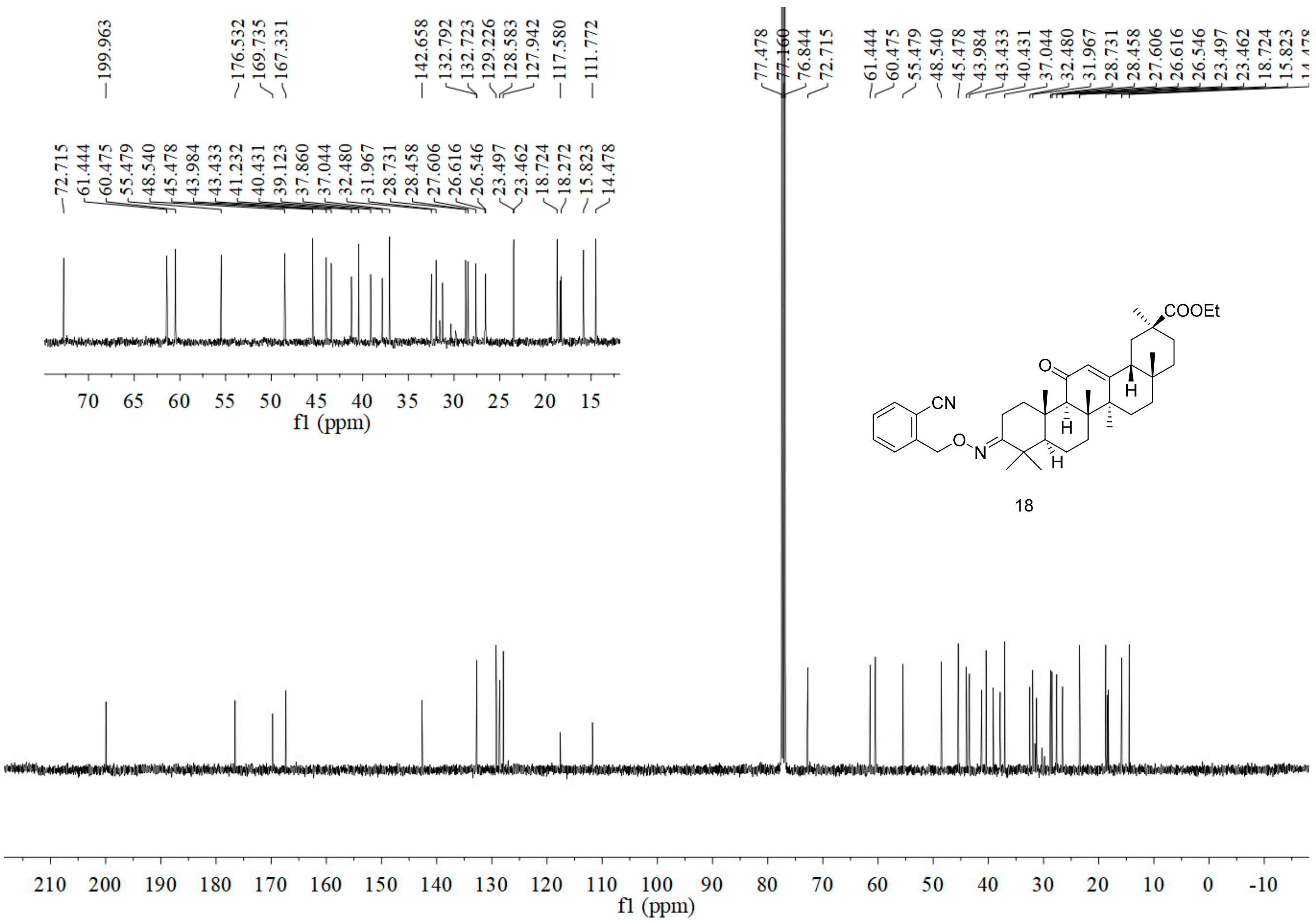

^{13}C NMR of Compound **18** (100 MHz, $CDCl_3$)

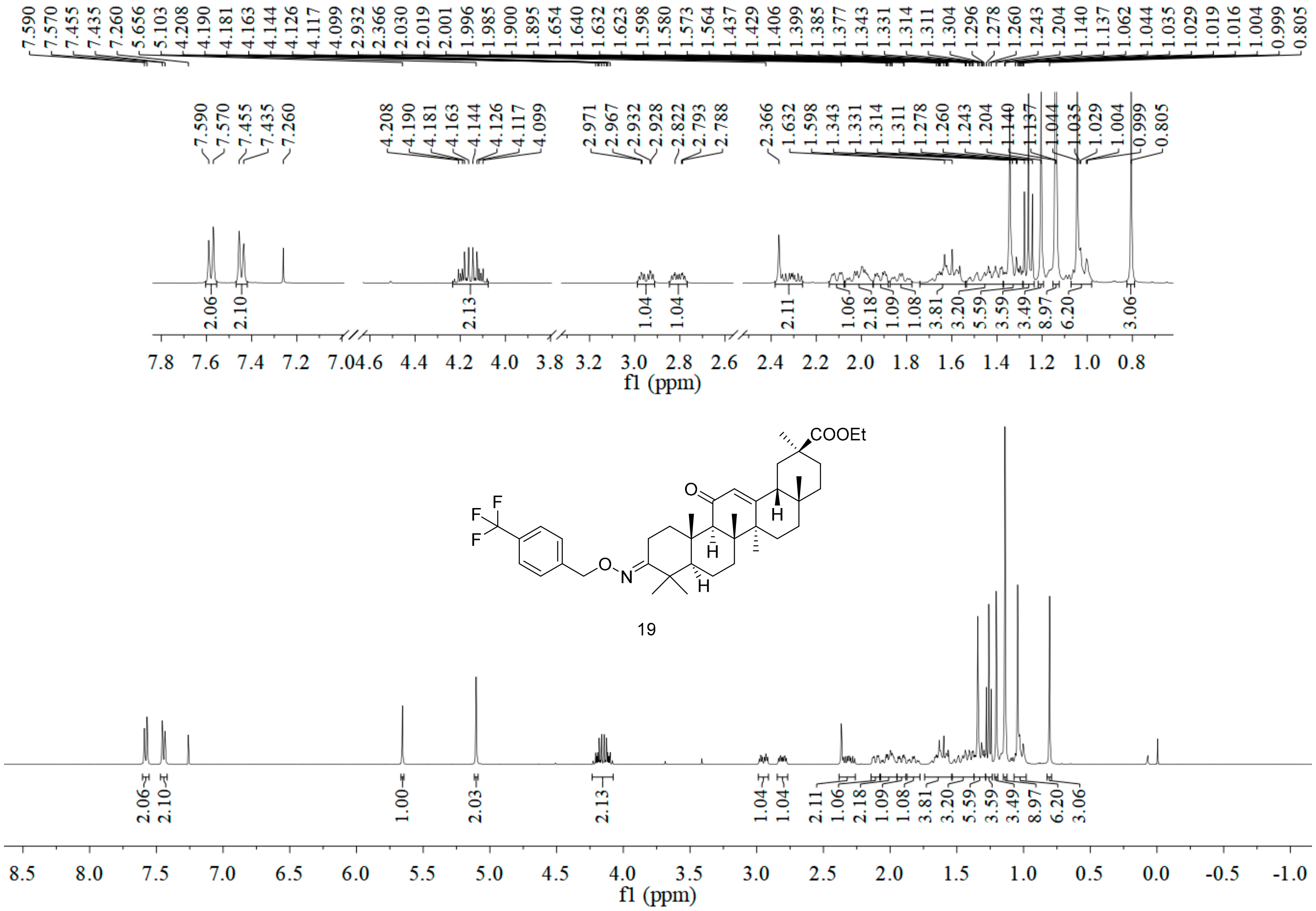

19

^{1}H NMR of Compound **19** (400 MHz, $CDCl_3$)

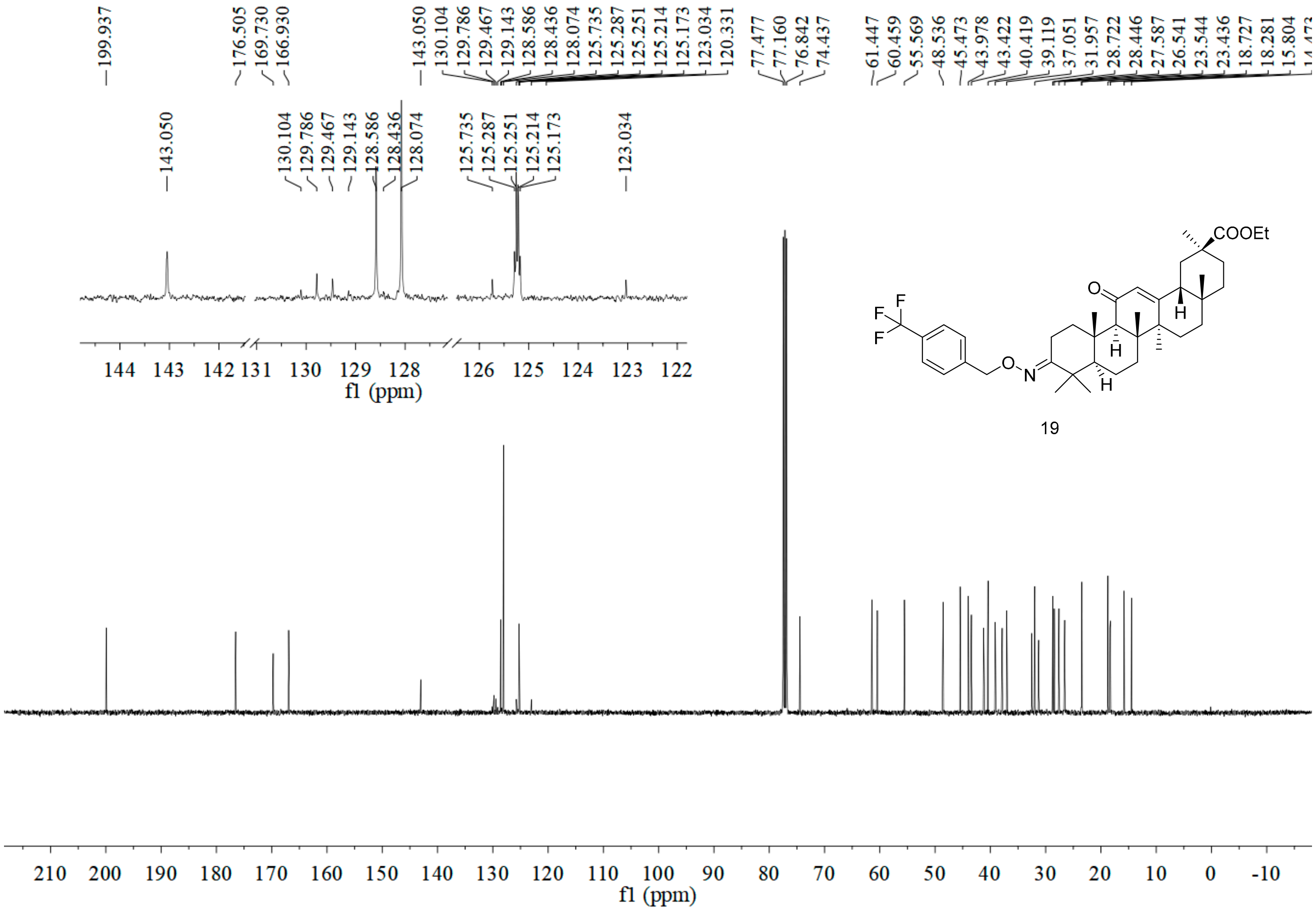

19

^{13}C NMR of Compound **19** (100 MHz, $CDCl_3$)

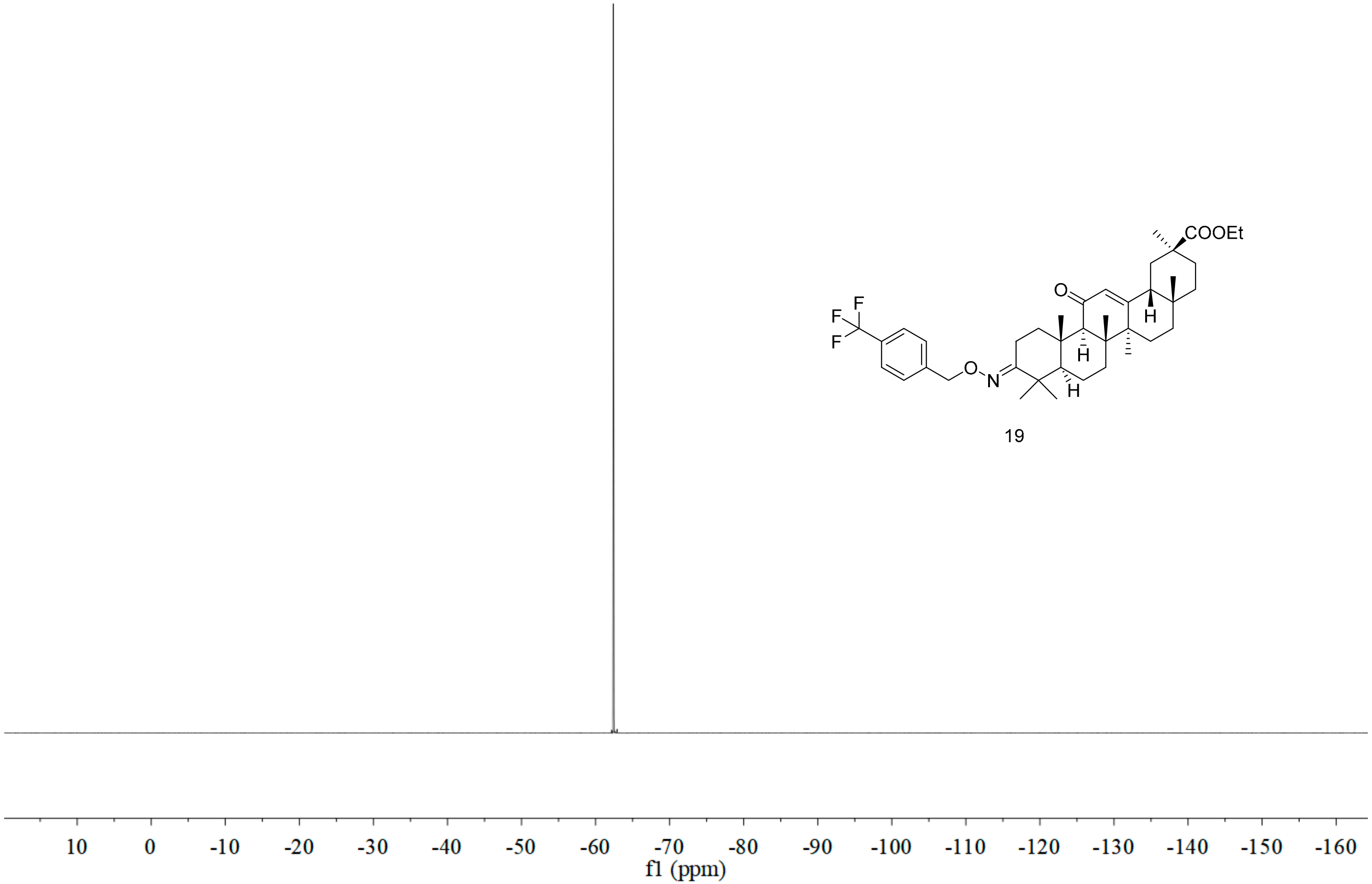

19

^{19}F NMR of Compound **19** (377 MHz, $CDCl_3$)

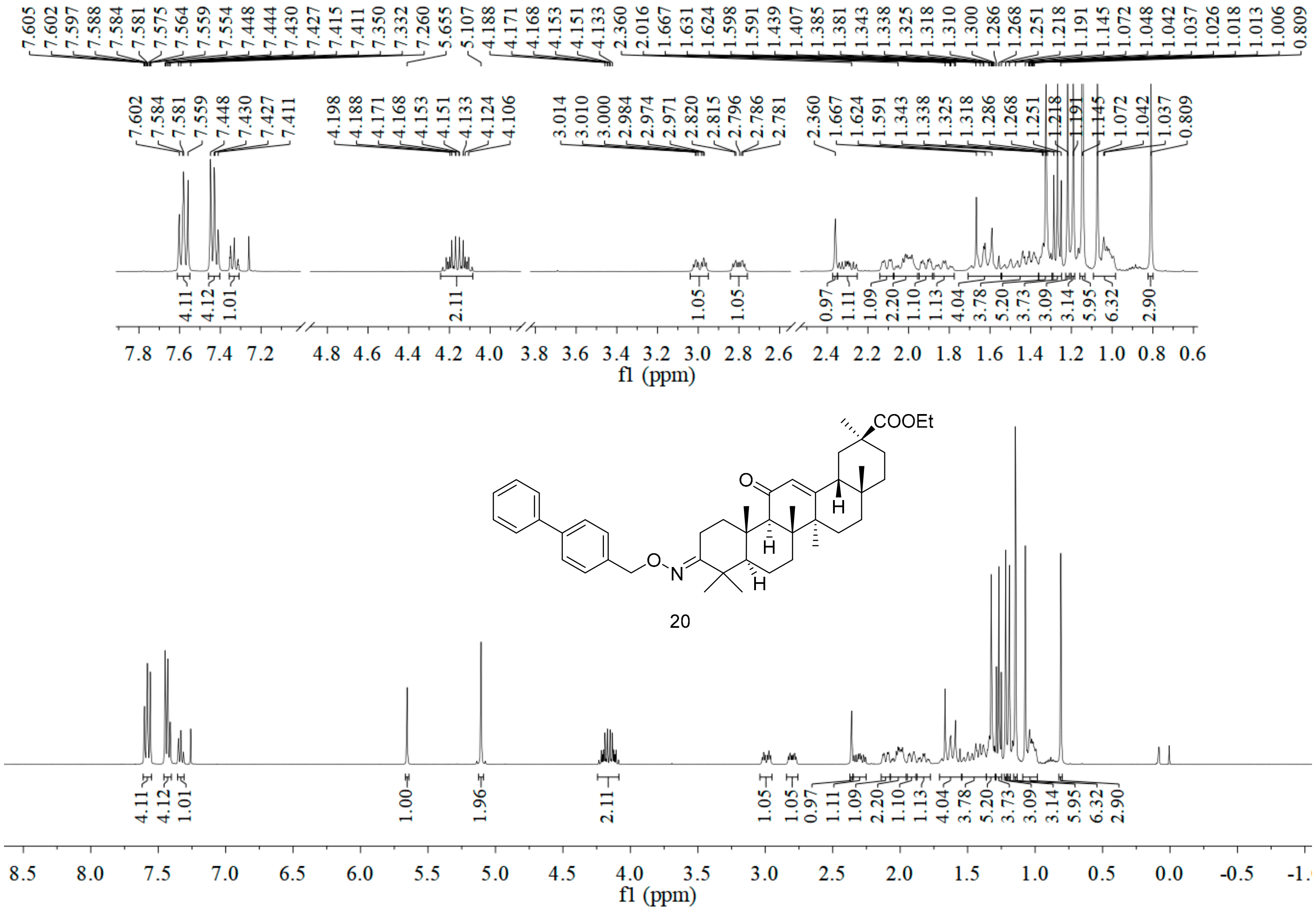

^{1}H NMR of Compound **20** (400 MHz, $CDCl_3$)

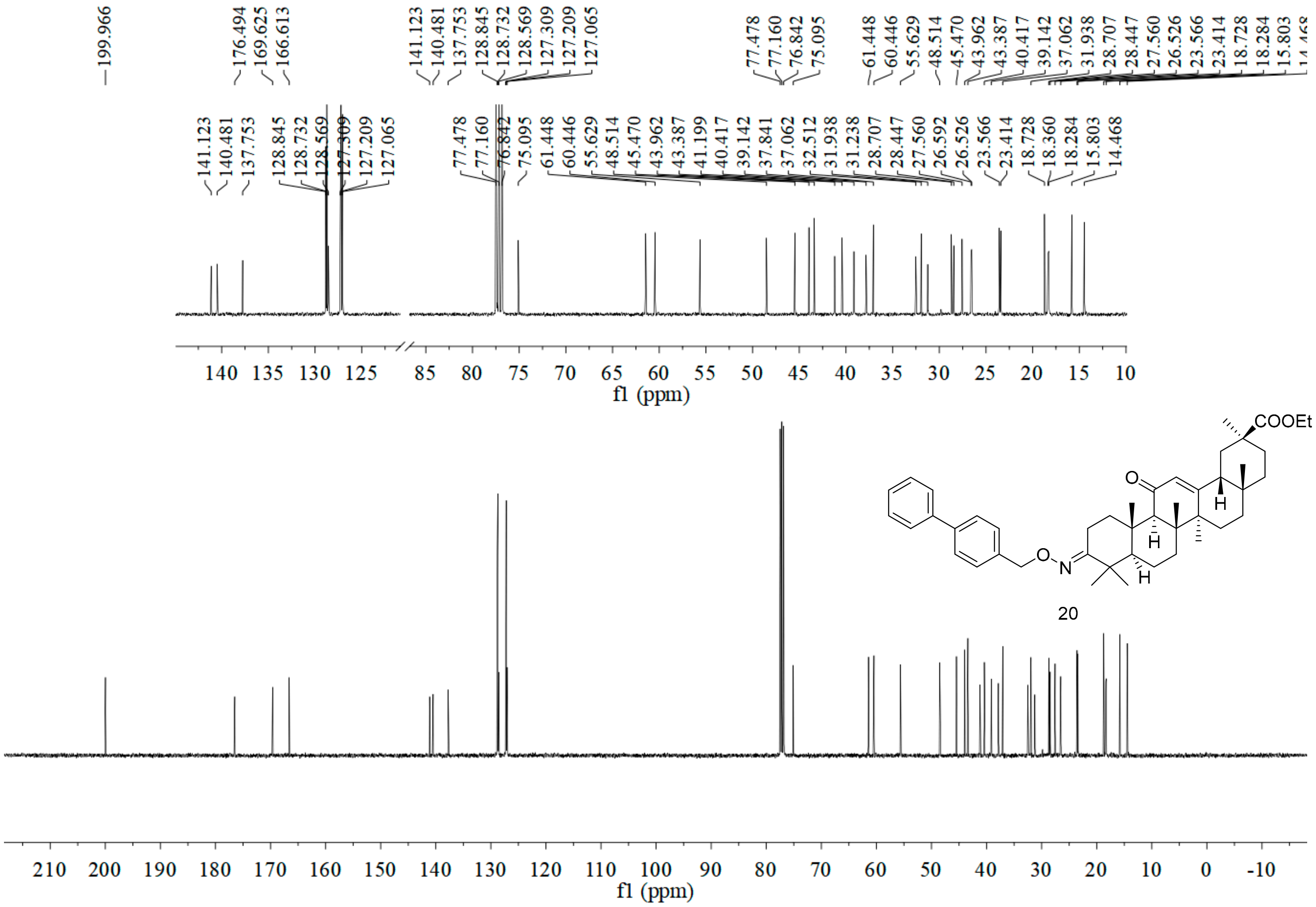

^{13}C NMR of Compound **20** (100 MHz, $CDCl_3$)

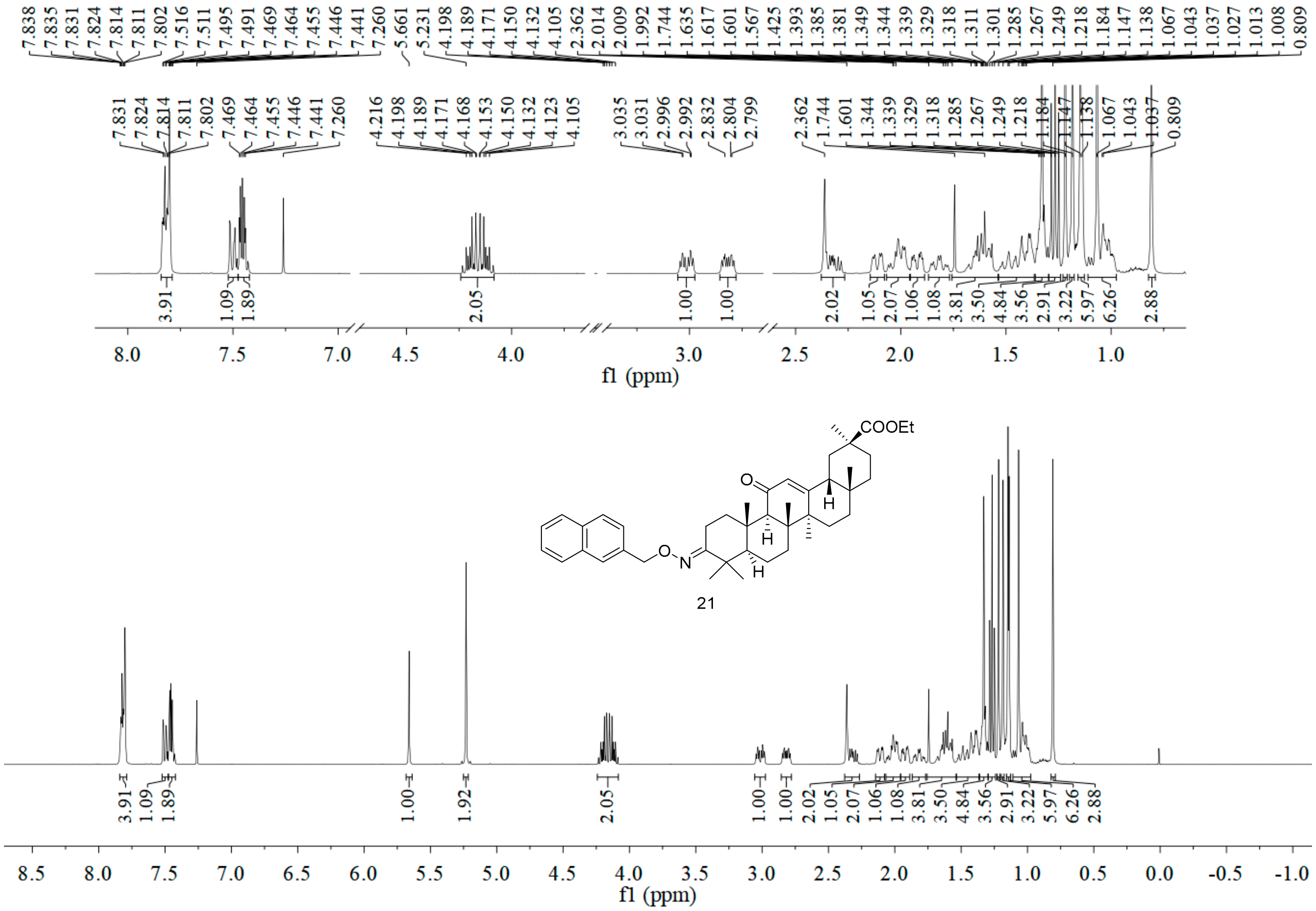

^{1}H NMR of Compound **21** (400 MHz, $CDCl_3$)

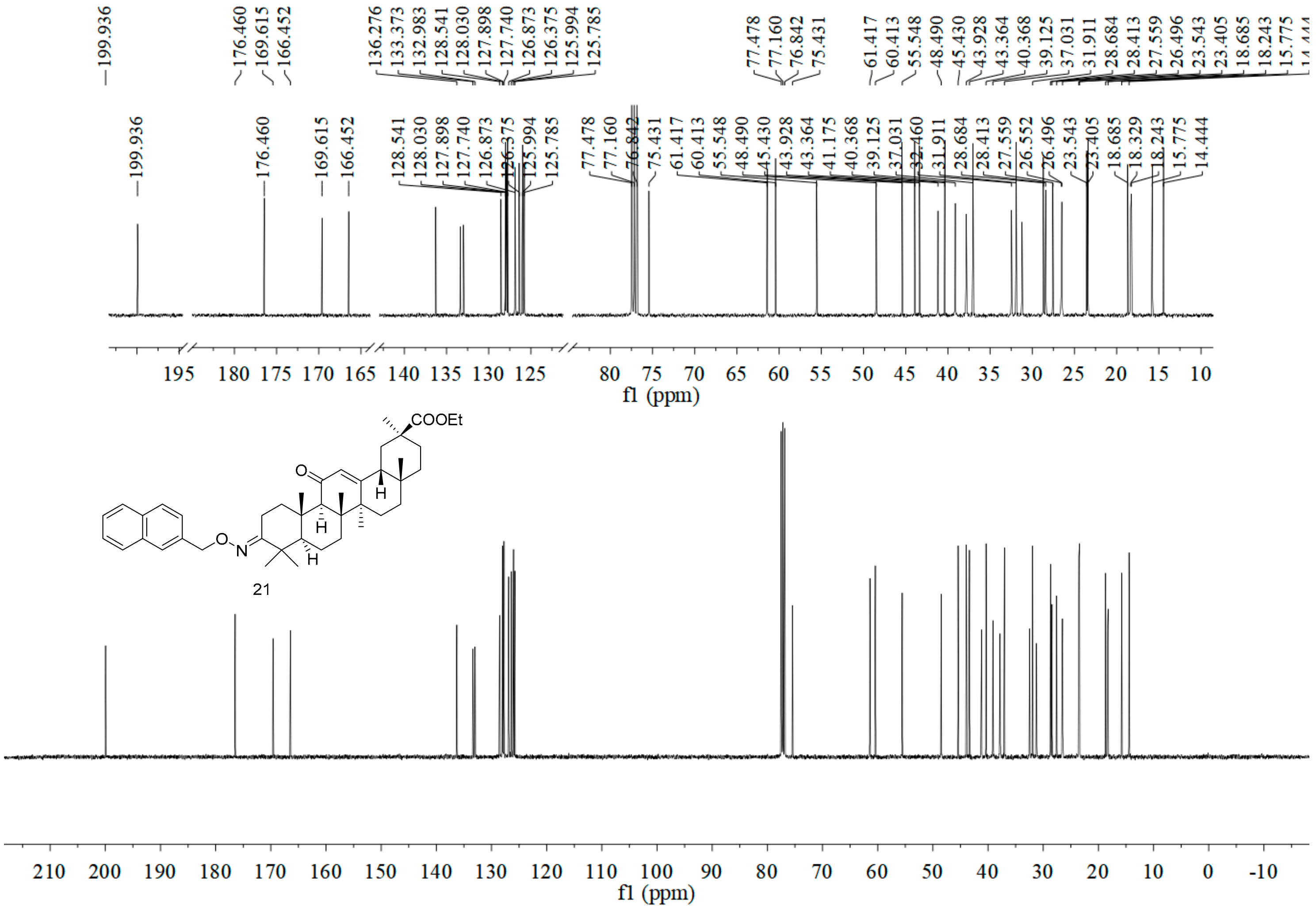

^{13}C NMR of Compound **21** (100 MHz, $CDCl_3$)

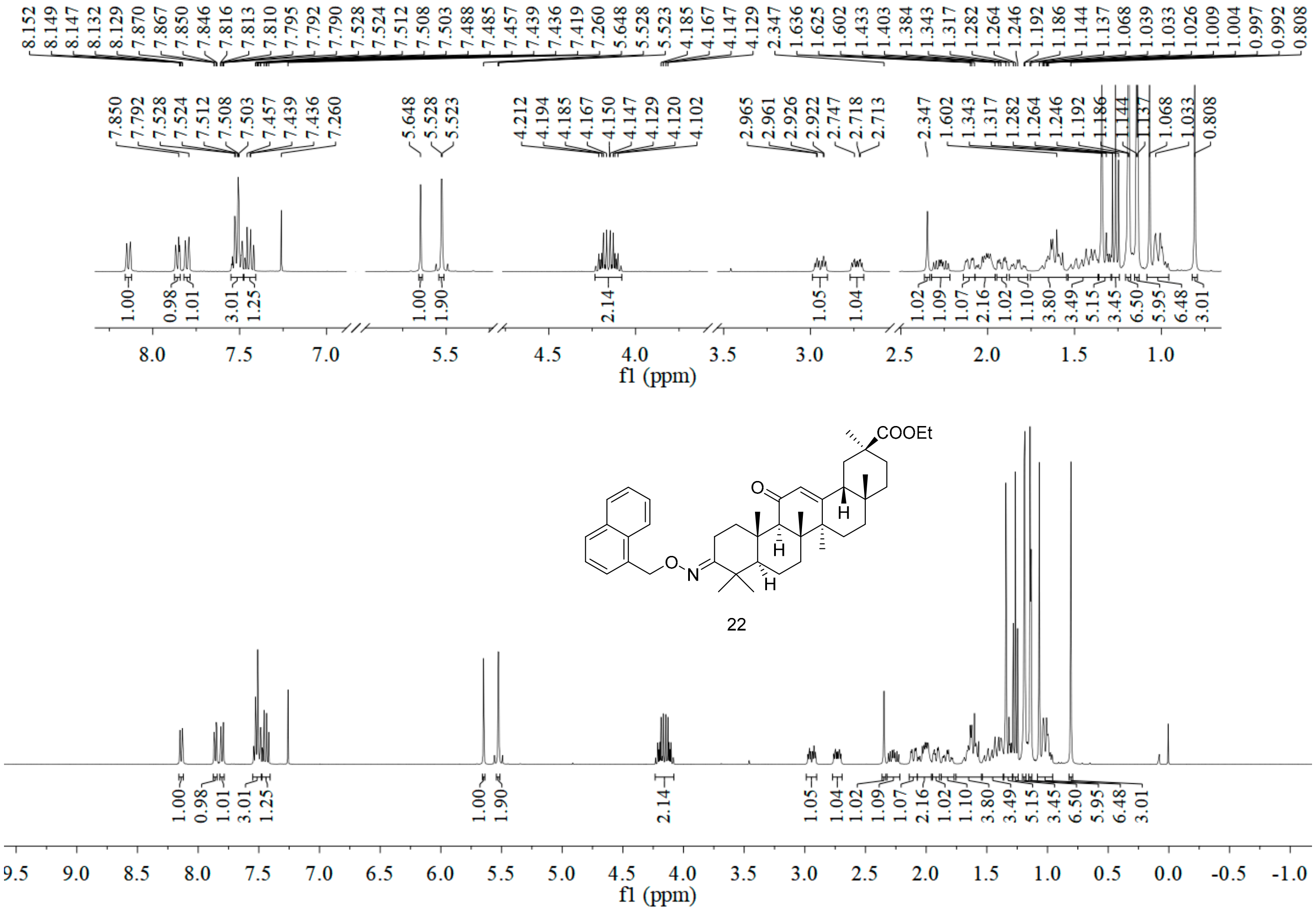

^{1}H NMR of Compound **22** (400 MHz, $CDCl_3$)

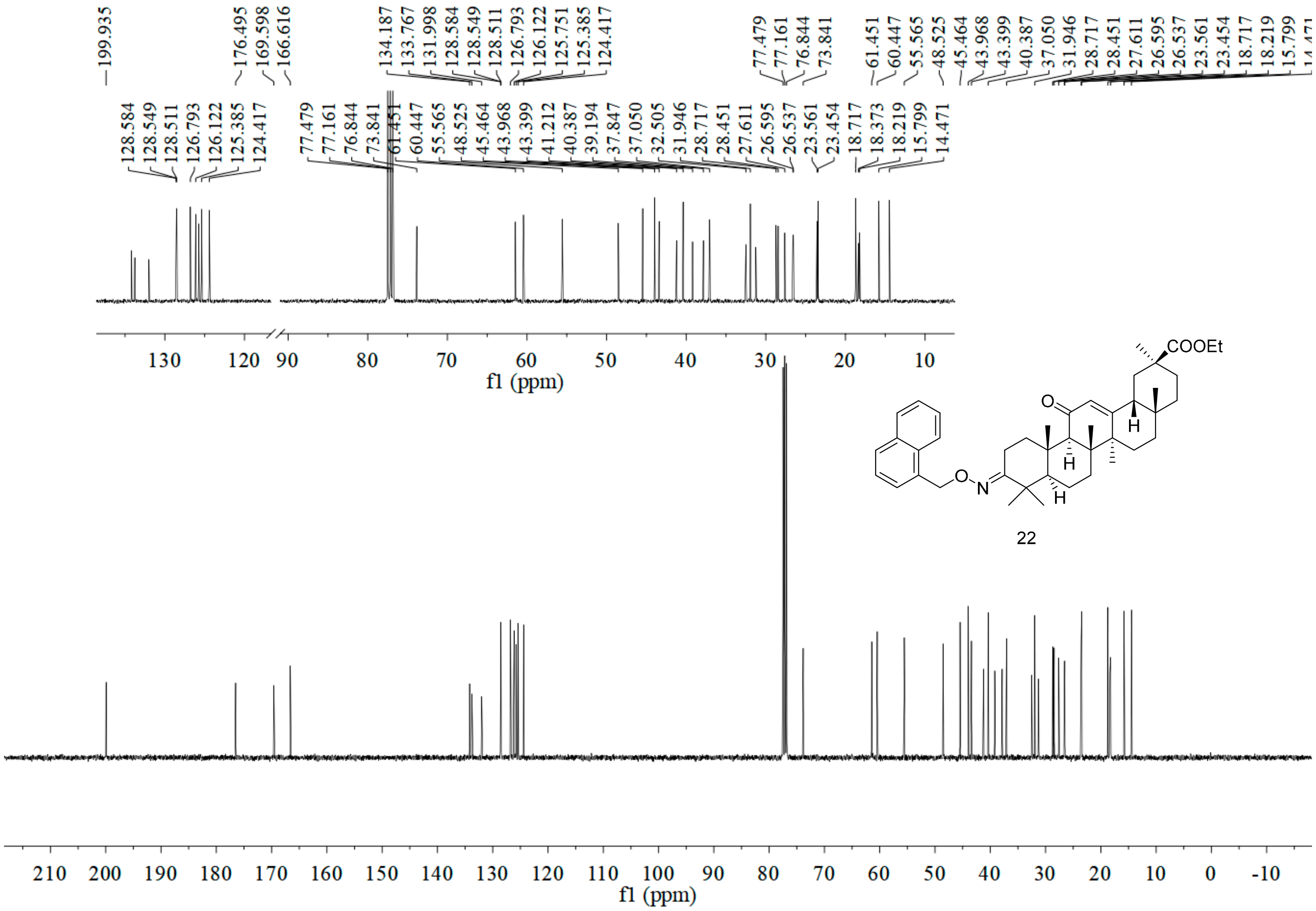

^{13}C NMR of Compound **22** (100 MHz, $CDCl_3$)

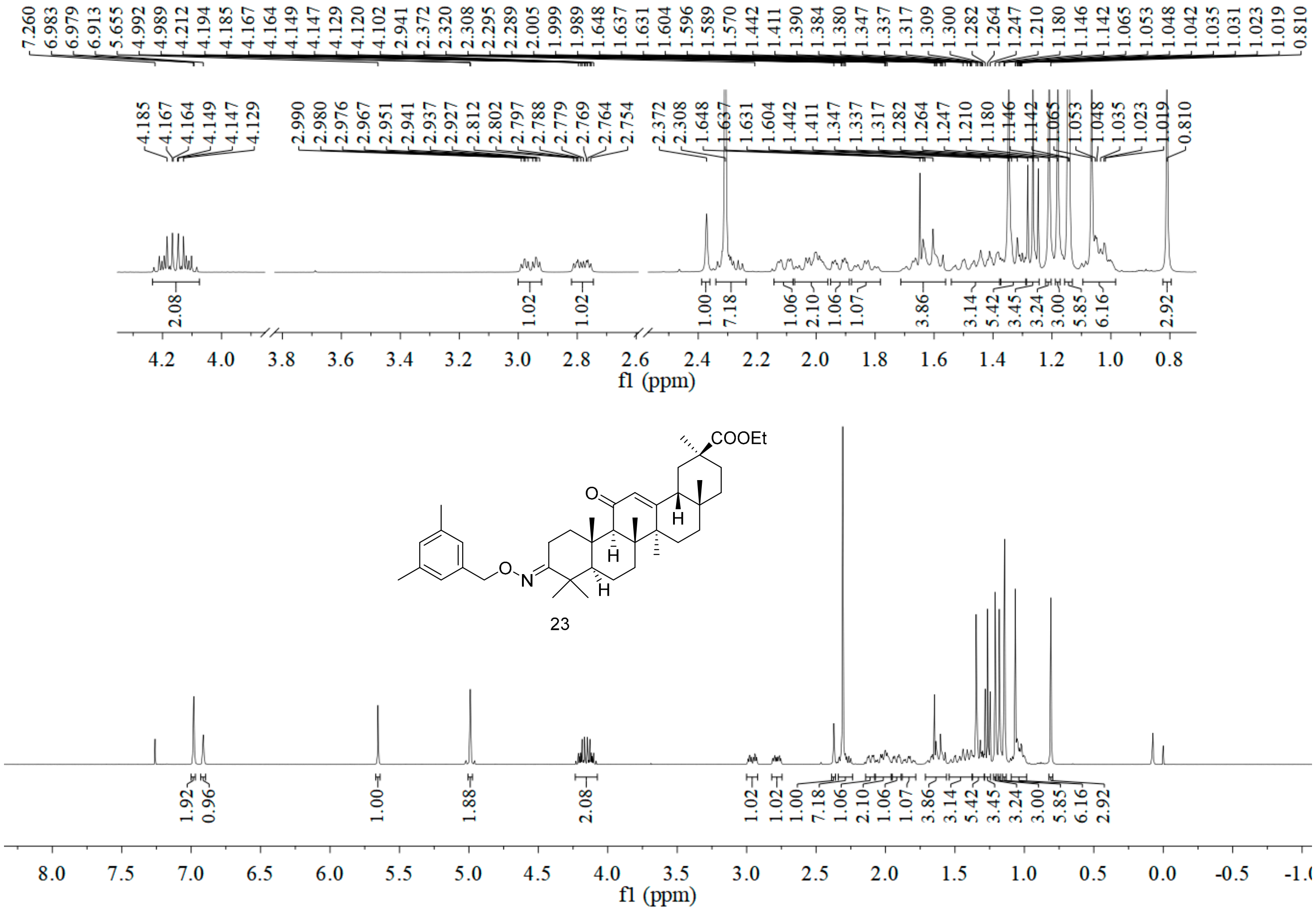

^{1}H NMR of Compound **23** (400 MHz, $CDCl_3$)

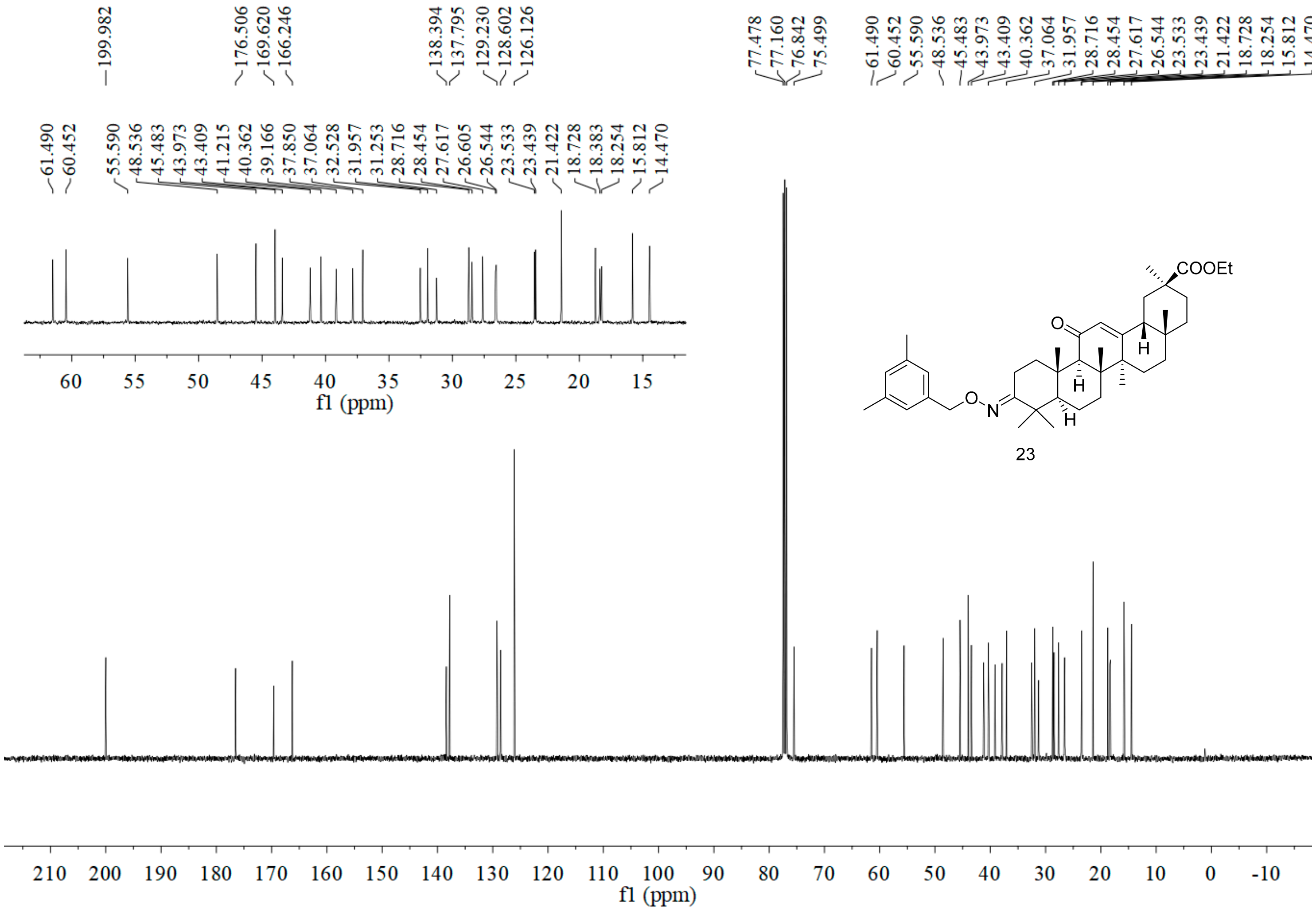

^{13}C NMR of Compound **23** (100 MHz, $CDCl_3$)

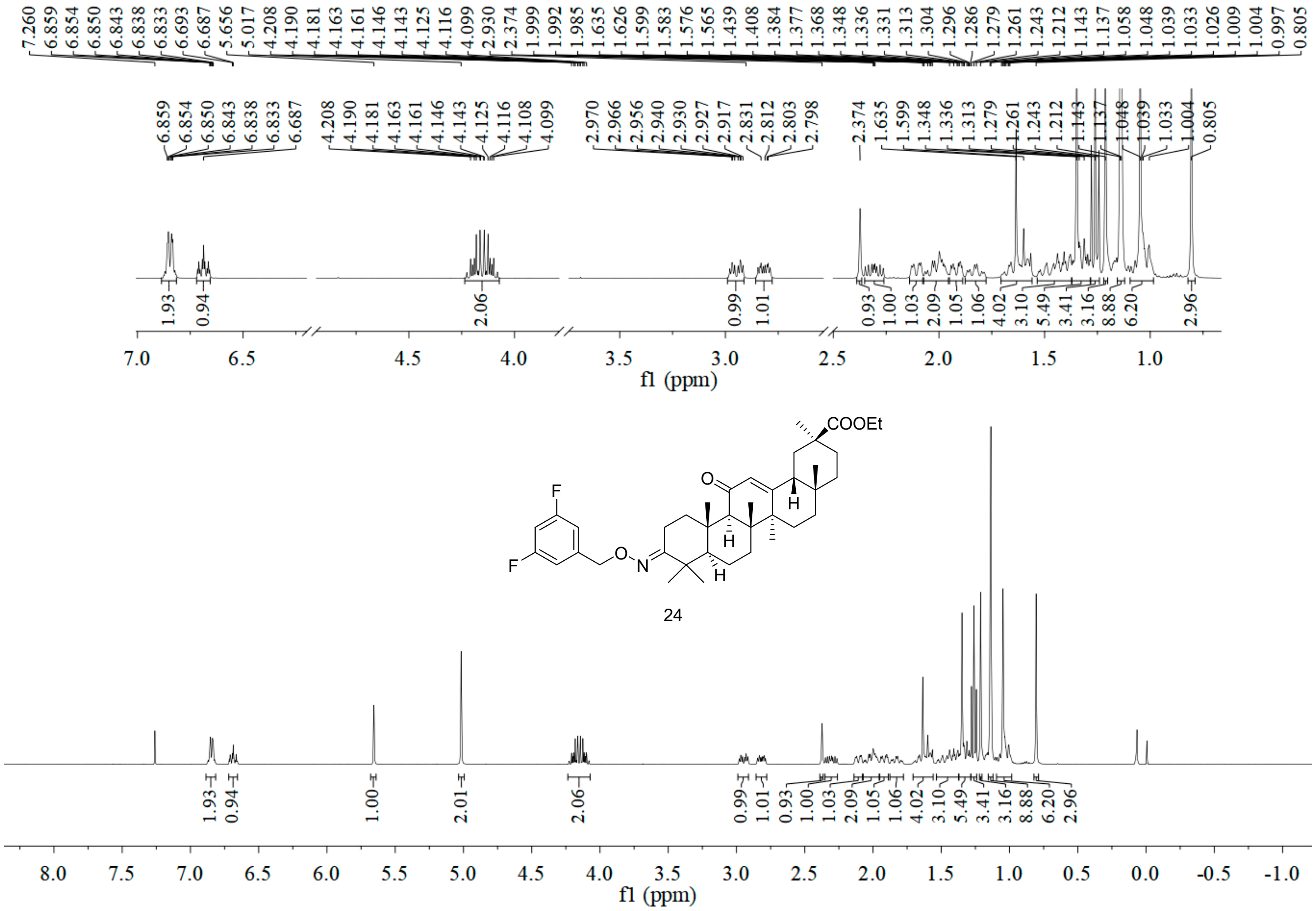

24

^{1}H NMR of Compound **24** (400 MHz, $CDCl_3$)

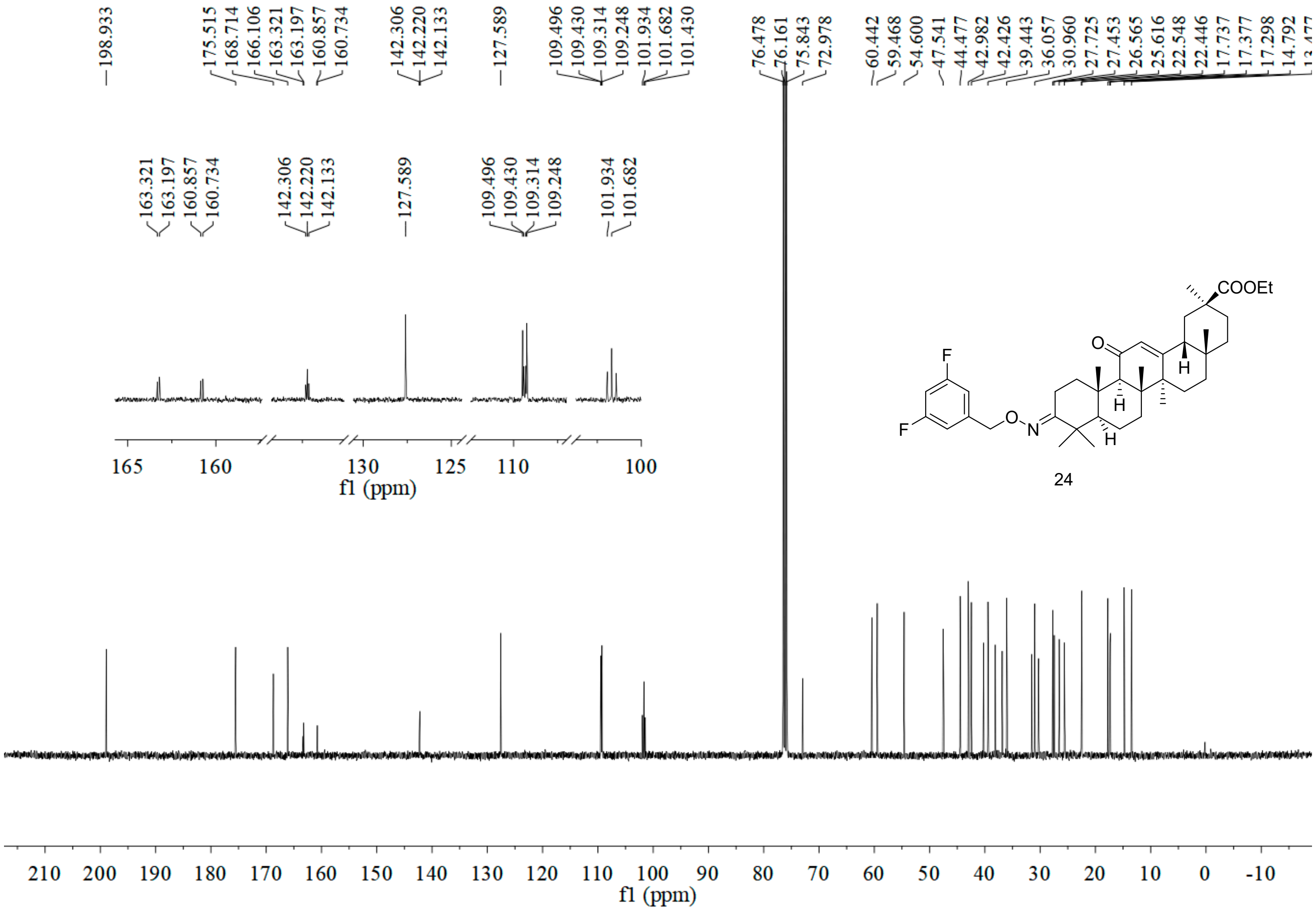

^{13}C NMR of Compound **24** (100 MHz, $CDCl_3$)

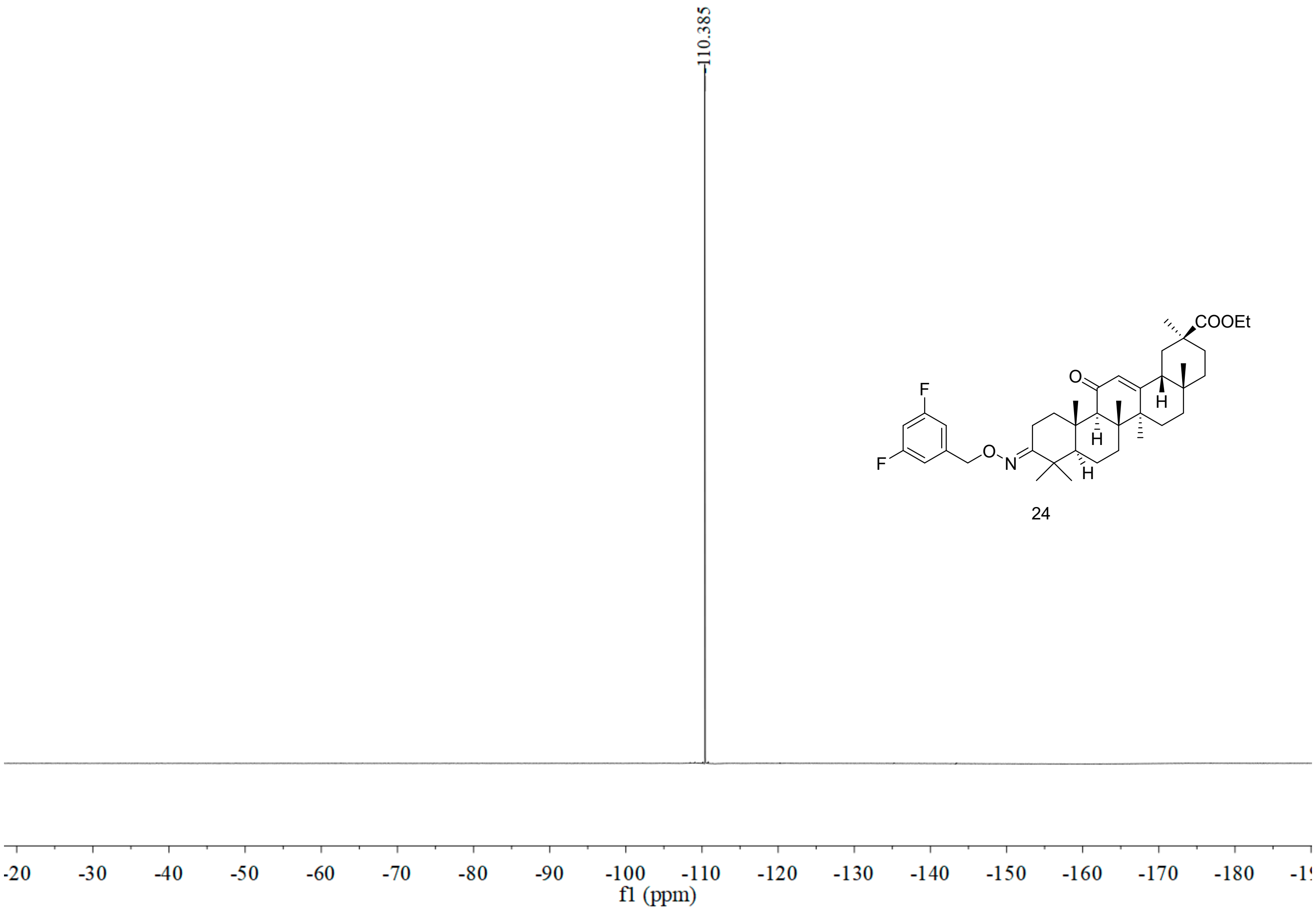

^{19}F NMR of Compound **24** (377 MHz, $CDCl_3$)

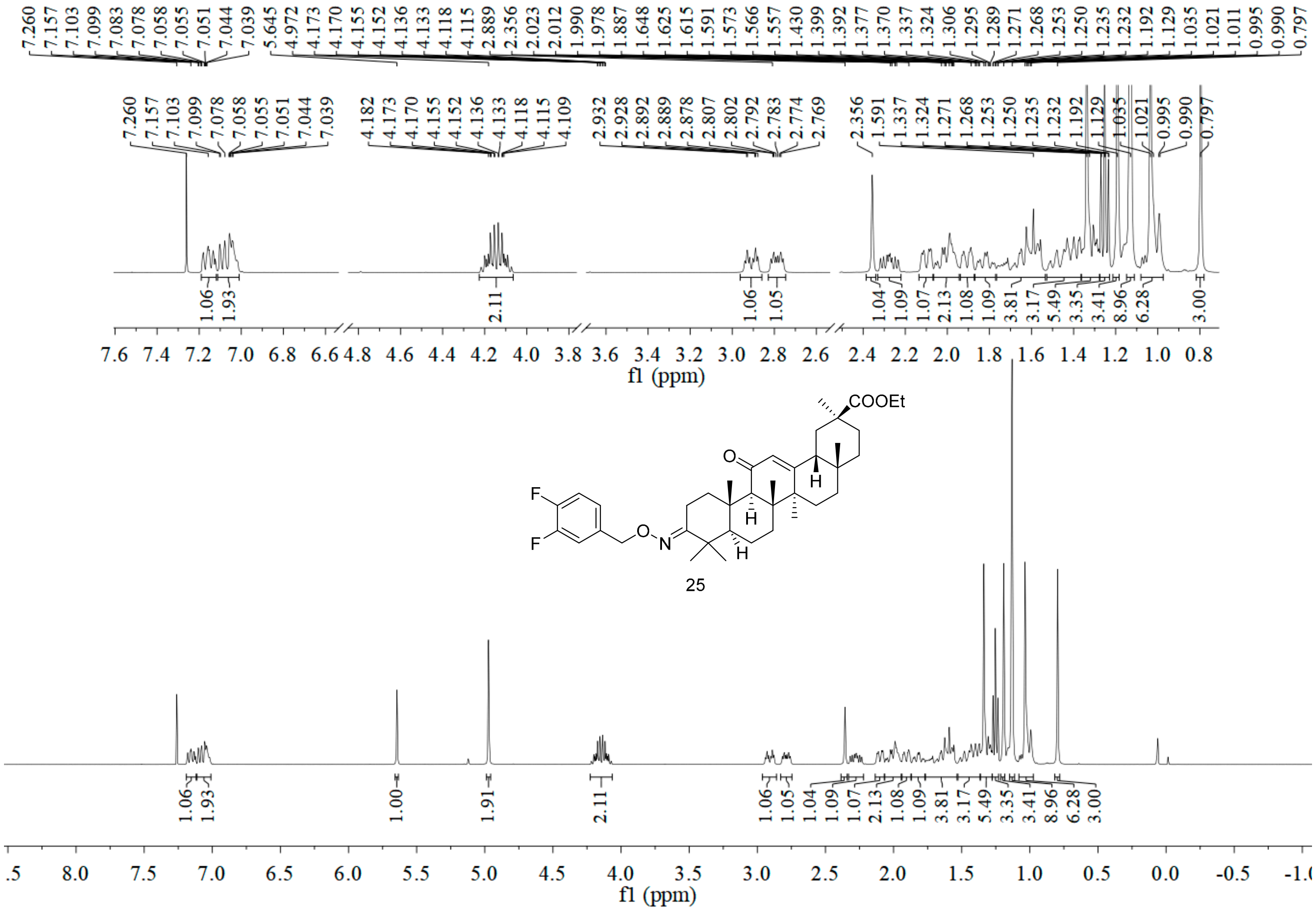

^{1}H NMR of Compound **25** (400 MHz, $CDCl_3$)

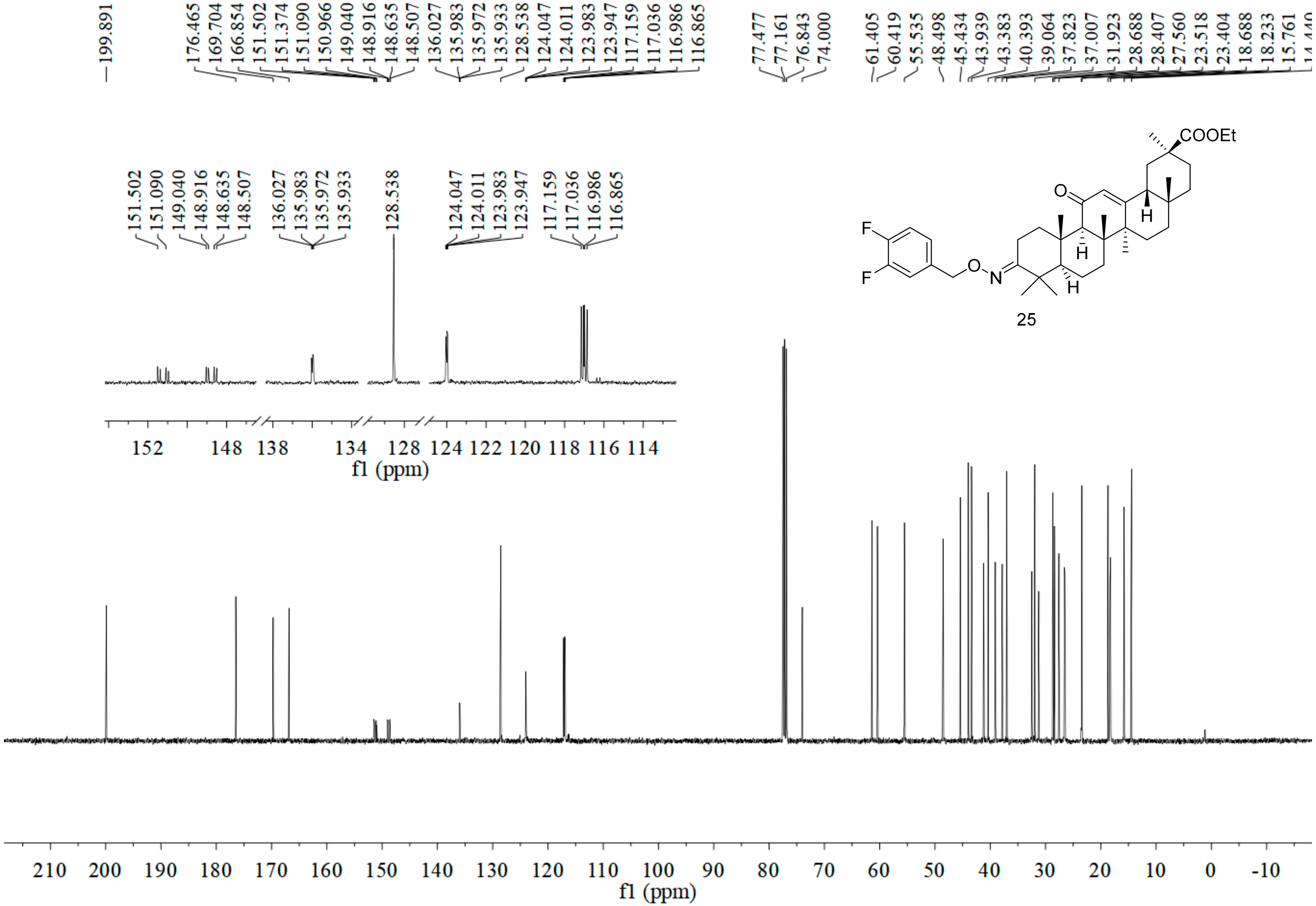

^{13}C NMR of Compound **25** (100 MHz, $CDCl_3$)

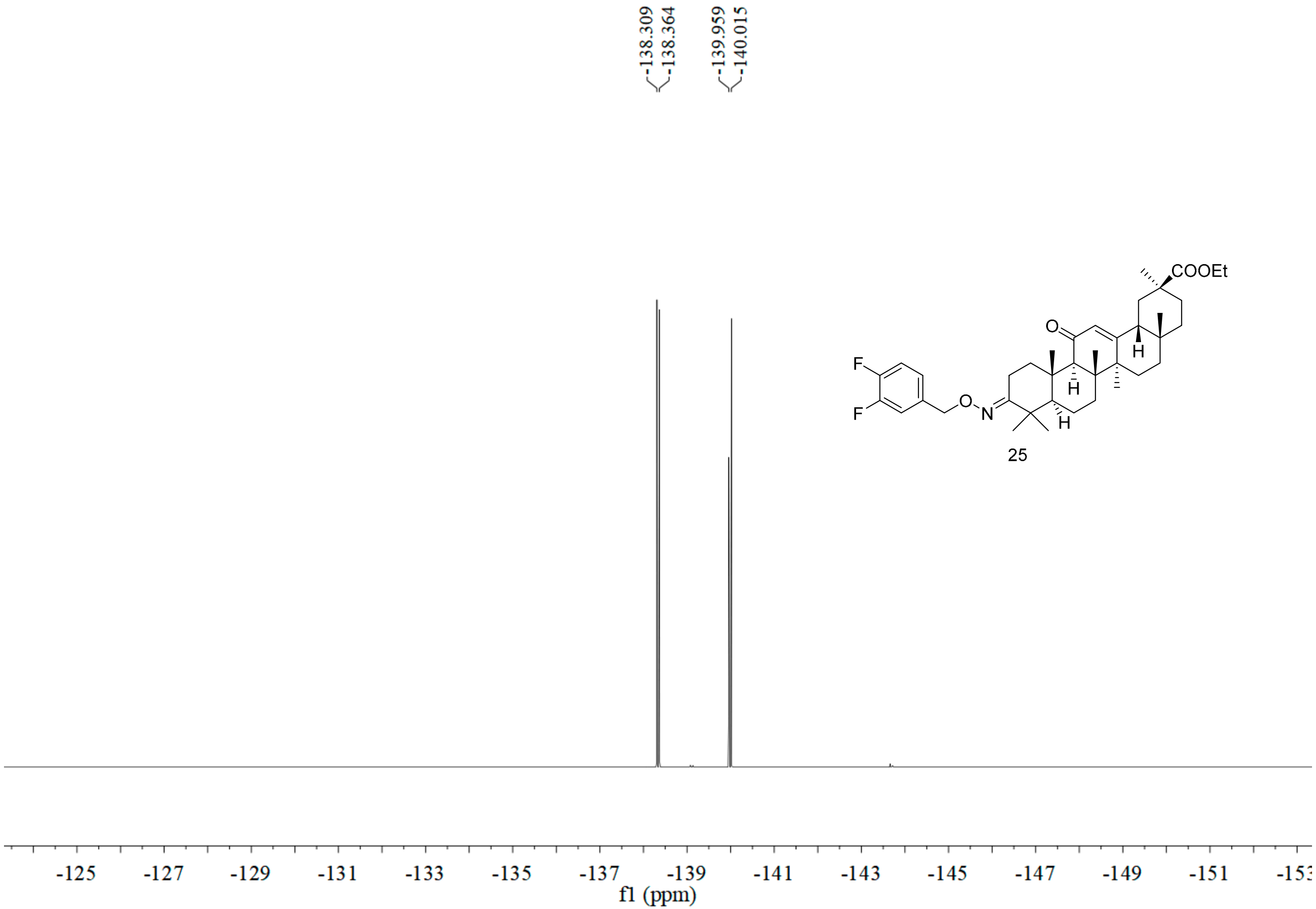

^{19}F NMR of Compound **25** (377 MHz, $CDCl_3$)

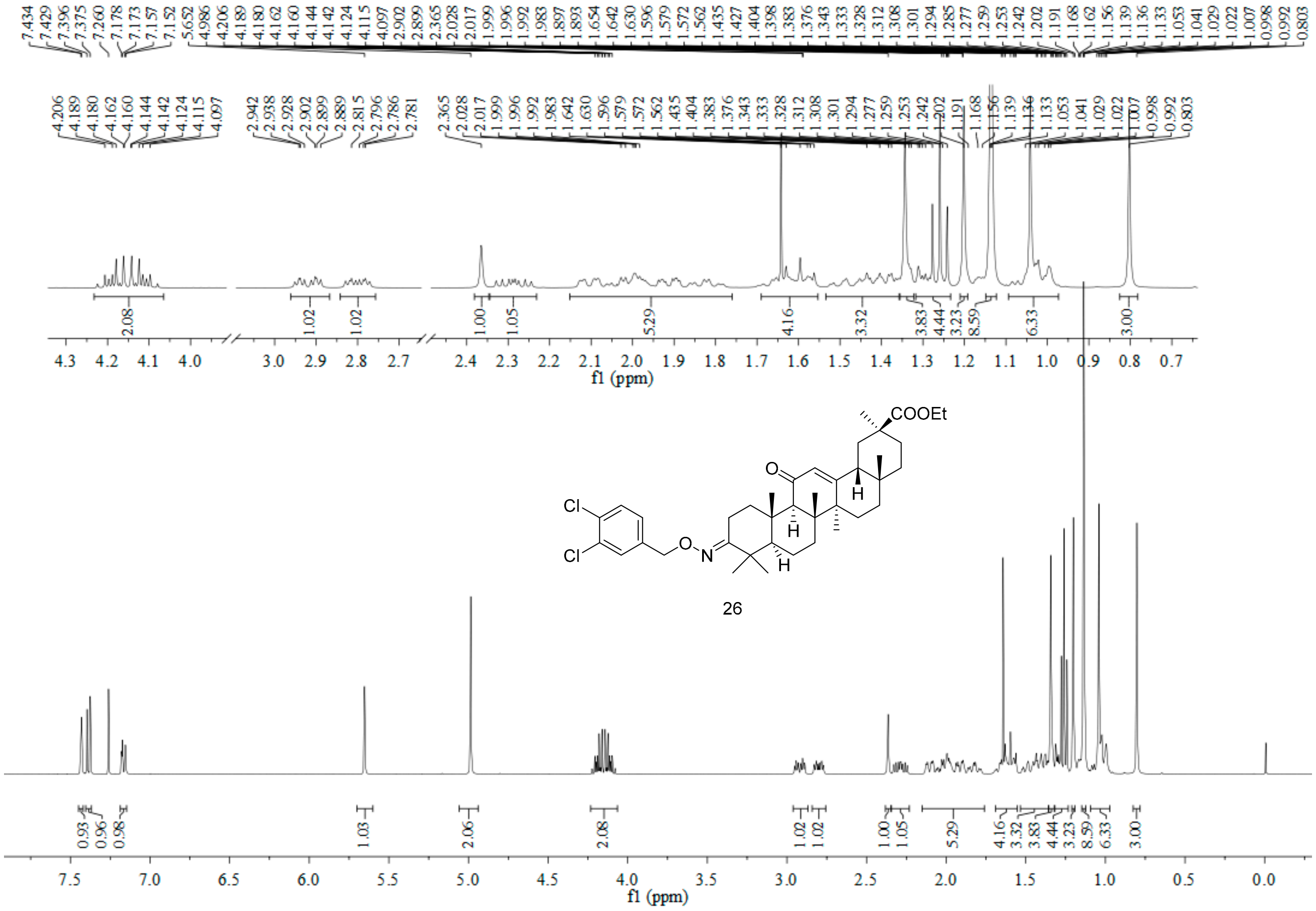

^{1}H NMR of Compound **26** (400 MHz, $CDCl_3$)

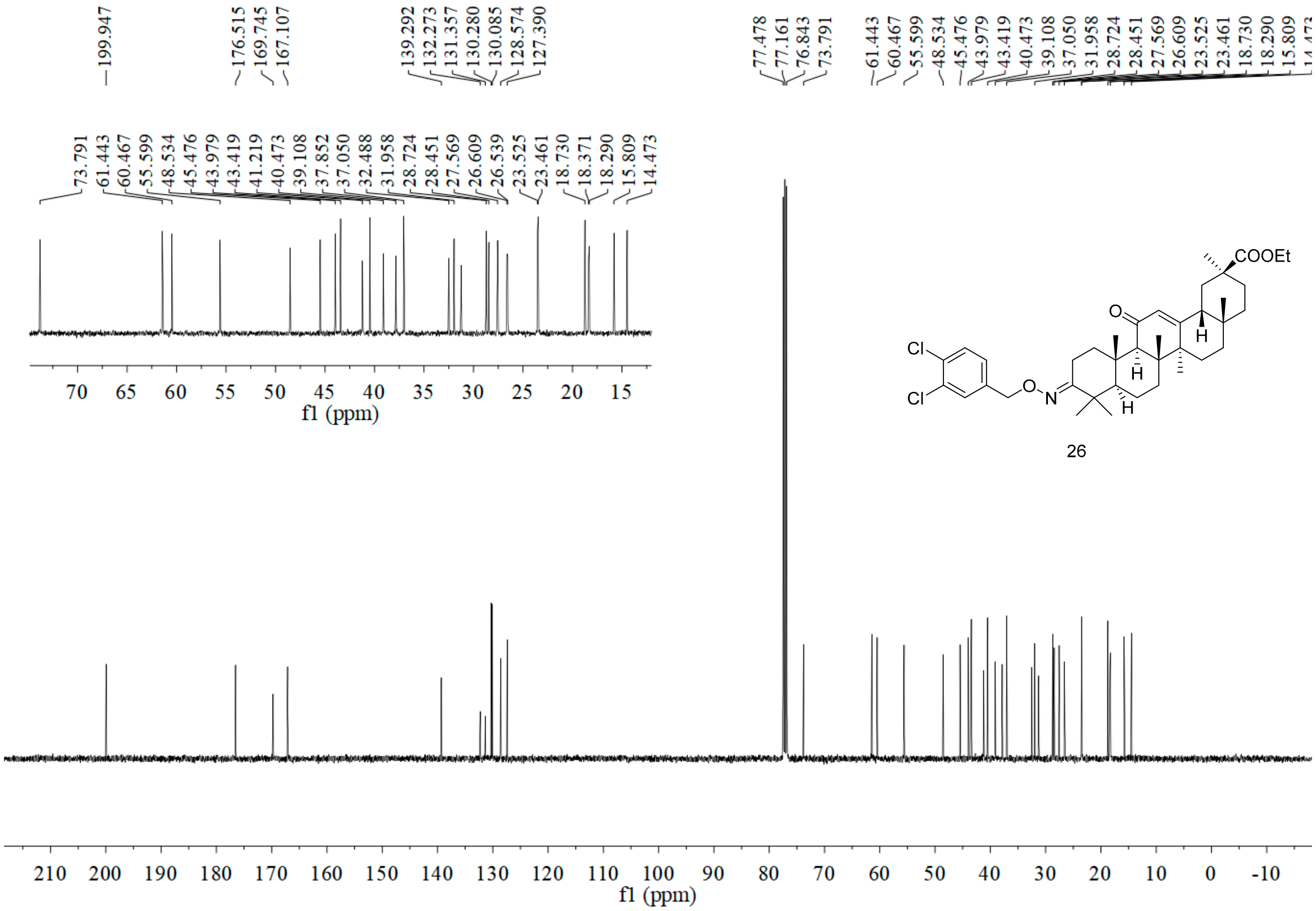

^{13}C NMR of Compound **26** (100 MHz, $CDCl_3$)

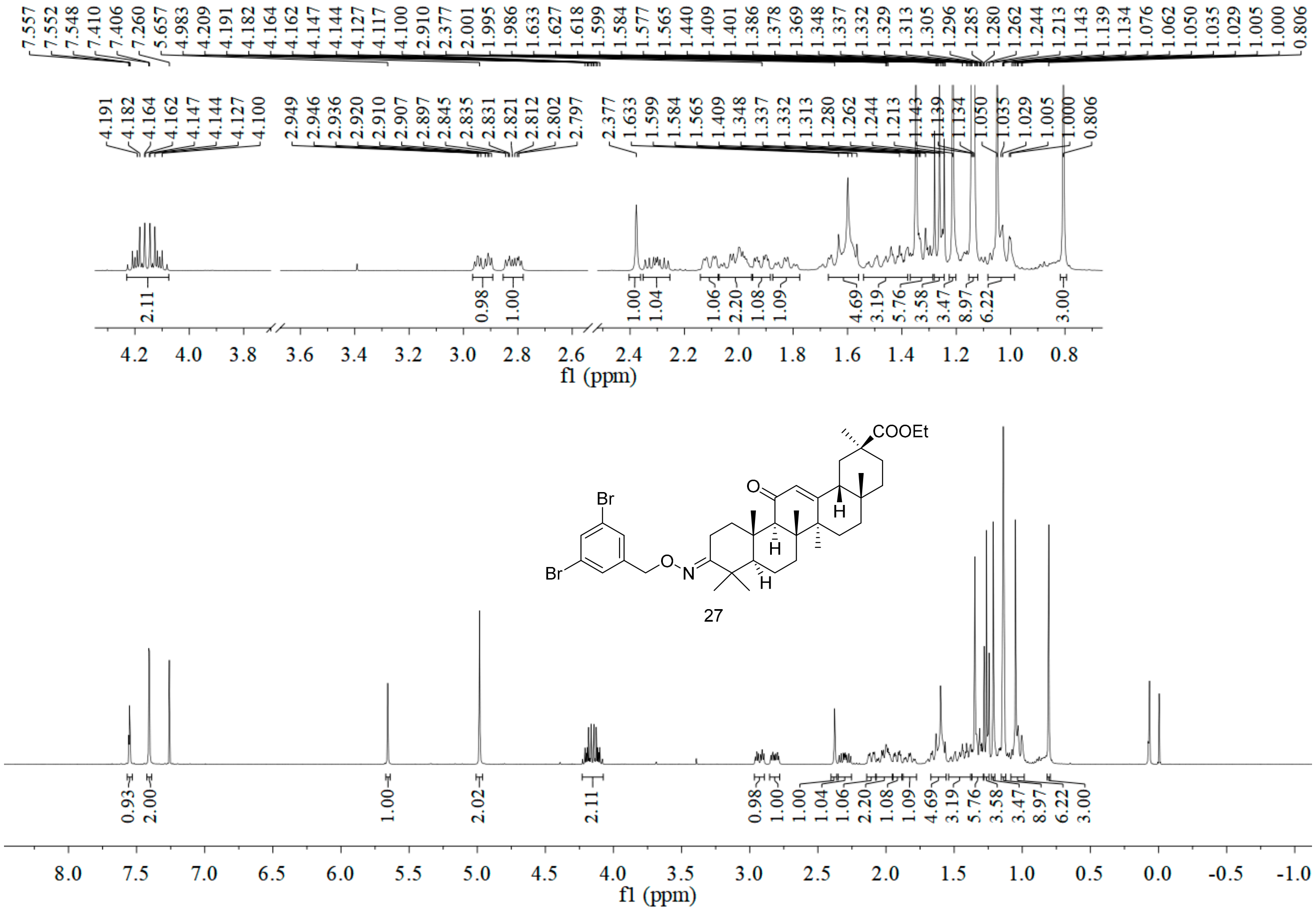

^{1}H NMR of Compound **27** (400 MHz, $CDCl_3$)

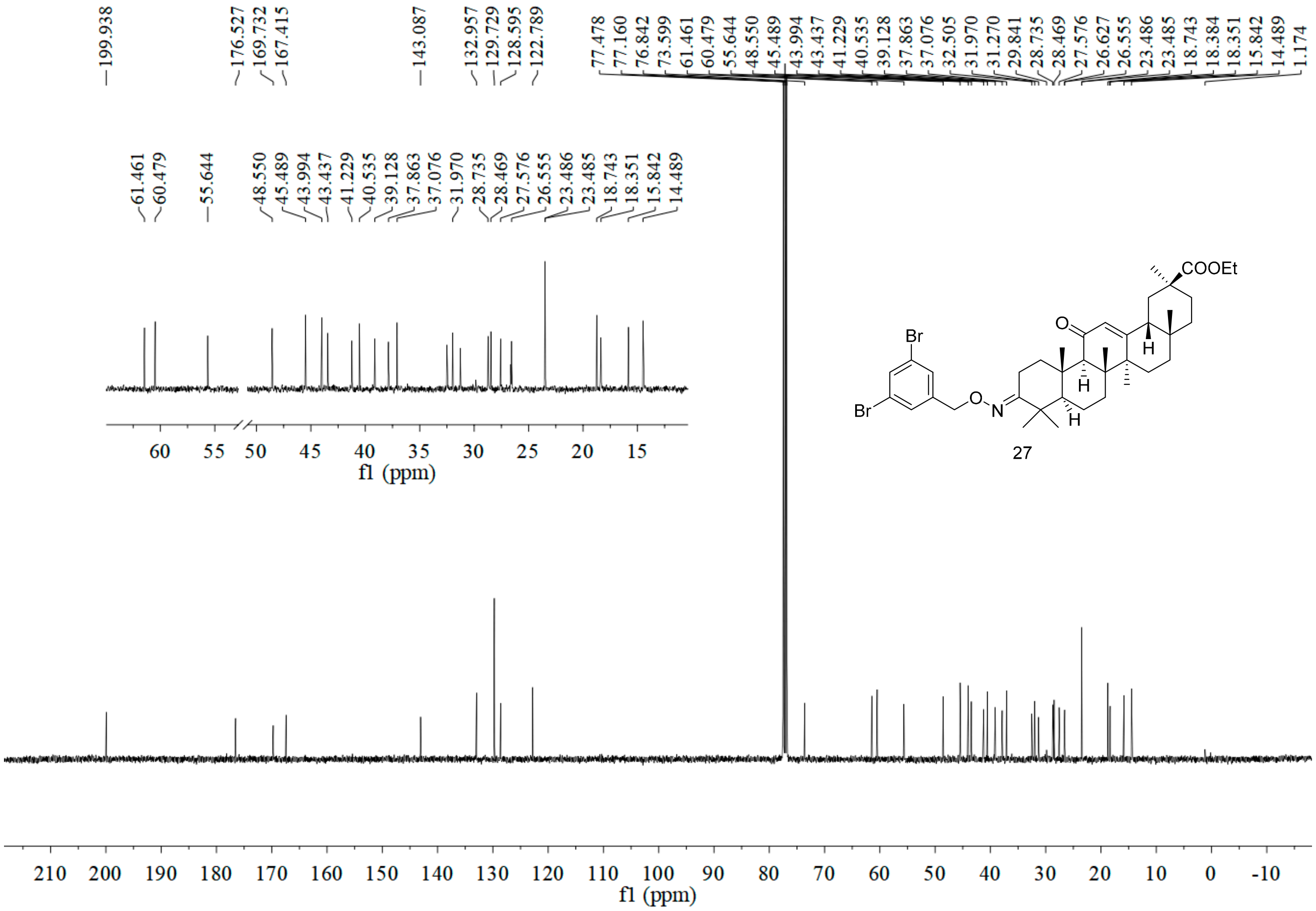

^{13}C NMR of Compound **27** (100 MHz, $CDCl_3$)

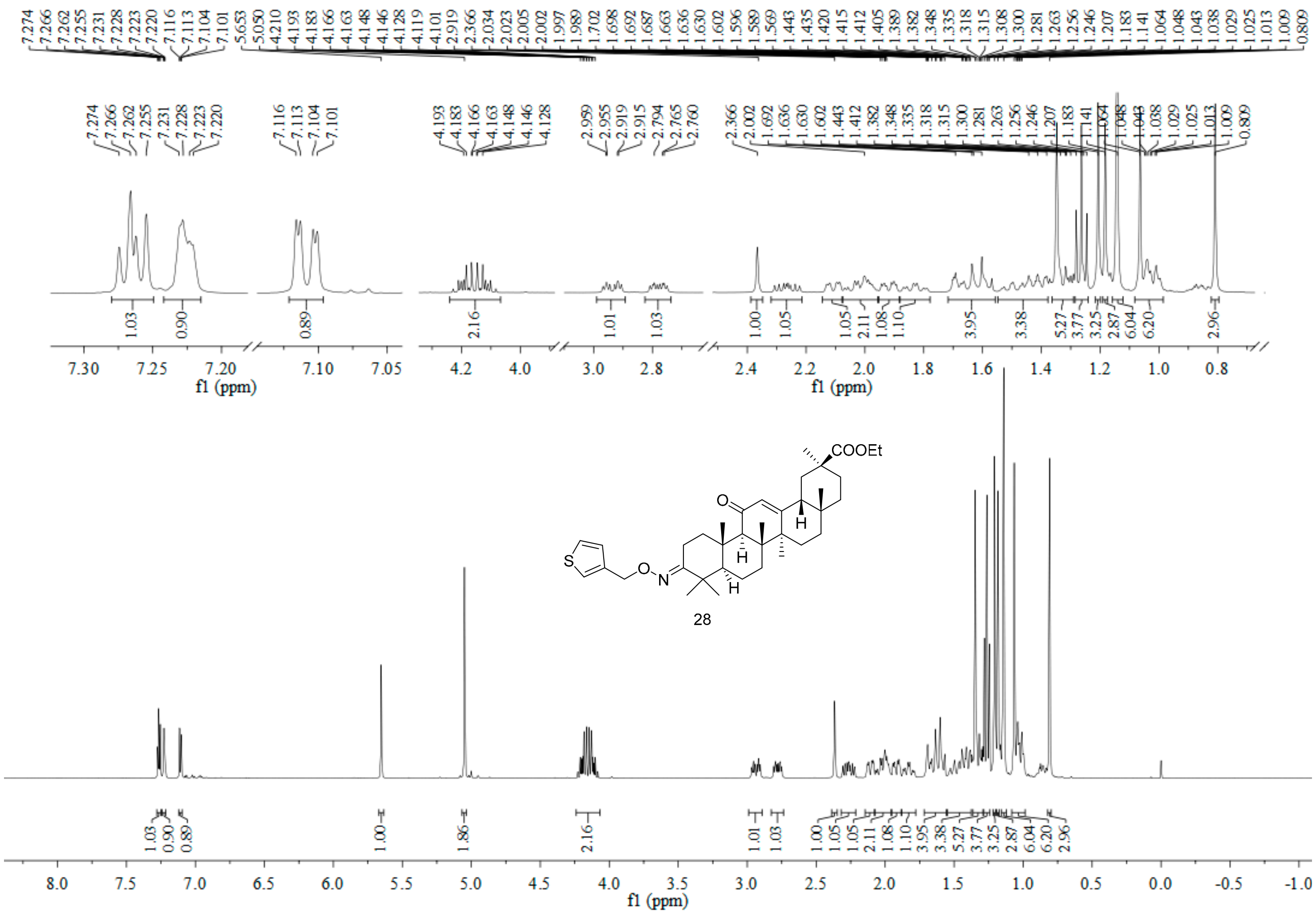

^{1}H NMR of Compound **28** (400 MHz, $CDCl_3$)

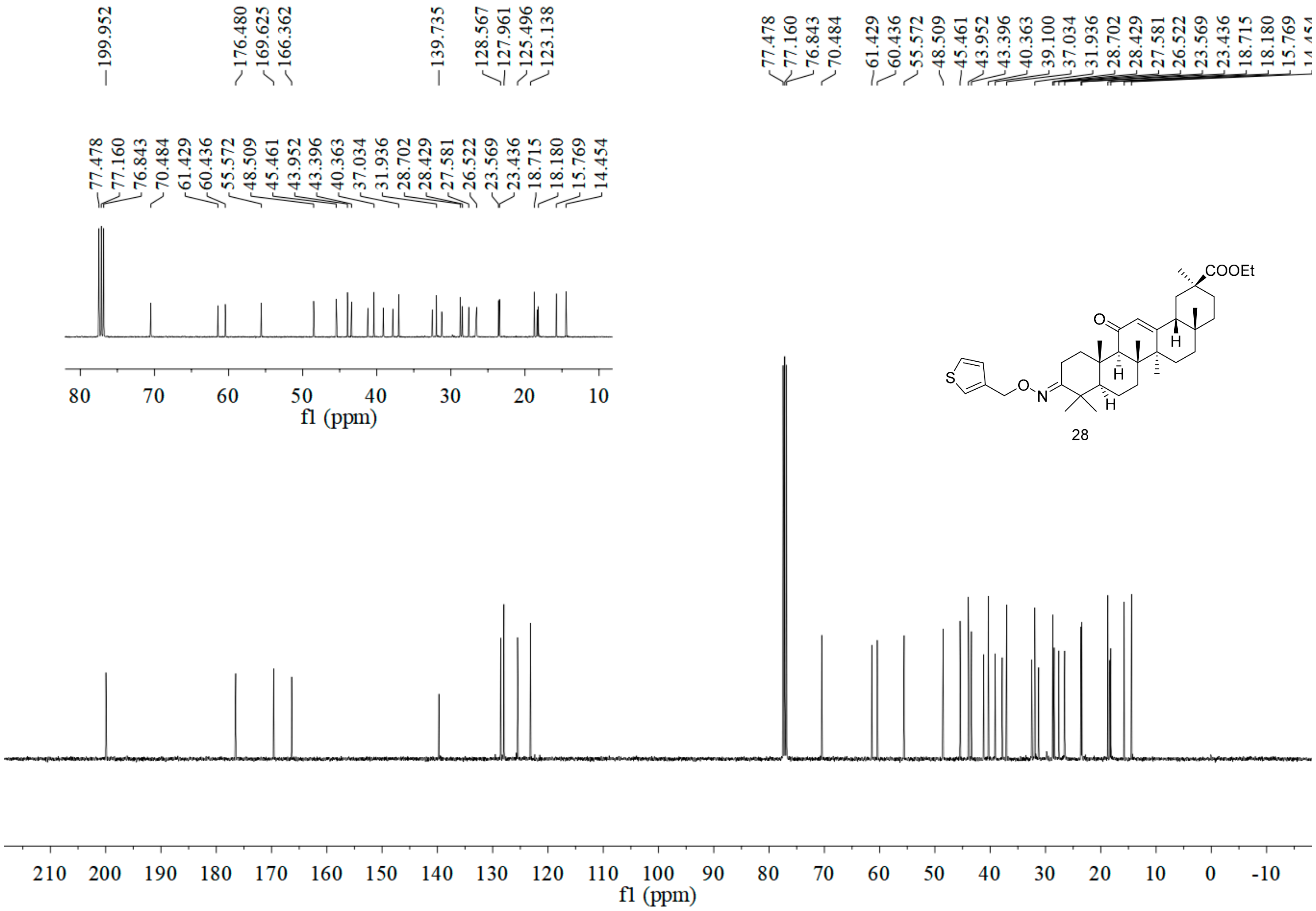

^{13}C NMR of Compound **28** (100 MHz, $CDCl_3$)

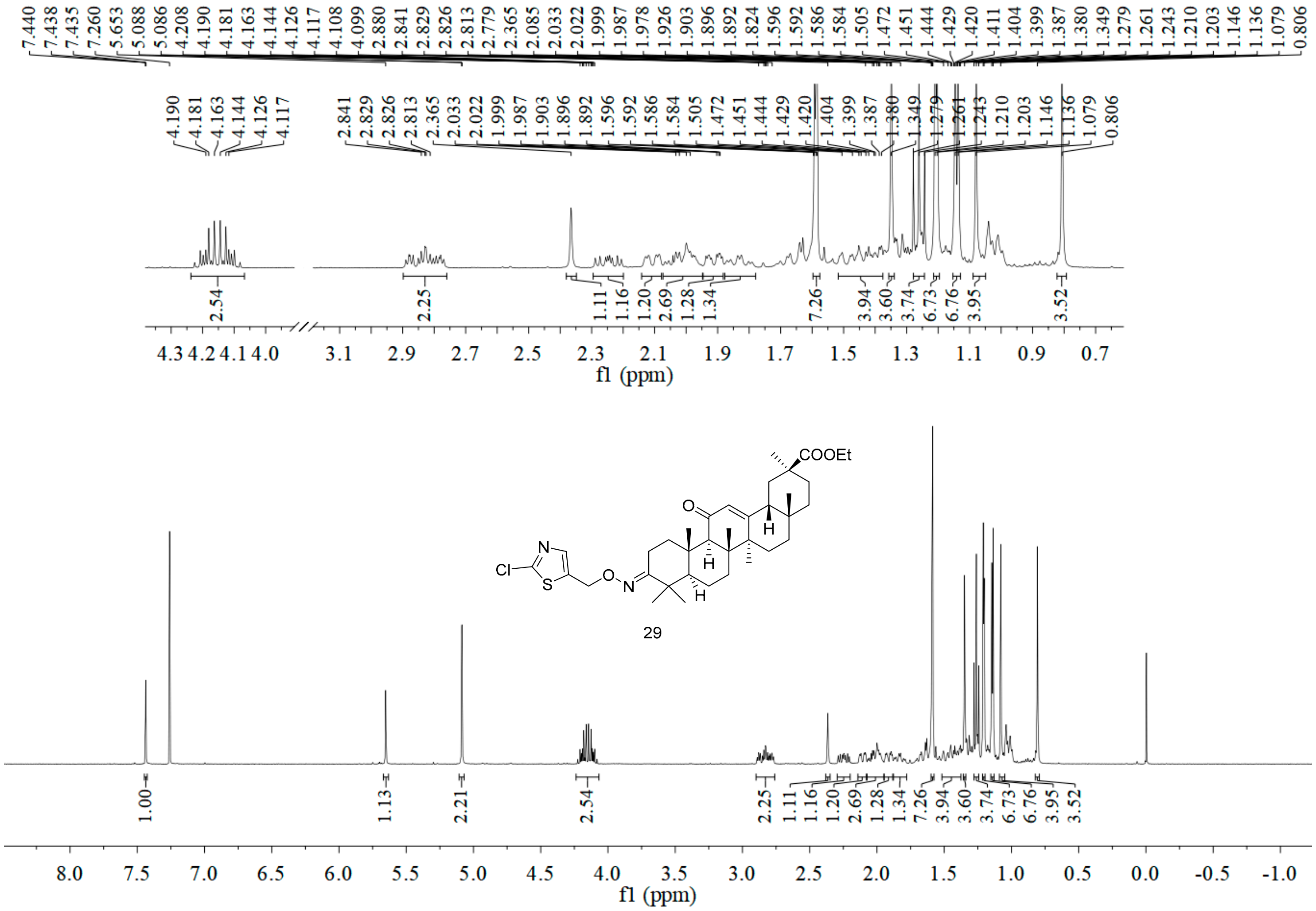

^{1}H NMR of Compound **29** (400 MHz, $CDCl_3$)

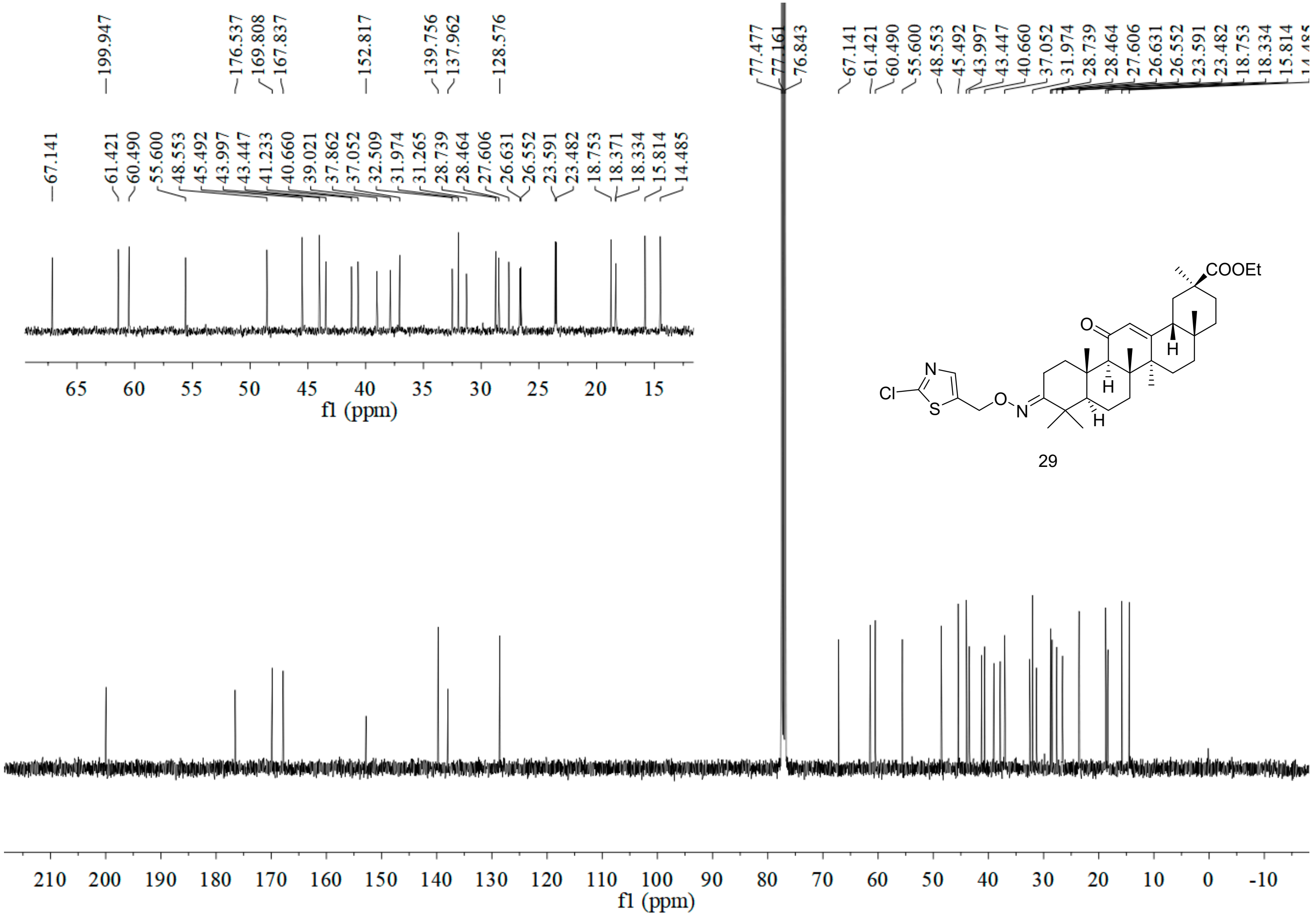

^{13}C NMR of Compound **29** (100 MHz, $CDCl_3$)

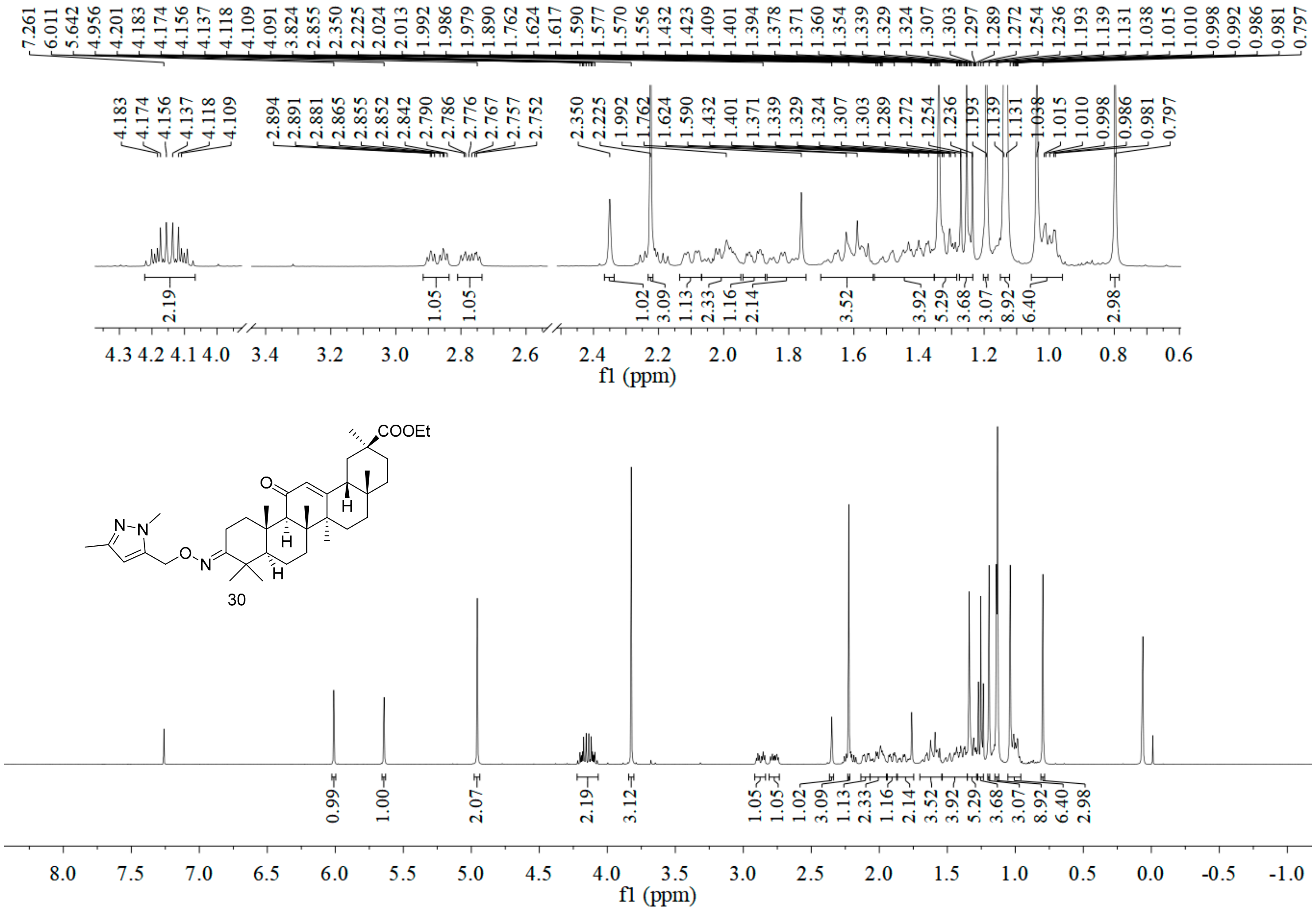

^1H NMR of Compound **30** (400 MHz, $CDCl_3$)

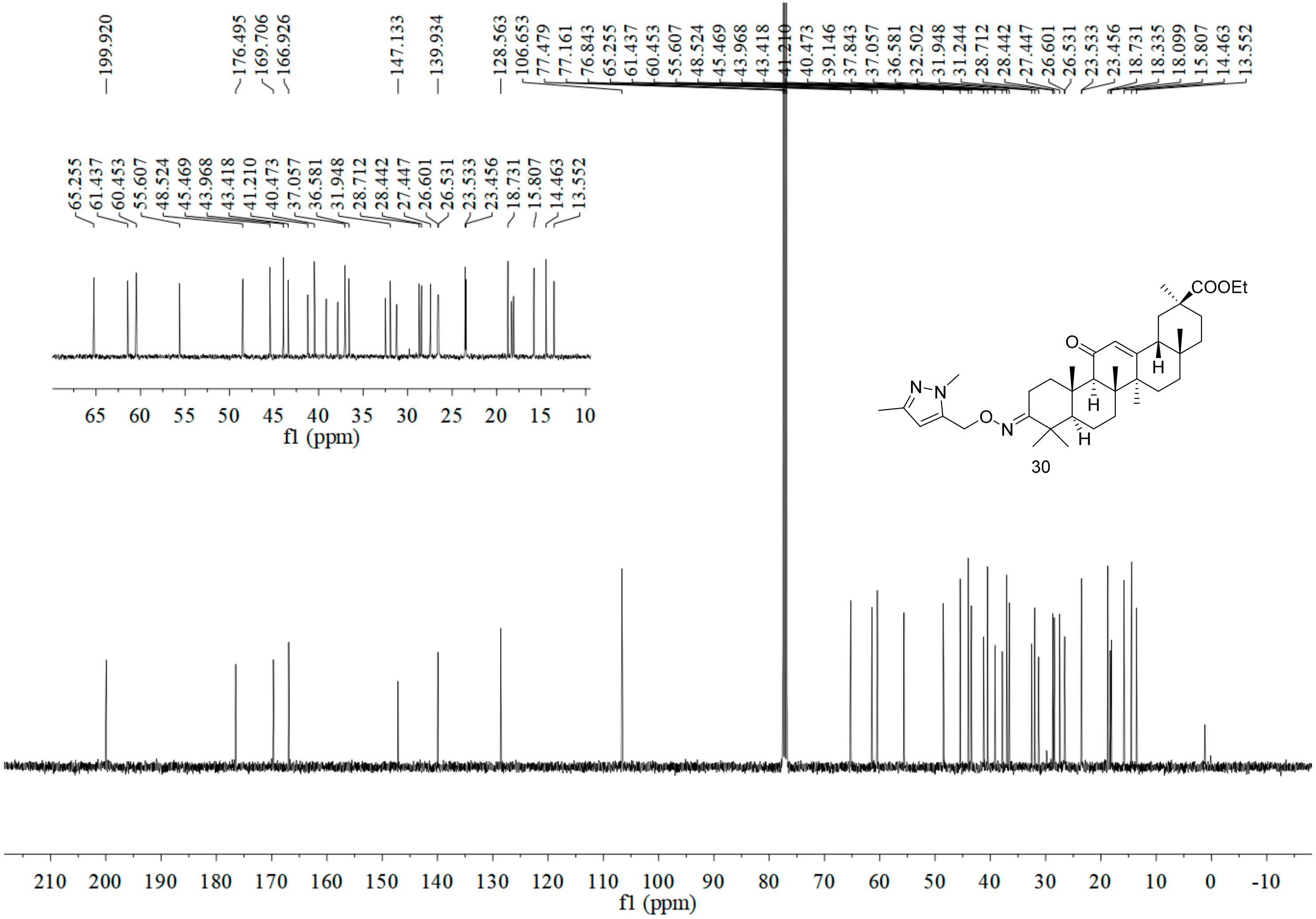

^{13}C NMR of Compound **30** (100 MHz, $CDCl_3$)

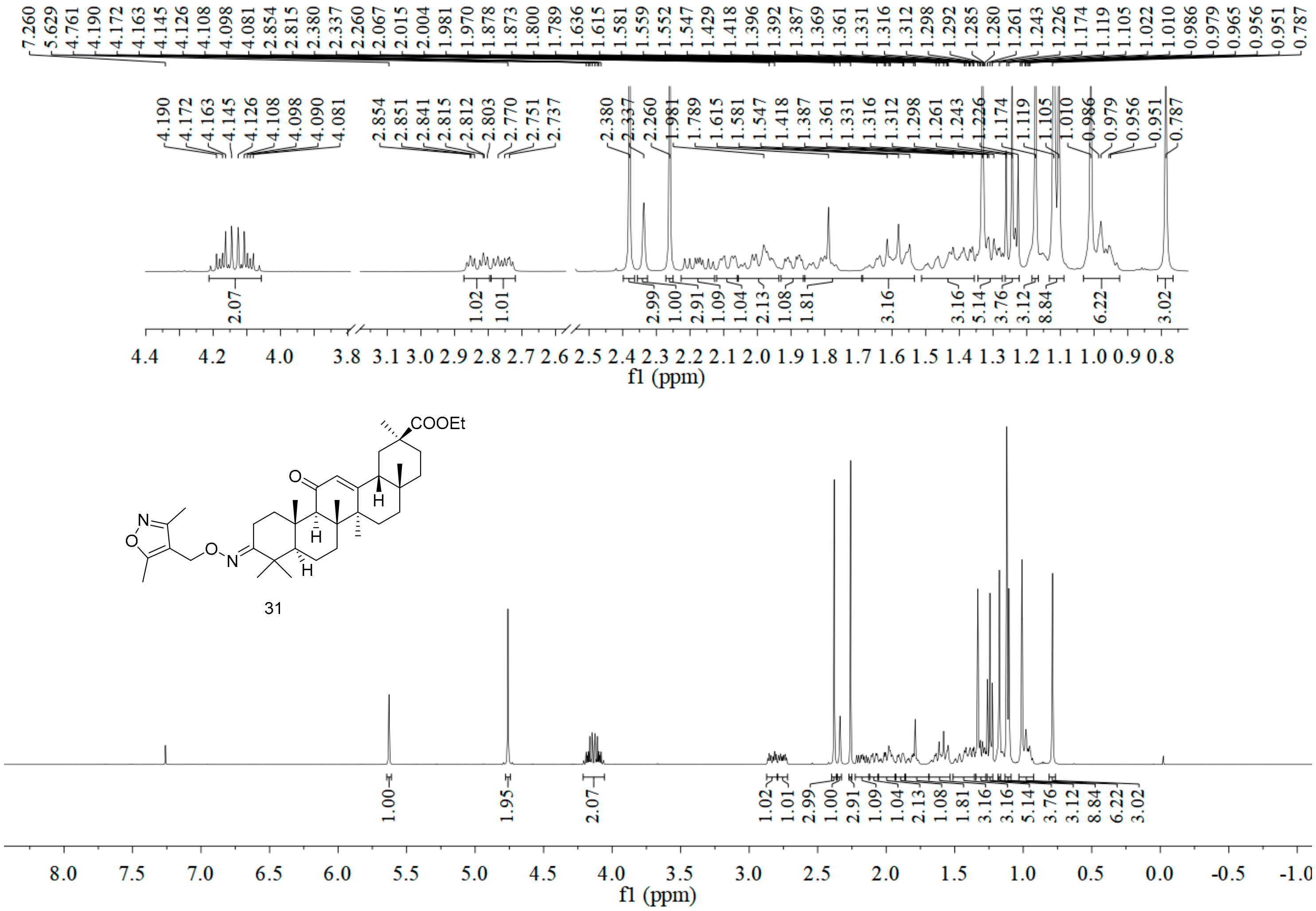

^{1}H NMR of Compound **31** (400 MHz, $CDCl_3$)

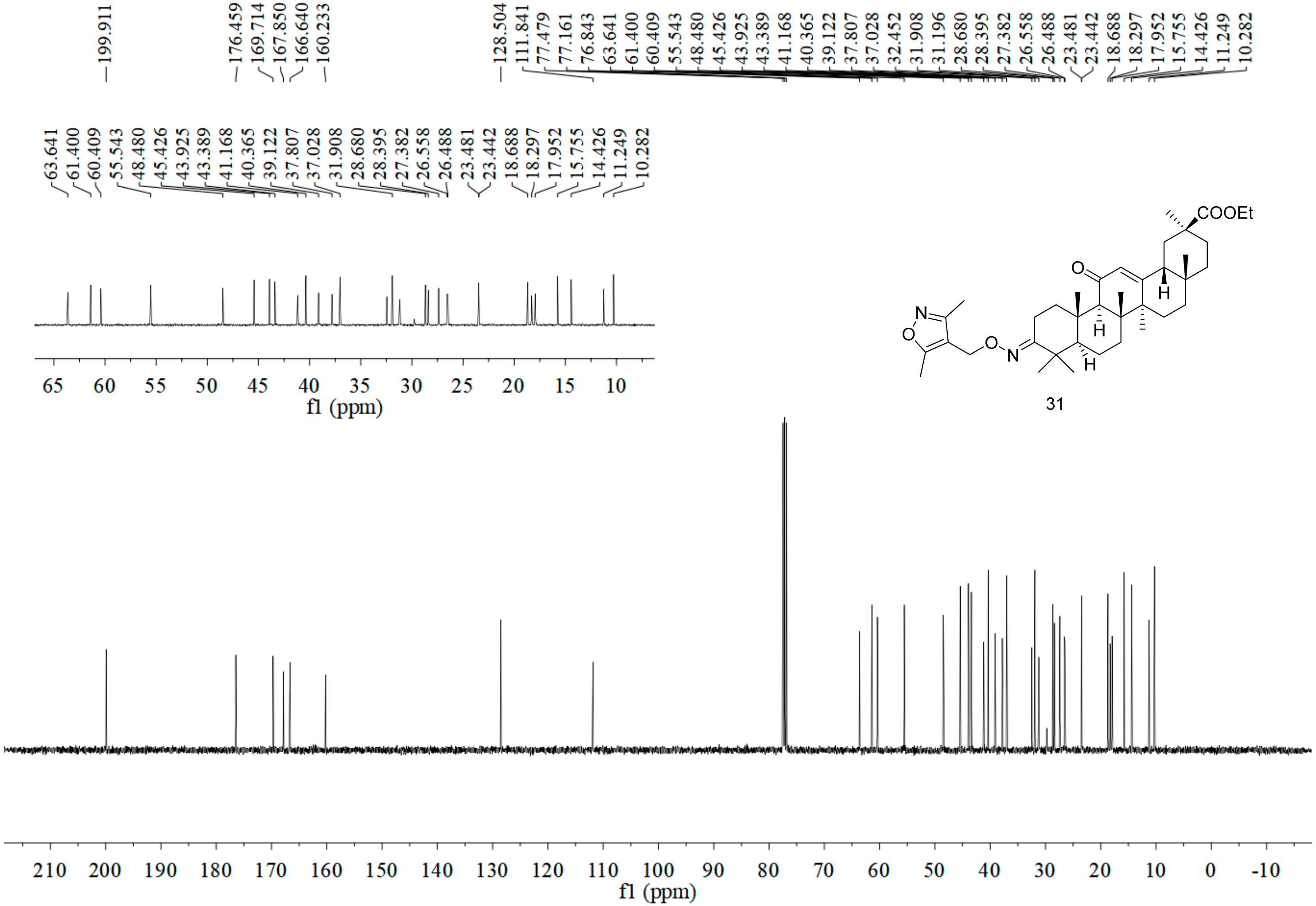

31

^{13}C NMR of Compound **31** (100 MHz, $CDCl_3$)

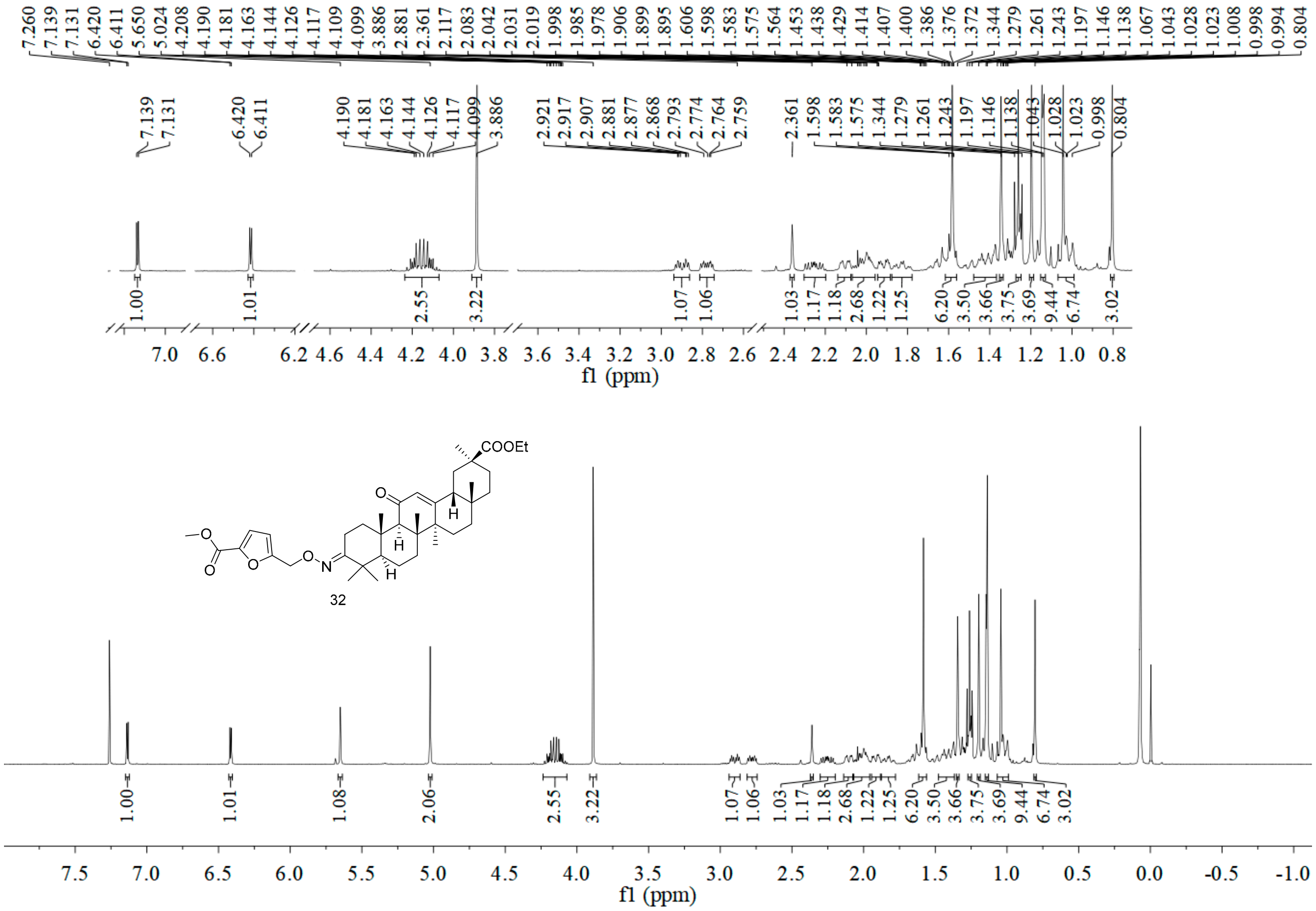

^{1}H NMR of Compound **32** (400 MHz, $CDCl_3$)

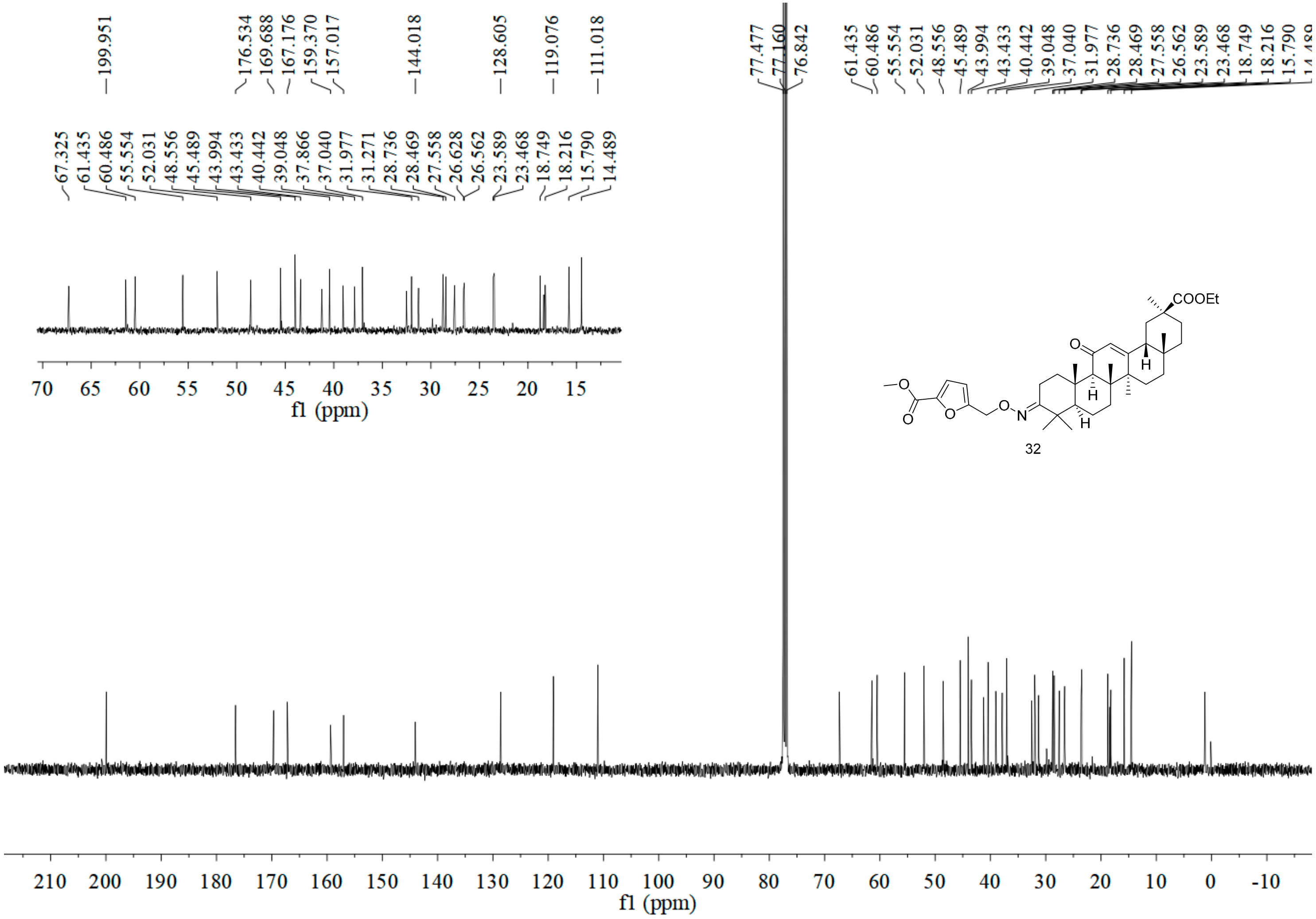

32

^{13}C NMR of Compound **32** (100 MHz, $CDCl_3$)

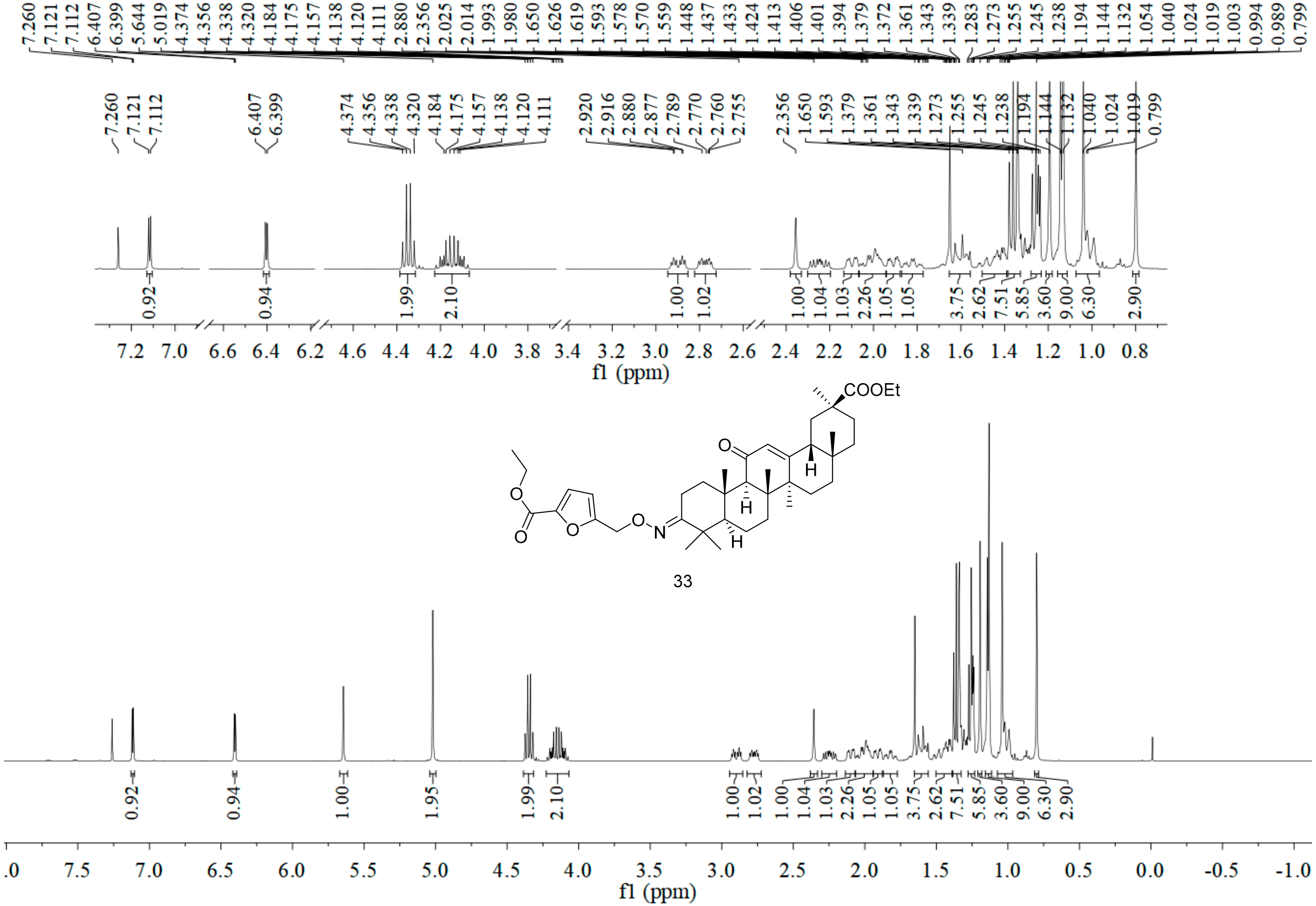

33

^{1}H NMR of Compound **33** (400 MHz, $CDCl_3$)

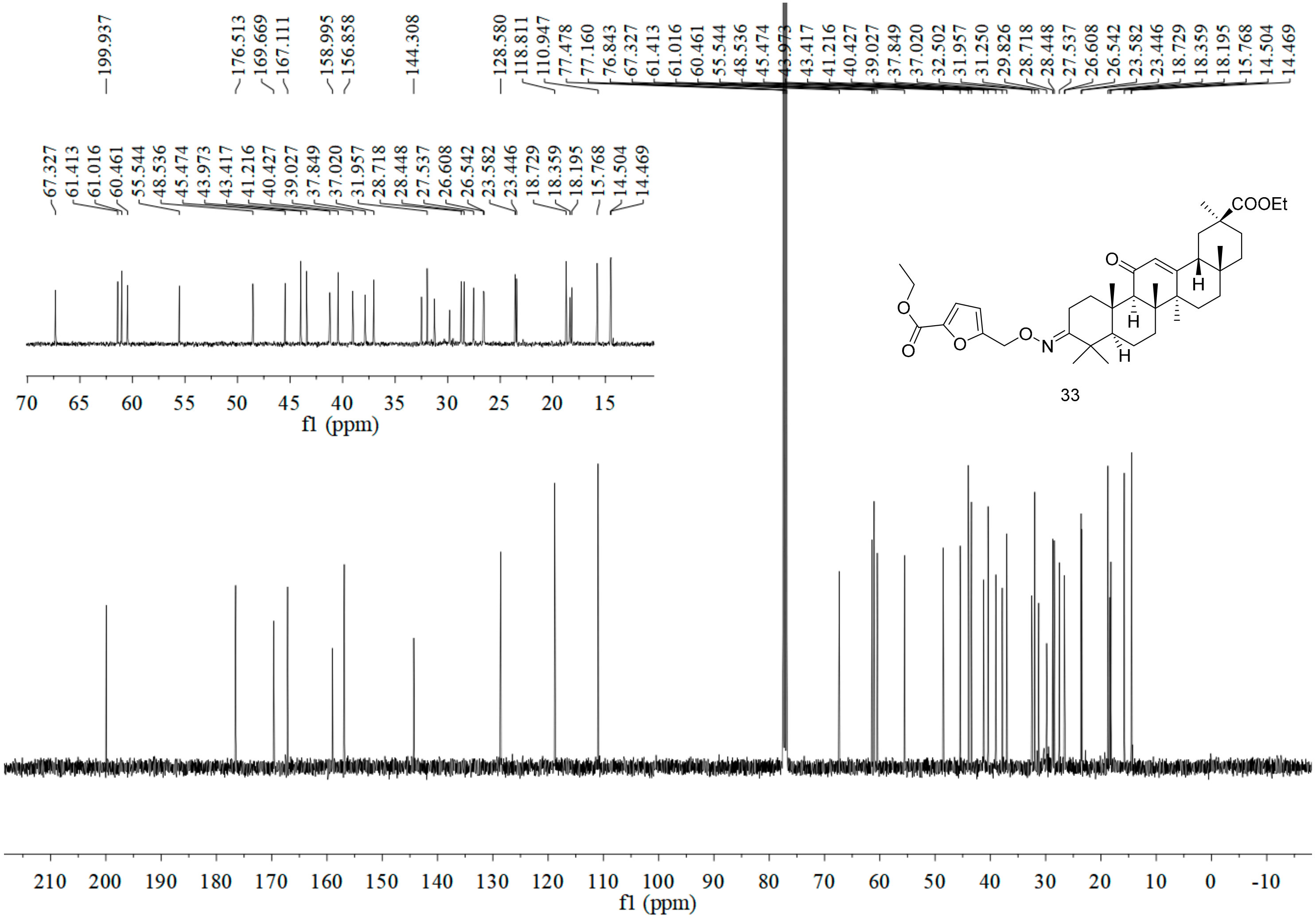

^{13}C NMR of Compound **33** (100 MHz, $CDCl_3$)

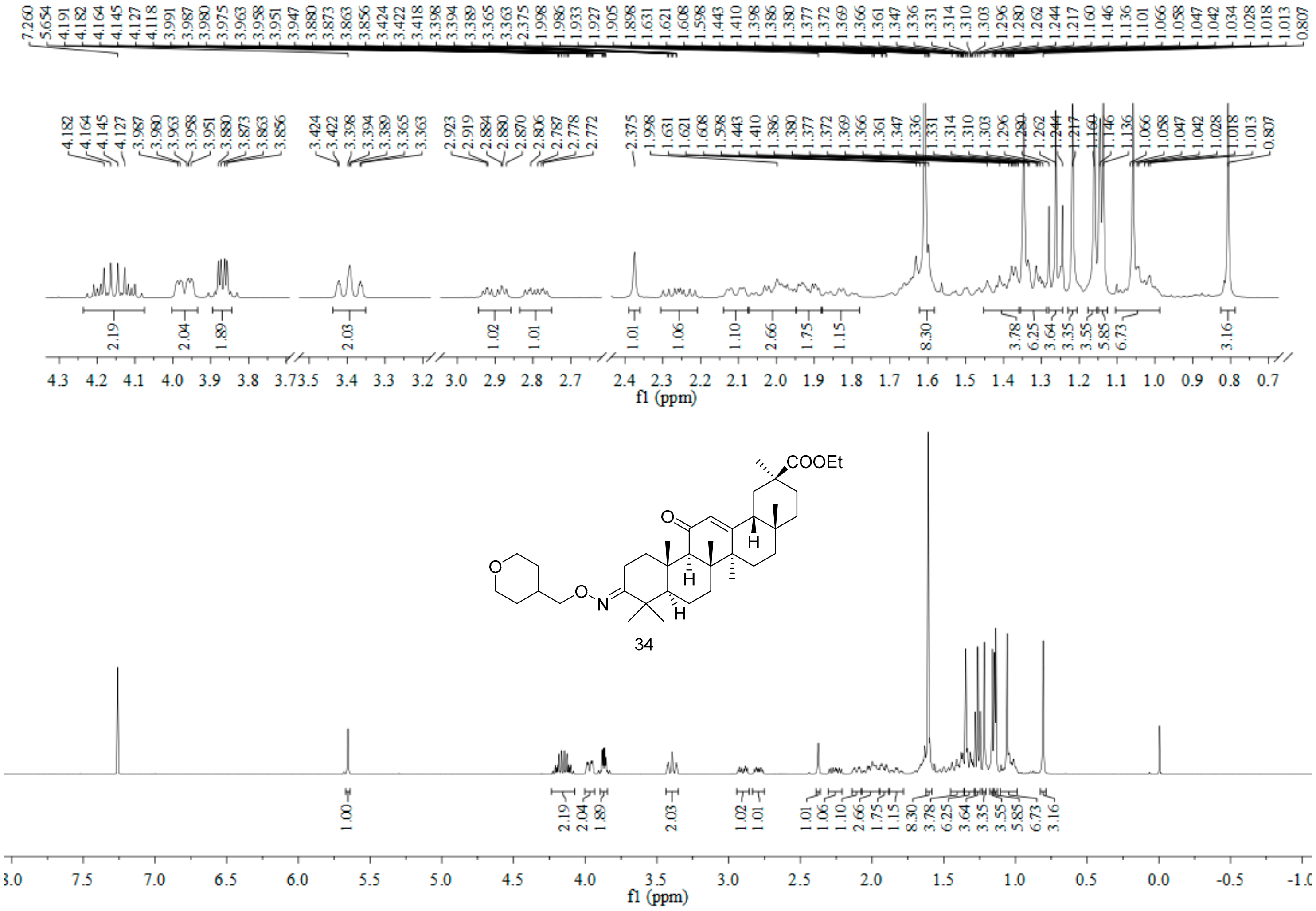

^{1}H NMR of Compound **34** (400 MHz, $CDCl_3$)

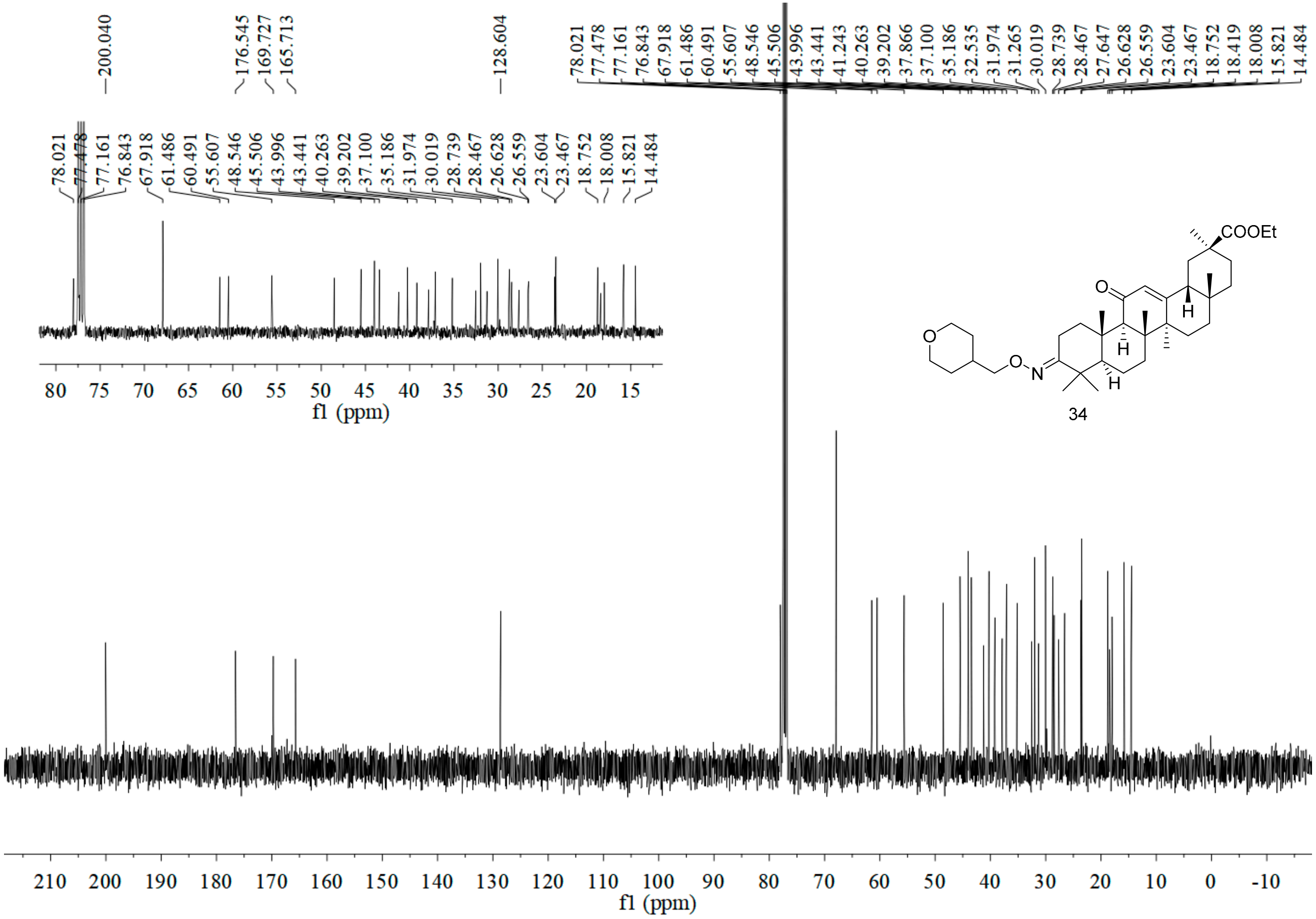

^{13}C NMR of Compound **34** (100 MHz, $CDCl_3$)

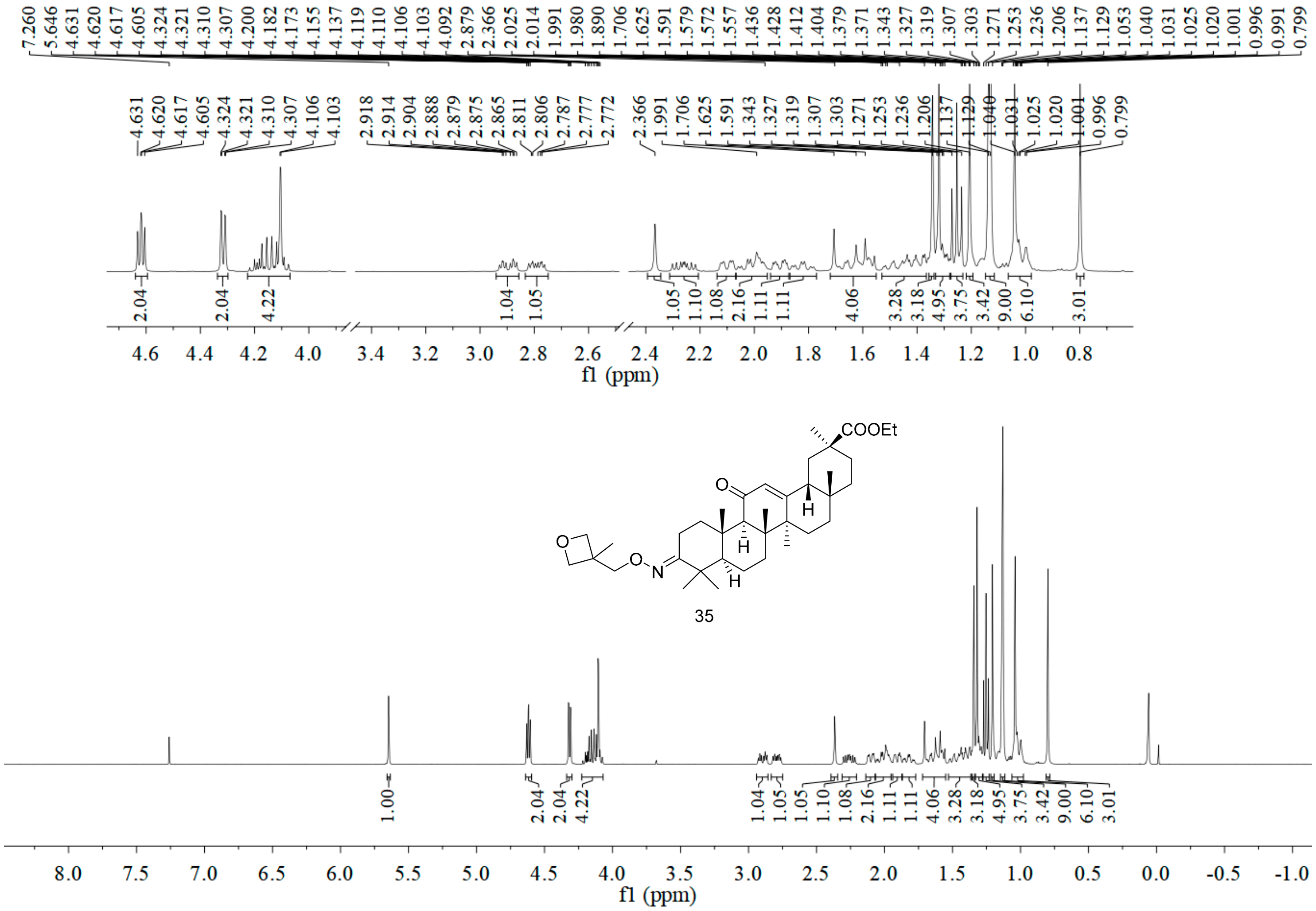

^{1}H NMR of Compound **35** (400 MHz, $CDCl_3$)

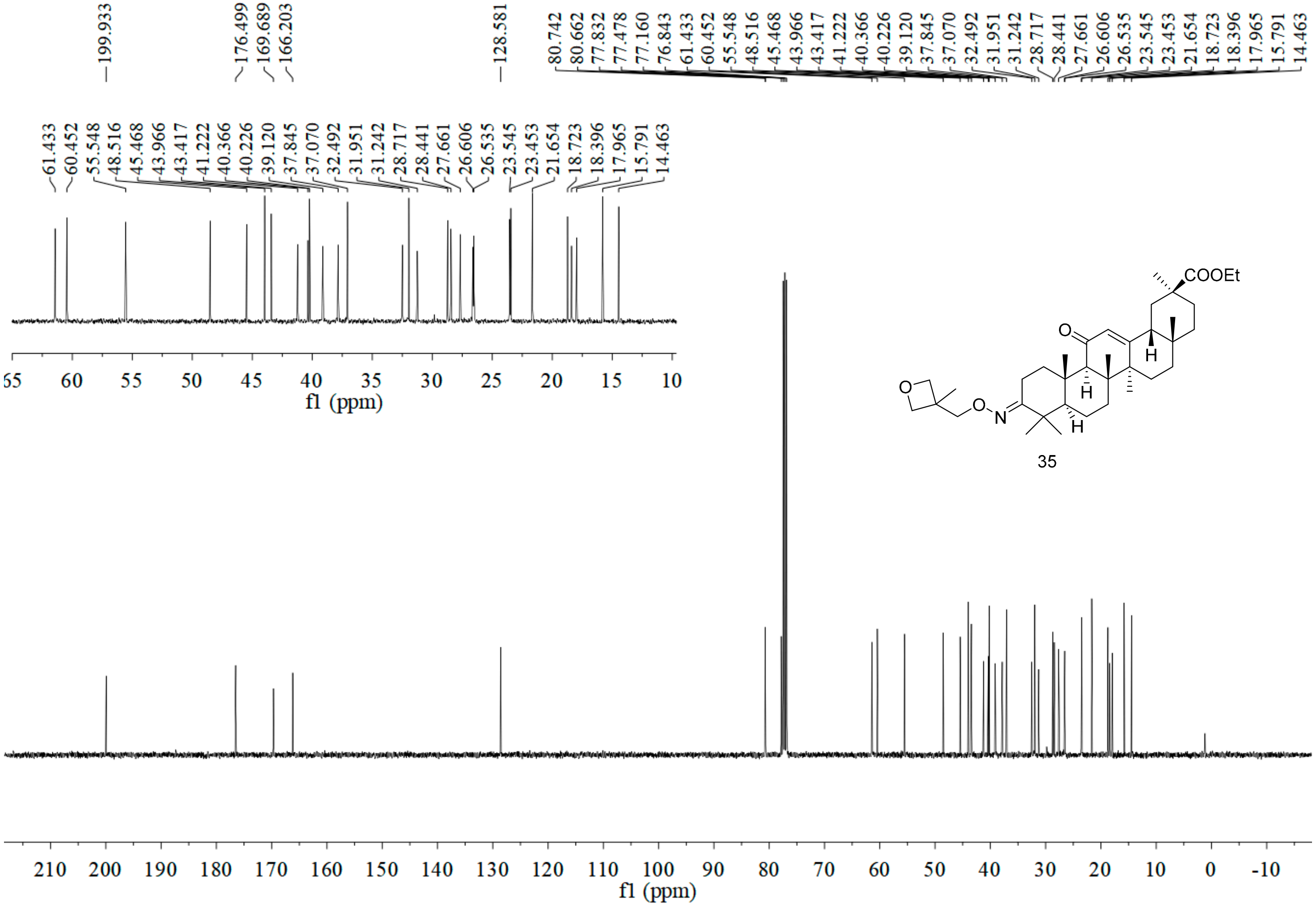

^{13}C NMR of Compound **35** (100 MHz, $CDCl_3$)
